# Variability within the 10-Year Pollen Rain of a Seasonal Neotropical Forest and Its Implications for Paleoenvironmental and Phenological Research

**DOI:** 10.1371/journal.pone.0053485

**Published:** 2013-01-08

**Authors:** Derek S. Haselhorst, J. Enrique Moreno, Surangi W. Punyasena

**Affiliations:** 1 Program in Ecology, Evolution and Conservation Biology, University of Illinois, Urbana, Illinois, United States of America; 2 Center for Tropical Paleoecology and Archaeology, Smithsonian Tropical Research Institute, Panama, Republic of Panama; 3 Department of Plant Biology, University of Illinois, Urbana, Illinois, United States of America; The Pennsylvania State University, United States of America

## Abstract

Tropical paleoecologists use a combination of mud-water interface and modern pollen rain samples (local samples of airborne pollen) to interpret compositional changes within fossil pollen records. Taxonomic similarities between the composition of modern assemblages and fossil samples are the basis of reconstructing paleoclimates and paleoenvironments. Surface sediment samples reflect a time-averaged accumulation of pollen spanning several years or more. Due to experimental constraints, modern pollen rain samples are generally collected over shorter timeframes (1–3 years) and are therefore less likely to capture the full range of natural variability in pollen rain composition and abundance. This potentially biases paleoenvironmental interpretations based on modern pollen rain transfer functions. To determine the degree to which short-term environmental change affects the composition of the aerial pollen flux of Neotropical forests, we sampled ten years of the seasonal pollen rain from Barro Colorado Island, Panama and compared it to climatic and environmental data over the same ten-year span. We establish that the pollen rain effectively captured the strong seasonality and stratification of pollen flow within the forest canopy and that individual taxa had variable sensitivity to seasonal and annual changes in environmental conditions, manifested as changes in pollen productivity. We conclude that modern pollen rain samples capture the reproductive response of moist tropical plants to short-term environmental change, but that consequently, pollen rain-based calibrations need to include longer sampling periods (≥7 years) to reflect the full range of natural variability in the pollen output of a forest and simulate the time-averaging present in sediment samples. Our results also demonstrate that over the long-term, pollen traps placed in the forest understory are representative samples of the pollen output of both canopy and understory vegetation. Aerial pollen traps, therefore, also represent an underutilized means of monitoring the pollen productivity and reproductive behavior of moist tropical forests.

## Introduction

The changing composition of fossil pollen in sediment profiles is used to reconstruct changes in vegetation and climate over time [1]–[4]. The interpretation of fossil samples, in turn, is based on modern pollen assemblages collected from ambient pollen and the mud-water interface of lakes from a range of ecosystems. Variation in the composition of these assemblages corresponds to differences in the floristic composition of the samples’ surrounding communities [5]–[14]. From these modern samples, pollen-climate calibration data sets have been developed for both temperate [12] and tropical [11], [13] ecosystems.

Despite the established relationship between pollen and vegetation, palynologists recognize that the structure of a pollen assemblage does not necessarily mirror the structure of the surrounding plant community. Studies in both temperate and tropical systems report that plant taxa may be over- or underrepresented due to differences in pollen dispersal and productivity, which varies by taxon and can be influenced by environmental conditions [10], [15]–[18]. This is particularly problematic for lowland tropical systems, which have a smaller number of lake sediment calibration studies from which to draw, and are more reliant on short-term studies of airborne pollen rain, most often collected over 1–3 years [7], [10], [15], [16], [19]. This is in contrast to surface sediment samples, which reflect a time-averaged accumulation of pollen spanning several years or more [20]. The relatively short timeframes represented by aerial pollen samples suggest that many pollen rain studies do not capture the full range of natural variability in pollen rain composition and abundance and may not provide the time-averaged data needed to calibrate the relationship between pollen, vegetation, and climate effectively [20]. Given that tropical fossil pollen assemblages often reflect extended periods of pollen accumulation, the current use of pollen rain data in the development of tropical pollen transfer functions for paleoclimate reconstructions may be highly biased by the effect of fine-scale climatic variation on pollen productivity.

Few pollen rain studies have measured the extent of the variability of tropical pollen production over time periods comparable to sediment samples, such as ten or more years [20]; a rare example is [21]. Due to the irregularity of flowering periodicity in many tropical taxa [22], short-term studies are unlikely to capture the full range of interannual variation in pollen production, particularly in high-diversity lowland tropical systems. As a result, pollen-climate correlations underestimate year-to-year variability in pollen outputs among even the most commonly sampled plant taxa, and overemphasize the degree of taxonomic turnover at a single site.

To establish the interannual variability of pollen outputs for one tropical forest, we sampled ten years of the seasonal pollen rain from Barro Colorado Island (BCI), Panama, using aerial pollen traps. Vertically stratified within the forest canopy, these traps captured seasonal pollen influx at varying heights for one site, the Lutz weather tower. The results provide a dynamic and long-term assessment of the modern pollen rain of a Neotropical forest, which is in contrast to more traditional static correlations of pollen assemblages to community composition [8], [9]. The data demonstrate a large amount of variability in both seasonal and interannual pollen production among the most common pollen taxa, despite a relatively stable forest community, suggesting that pollen influx may be influenced as much by climatic conditions as the changing community structure of a forest. The influence of both the current and previous year’s climate were evident in the changing pollen rain composition. Our results also highlight the seasonality of the reproductive output of prominent Neotropical plant taxa and demonstrate how aerial pollen traps can be used to track these aspects of plant reproduction.

## Materials and Methods

### Ethics Statement

Airborne pollen was collected using aerial pollen traps placed on the Lutz tower at BCI, Panama. Permission to place these traps was granted by the Smithsonian Tropical Research Institute (STRI) and a collecting permit was issued by the Autoridad Nacional del Ambiente of Panama (Permit No. SEX/P-3-12).

### Study Site

Our study site is the seasonal tropical moist forest of BCI, Panama (9°9′ N, 79°51′ W), where plant community dynamics have been studied over the past 30 years [23], [24]. There is a distinct dry season usually beginning in mid-December and ending in mid-April that is marked by decreased precipitation and a 40–50% increase in solar irradiance [25], [26]. During the dry season, evapotranspiration can exceed precipitation, resulting in a period of severe water stress and leaf loss in many plant species [22], [27], [28]. The island receives ∼2600 mm of rainfall per year with severe dry seasons receiving as little as 100 mm of the annual total [25]. Seasonal climate variation can become further accentuated by the El Niño-Southern Oscillation (ENSO) [25], which provides a natural experiment for studying short-term plant physiological and reproductive responses [29]–[31].

### Pollen Rain Dataset

Pollen rain samples were collected seasonally from 1996–2005. Aerial pollen traps (following [32]) were placed on the BCI Lutz weather tower at 5 m intervals from ground level (0 m) to above the canopy (42/45 m; in 2002, the height of the tower was increased and the trap positioned at 42 m was moved to 45 m). Traps were extended one meter away from the base of the tower using PVC tubing to prevent disturbance by other tower users. Pollen traps were placed on the tower at the beginning of the dry and wet seasons and were collected at the beginning of the next season, with one exception: dry season traps could not be collected at the onset of the wet season in 1998. As a result, the 1998 samples reflect a single annual sampling period. There is variation in the absolute timespan represented by each pollen trap due to the differences in the trap set and collection dates. The mean length of the dry and wet season sampling periods were 137 (σ = 13) and 220 (σ = 24) days, respectively.

We chose a subset of samples (0, 5, 20, 25, 40 and 42/45 m) for our analysis. This allowed us to compare pollen influx from the understory, (0, 5 m), upper canopy, (20, 25 m) and emergent (40, 42/45 m) sampling heights in a similar manner to [21]. Dry and wet season samples were counted and analyzed from each of the six sampling heights, bringing each combined annual total (with the exception of 1998) to 12 samples. Six samples were analyzed from 1998, for a 10-year total of 114 samples.

Samples were processed to remove organic debris and isolate pollen following standard protocols [32]–[34], with minor modifications (see supplemental file Appendix S1). At the end of each sampling period, traps were replaced on-site and the rayon fiber and Whatman (GF/D) filter lining trap funnels were transferred to Whirl-Pak bags. After collection, samples were stored at a temperature of 4°C at the Center for Tropical Paleoecology and Archaeology in Panama City, Panama.

One tablet containing a known quantity of exotic *Lycopodium* spores was dissolved in a solution of 10% HCl, neutralized in a test tube with distilled water, then added to each sample to calculate pollen influx [35]. A solution of 5% KOH was added to each sample to dissolve organic debris and free pollen from the rayon fiber and filter. Samples were then manually squeezed, passed through a 500 µm copper mesh, centrifuged at 2700 rpm for 5 minutes, washed with distilled water, and centrifuged again as needed. Samples were then dehydrated using glacial acetic acid prior to an acetolysis treatment consisting of a 9∶1 mix of acetic anhydride ((CH_3_CO)_2_O) and sulfuric acid (H_2_SO_4_). After treatment, residues of the processed pollen were mounted on microscopic slides using a jelly glycerin mounting media.

### Pollen Counts and Identifications

Counts were completed from prepared microscopic slides using a transmitted light microscope at 400× magnification [33]. Counts were made following vertical transects from the center of each slide until ≥300 identified grains had been tallied. The remainder of the slide was scanned for unique pollen types. Preliminary pollen identifications were made using published photographs and morphological descriptions of species present on BCI [36]. Final pollen identifications were based on comparisons to reference material (the Alan Graham pollen reference collection, housed at the Smithsonian Tropical Research Institute (STRI)). We identified 133 unique morphotypes. Pollen types were identified to species when possible; however, due to the highly conserved morphology of related pollen types [37], the majority of identifications were limited to the family or genus level (Table S1). If a taxonomic identification could not be made, we designated the specimen as an unknown morphotype. We recognized 11 morphologically distinct unknown taxa. Pollen types that shared morphological similarities with previously described grains, but for which the identities remained uncertain, were described as *cf*. species. Corresponding pollen type photographs were taken using a 63× oil immersion objective (NA 1.40) (Plates S1–S22 in Appendix S2, Plates S23–S45 in Appendix S3).

Pollen influx was estimated using the count of exotic *Lycopodium* spores recorded in each sample. To account for temporal variability within the year-to-year seasonal sampling methodology, both seasonal and annual pollen influxes were normalized by the number of days pollen samples were collected (pollen grains/cm^2^/day) as opposed to more traditional measures of annual pollen influx (pollen grains/cm^2^/year). Three samples (2001 dry 5 m; 2002 wet 40 and 45 m) were excluded from subsequent analyses of the pollen influx because of the limited amount of pollen in these samples. Pollen counts of ≥300 identified grains could not be obtained from these slides.

### Vegetation Census

We completed a vegetation census of the area within a 50 m radius (0.785 ha) of the Lutz tower in May 2011 to document the floristic composition of the immediate surrounding forest. Our surveyed radius was based on previous research from BCI indicating that the influx of dominant pollen types to aerial traps likely originates within a 30–40 m radius of traps [15], [16]. Reproductive stems of all tree, shrub, liana, and herbaceous individuals were tallied in 5 m and 20° intervals from the base of the tower. Estimates of basal area (BA; m^2^/ha) were calculated in 40 plant taxa for which DBH (diameter at breast height) measurements at or above reproductive size were made.

The census represents a snapshot in time, so we are unable to account for the possibility of annual turnover of vegetation in our analysis. However, because the average residency time of a tree in the BCI canopy is ∼45 years and no large-scale floristic changes were observed near Lutz tower during our sampling period, this single survey served as an approximate baseline of the surrounding plant community for the entire decade.

### Meteorological Dataset

Meteorological data for BCI were available for the same timeframe as the pollen dataset [38]. Averaged values for each climate variable were calculated based on the respective durations of each sampling period. Climate variables investigated include: evapotranspiration (ET; mm/day); mean photosynthetically active radiation (PAR; Einsteins/m^2^/h); mean precipitation (mm/day); proportion of days receiving no rainfall; and temperature (minimum, maximum, mean, and mean diurnal range; °C).

### Pollen Influx and Forest Composition

The ratio of relative pollen influx to relative basal area (R-rel; measured as the percentage of the total number of pollen grains of a taxon in sample divided by the percentage basal area of that taxon) provides a metric for assessing the degree to which different plant taxa are overrepresented or underrepresented in a pollen sample [16], [33], [39]. Plant taxa present within 50 m of the Lutz tower, but absent from the 10-year pollen record, are ‘silent taxa’ (Table S2) [16]. Pollen taxa corresponding to plant species within the surveyed vegetation radius are referred to as ‘local pollen taxa,’ and pollen taxa unaccounted for by species within the surveyed radius, ‘non-local pollen taxa.’ R-rel values >1 indicate pollen types that are overrepresented with respect to the surrounding vegetation and values <1 indicate types that are underrepresented.

Fifty-nine of the 113 species identified in the vegetation census are represented by pollen types in the 10-year pollen rain (Table S2). This corresponds to 39 pollen taxa (Table 1). We calculated the ten-year aggregate R-rel values for 24 of these taxa, where BA estimates could be estimated from DBH measurements (Table 1). To estimate the degree of overrepresentation and underrepresentation of common Neotropical pollen taxa without measured DBH and BA estimates, we also calculated the ten-year pollen/vegetation (p/v) estimates for all 39 taxa (Table 1); p/v is measured as the percentage of the total number of pollen grains of a given taxon divided by the corresponding percentage of reproductive stems for each taxon. To illustrate how variable year-to-year representation of different pollen types can be, we also calculated ten annual measures of R-rel and p/v by combining pollen counts from both the wet and dry season for each of the ten years.

**Table 1:** Representation of pollen taxa with parent plants in the surrounding community.

Taxon	Life form	Stems %	Pollen %	BA %	R-rel ± σ	p/v ± σ
Moraceae/Urticaceae	tree	3.10	17.6	2.34	7.7±4.0	4.5±2.3
*Cecropia*	tree	1.00	11.9	0.21	59.4±26.8	11.8±5.3
Arecaceae	tree	0.67	7.49	0.28	25.0±11.4	11.2±5.1
*Maripa*	liana	0.08	4.17	–	–	49.7±28.9
Melastomataceae	shrub	1.76	3.86	–	–	2.2±0.9
cf. *Gustavia*	tree	2.18	3.10	3.08	1.0±0.5	1.4±0.7
*Faramea*	shrub	3.60	2.15	0.23	10.7±9.0	0.6±0.5
*Eugenia*	tree	0.08	2.13	0.36	5.3±4.8	25.4±22.7
*Virola*	tree	0.33	1.50	7.19	0.2±0.1	2.2±0.6
*Acalypha*	herb	0.33	1.49	–	–	4.4±2.0
*Alseis*	tree	1.01	1.47	1.84	0.8±0.5	1.5±0.9
*Hyeronima*	tree	0.33	1.44	1.57	0.9±0.4	4.3±2.0
*Anacardium*	tree	1.01	1.22	34.6	0.03±0.01	1.2±0.6
*Uncaria*	liana	1.01	1.06	0.34	3.5±1.8	1.05±0.5
*Pseudobombax*	tree	0.08	1.03	3.26	0.3±0.1	12.3±5.5
*Spondias*	tree	0.17	0.99	2.87	0.3±0.1	5.9±2.0
*Protium*	tree	1.26	0.98	5.22	0.2±0.2	0.8±0.7
Malpighiaceae	liana	3.02	0.65	–	–	0.2±0.2
*Schefflera*	tree	0.08	0.36	0.35	1.2±0.8	4.4±2.9
*Solanum*	vine	0.08	0.32	–	–	3.9±2.3
*Arrabidaea*	liana	0.17	0.31	0.07	3.1±2.1	1.8±1.2
*Astronium*	tree	0.08	0.30	–	–	3.5±1.7
*Cydista*	liana	0.08	0.29	–	–	3.5±3.9
Combretaceae	tree/liana	0.08	0.28	–	–	3.3±2.3
*Ficus*	tree	0.25	0.24	11.2	0.02±0.02	0.9±1.2
*Psychotria*	shrub	11.7	0.24	–	–	0.02±0.02
*Paullinia*	liana	0.17	0.23	–	–	1.4±0.9
*Quassia*	shrub	0.67	0.22	–	–	0.3±0.25
Piperaceae	shrub	33.1	0.19	–	–	0.01±0.01
*Bombacopsis*	tree	0.08	0.16	–	–	1.9±1.2
*Socratea*	tree	0.25	0.15	0.17	0.7±0.5	0.6±0.4
*Casearia*	tree	0.42	0.08	–	–	0.2±0.2
*Posoqueria*	shrub	0.50	0.08	0.1	0.8±1.3	0.15±0.3
cf. *Inga* sp.	tree	0.42	0.05	0.1	0.5±0.8	0.1±0.2
*Lacmella*	tree	0.08	0.05	0.2	0.3±0.2	0.6±0.5
*Pouteria*	tree	0.08	0.04	0.1	0.4±0.3	0.5±0.3
*Erythrina*	shrub	0.25	0.03	0.1	0.3±0.3	0.1±0.1
*Mendoncia*	vine	0.17	0.01	–	–	0.03±0.08
*Quararibea*	tree	0.33	.003	2.70	.001±.007	.01±.05

Calculations of basal area (%BA), R-rel, and p/v for taxa found in both the pollen samples and the vegetation census. Relative pollen influx provided by “% Pollen”, based on the relative representation of each taxon in the full ten-year pollen record. Relative abundance and basal area of individual taxa within 50 m given by “% Stems”, based on the May 2011 census (Table S2). R-rel and p/v values were calculated annually and are shown as averages and one standard deviation (σ).

### Pollen Influx and Environmental Variability

Canonical correspondence analysis (CCA) was used to assess the degree to which variation in composition among the seasonal pollen samples correlated with height within the forest canopy and eight time-averaged climate variables (described in “Meteorological Dataset”). Seasonal pollen influx (pollen grains/cm^2^/day) for the 50 most abundant pollen types from all pollen samples were included in the analysis, with the exception of the three samples for which a 300-grain baseline could not be counted (2001 dry 5 m; 2002 wet 40 and 45 m). Rare pollen types (defined as contributing <0.20% of the 10-year pollen rain) were excluded from the ordination.

A second CCA of annual pollen influxes (using combined dry and wet season pollen influxes divided by the total number of days collected) was also completed to assess the effect of year-to-year environmental variability on the pollen influx. Environmental data from both the concurrent and previous sampling years were included. Separate CCA analyses were completed using the 50 most abundant pollen taxa and the 24 of those 50 taxa that were non-local (not censused within 50 m of Lutz tower) to assess how climate influences the pollen outputs of local and non-local taxa.

The significance of the canonical axes was evaluated through 1000 permutations of the ordination matrix. Raw environmental and species scores were plotted and compared for the first two ordination axes. Variables with the greatest absolute CCA scores accounted for most of the seasonal or interannual variability in pollen production.

### Measuring Compositional Stability

We used correspondence analysis (CA) to measure the degree of variation in the composition of the pollen influx introduced by sampling length. To do so, we created aggregate, time-averaged samples using a moving window of varying sampling lengths: one, three, five, seven, and 10 years. The aggregate samples were continuous in time and, as with our other influx data, normalized by the number of days in the sampling period. We aggregated each sampling height separately and restricted our analysis to the same 50 pollen types that were included in the seasonal CCA analysis.

## Results

### Pollen Influx

In total, 34,299 individual pollen grains were recorded and identified from 114 seasonal pollen rain samples. Mean pollen influx to each sample was 28.1 pollen grains/cm^2^/day. The mean seasonal influx to each sampling height did not vary significantly between dry and wet sampling periods, but was instead most influenced by sampling height, with samples take above the canopy with the lowest pollen influx (Table S3). Maximum pollen influx was calculated in the 2000 5 m dry season sample with 230.1 pollen grains/cm^2^/day and the lowest pollen influx was recorded for the 2005 45 m wet season sample, with only 0.19 pollen grains/cm^2^/day. Pollen types corresponding to local taxa (species located within the 50 m censused radius) accounted for 67.6% of the time-averaged 10-year pollen influx, with a maximum influx of local pollen taxa to any one sample in the 1999 wet 45 m sample (90.5%), and lowest local influx in the 1999 dry 25 m sample (35.7%).

We observed a few long-distance dispersed grains (grains originating from outside the Canal Zone area, characteristic of montane taxa, e.g. *Alnus* and saccate grains – putatively *Podocarpus*). The highest percentages of long-distance dispersed pollen were found in the 40 and 42/45 m pollen samples, but never exceeded 1% of the relative pollen influx in any sample for which a 300-grain baseline was counted. The maximum percentage observed was 0.9% in the 2003 dry 45 m sample. We also observed single grains of *Alnus* and gymnosperm pollen types in samples collected at both 0 and 5 m, but they never exceeded more than trace proportions of the total influx.

### Compositional Stratification

**Figure 1:**
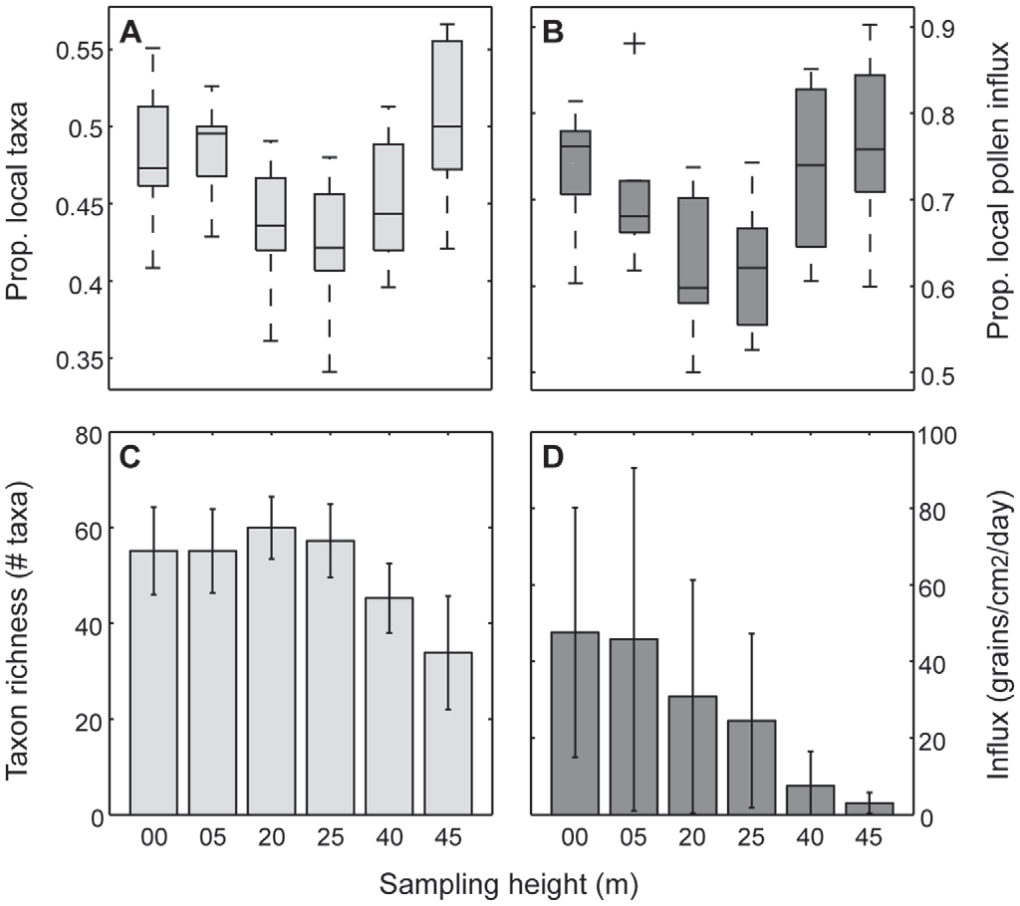
Contribution of local taxa to pollen assemblage richness and influx as a function of sampling height. Boxplots of proportion of pollen taxa in the ten annual aggregate samples that correspond to local species, those within 50 m of Lutz tower, are shown in (A) and the proportion of pollen influx that corresponds to local species, is shown in (B). Influx is measured as the number of grains/cm^2^/day. Median values are designated by horizontal bars. The edges of the box are the 25th and 75th percentiles. Whiskers represent +/−2*σ* or 95% of the data. Outliers are plotted as single points. The number of pollen taxa recorded at each height is shown in (C) and the annual pollen influx for each sampling height is shown in (D). Error bars for (C, D) represent one standard deviation.

The composition of annual pollen influx to each sampling height was highly variable (Figure 1). The proportion of the pollen influx accounted for by non-local species (located outside of the surveyed radius) was less than that of local components (Figure 1b), most notably at the understory and emergent sampling heights. A two-sample t-test comparing the contributions of local and non-local pollen taxa to the ten-year aggregate of pollen influx (pollen grains/cm^2^/day) revealed that local pollen taxa (taxa found within 50 m) contributed significantly more to influx to the pollen rain than did pollen components originating at a distance beyond 50 m (t = 3.02; *P* = 0.002). The proportion of the influx accounted for by local pollen taxa was greatest in the emergent 40/45 m traps (Figure 1a,b); however, the total pollen influx in the emergent traps (40, 42/45 m) was consistently lower than the upper-canopy (20, 25 m) and understory (0, 5 m) traps (Figure 1d). The strong local signature in these traps can be attributed to a high relative influx of Melastomataceae (9.8%), cf. *Gustavia* (5.8%) and *Hyeronima* (3.4%) pollen types at these sampling heights in addition to anemophilous Moraceae/Urticaceae (22.6%) and *Cecropia* (20.5%). Although not all this pollen necessarily originated from within 50 m of the pollen traps, all five taxa are represented within the censused radius.

Influx from non-local pollen taxa was greatest in upper-canopy 20/25 m sampling heights. The difference between local and non-local pollen influx was not significant at 20 m (t = 0.774; *P* = 0.446) or at 25 m (t = 0.843; *P* = 0.398). Furthermore, pollen assemblages from the 20/25 m sampling heights were marginally, but not significantly, more taxon-rich than understory pollen assemblages (Figure 1c). A one-way ANOVA comparing pollen taxon-richness between understory and canopy sampling heights revealed negligible differences in species richness (*F*_3, 36_ = 0.767; *P* = 0.520). The increased non-local signature in the 20 and 25 m pollen assemblages instead was driven in part by increased relative representation at these sampling heights from *Schefflera* (3.9%), *Machaerium* (3.9%), *Cordia* (2.4%) and *Sabicea* (2.4%), pollen types not found within the censused radius, but local to BCI. Pollen diagrams for the 20 most abundant taxa at each sampling height are provided in Figure S1.

### Forest Composition and Pollen Representation

The vegetation census recorded 114 different species within the 50 m radius surrounding the Lutz tower, which includes 45 families and 92 genera (Table S2). A total of 1193 individuals were recorded, half of which are species of *Piper* (33.1%) and Rubiaceae (21.6%). In terms of basal area, *Anacardium excelsum* (Anacardiaceae) and *Ficus insipida* (Moraceae) were the most prominent woody species (Table 1). Other notable taxa include *Virola* (Myristicaceae), *Protium* (Burseraceae), *Gustavia* (Lecythidaceae), *Pseudobombax* (Malvaceae), *Spondias* (Anacardiaceae), and *Quararibea* (Malvaceae) (Table 1). A summary of the vegetation census is included as a supplemental file (Table S4).

There was limited overlap between the censused vegetation and the pollen trap composition. Fifty-nine of the 114 recorded species were potentially found in the pollen samples (Table S2), representing 39 of the 133 identified pollen taxa. Multiple species comprise grouped pollen types in Arecaceae, Moraceae/Urticaceae, Piperaceae, and *Psychotria*. R-rel and p/v estimates indicated that many pollen taxa are either underrepresented (<1) or overrepresented (>1) in the 10 years of the pollen rain sampled (Table 1). Taxa such as *Anacardium* and *Ficus*, while dominant components of the measured basal area, are underrepresented with R-rel values well under one (Table 1). Anemophilous pollen types were predictably overrepresented in the pollen record, with Moraceae/Urticaceae (excluding *Ficus* (Moraceae) and *Cecropia* (Urticaceae)) contributing disproportionately more to the pollen influx than other taxa with comparable BA and reproductive stems (Table 1). However, some insect-pollinated taxa were also overrepresented, most notably Arecaceae (identified as *Astrocaryum* and *Oenocarpus* pollen types) and Melastomataceae pollen types. Several liana taxa such as *Arrabidaea*, *Maripa panamensis*, and *Uncaria* were also overrepresented. However, this association was not true for all liana species. For example, Malpighiaceae and *Hiraea*-type pollen were underrepresented.

**Figure 2:**
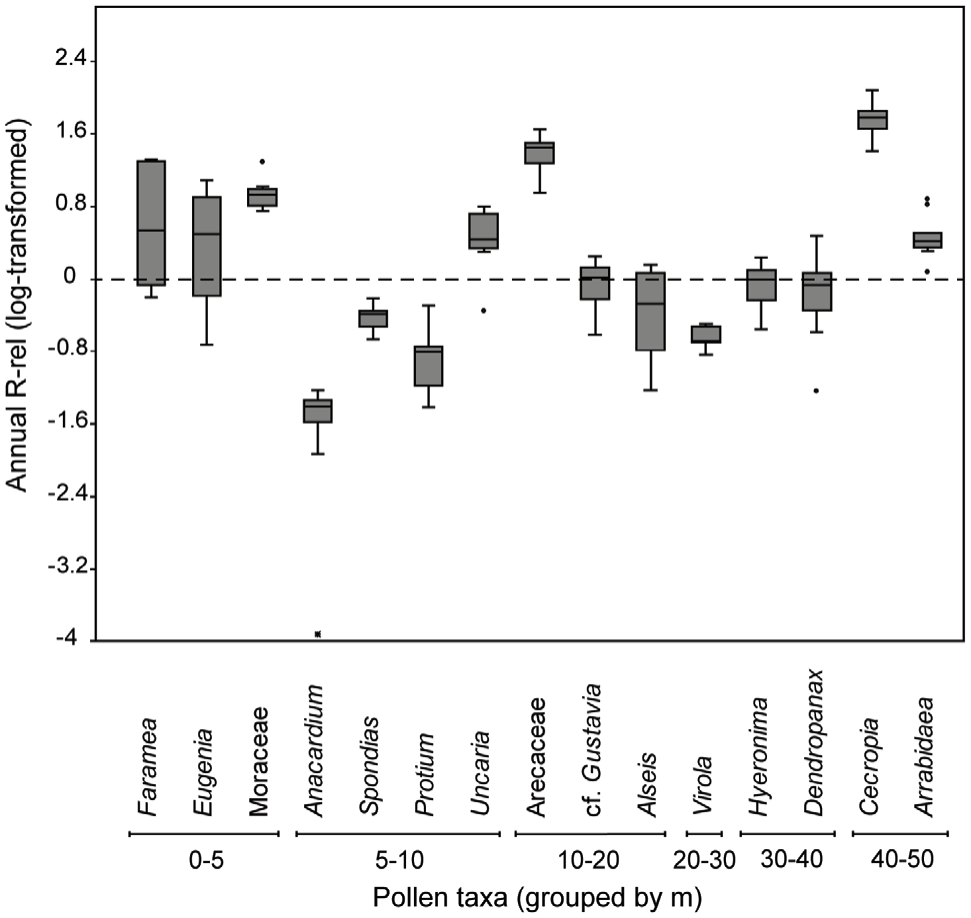
Annual variability in R-rel from 1996–2005. Variability in log-transformed annual R-rel values for 15 of the most abundant pollen taxa with corresponding basal area estimates. Taxa with values >0 are overrepresented and values <0 are underrepresented. Taxa are grouped according to distance to nearest individual at reproductive DBH from the base of Lutz tower (in m). Taxa within 5 m of the traps demonstrate some of the highest and most variable R-rel values.

Because of the small clearing surrounding the tower, there are few individual trees within 5 m of the traps. Three insect-pollinated species, *Eugenia coloradoensis*, *Faramea occidentalis*, and *Maripa panamensis*, were found within a 5 m radius and were likely overrepresented due to their proximity to the pollen traps (Table 1; Figure 2). Additionally, an individual of *Poulesnia armata* (Moraceae) was also recorded within 5 m, though the overrepresentation of this taxon was expected given its anemophilous pollination syndrome. However, not all taxa found within 5 m of Lutz were overrepresented. Taxa such as *Calathea* (Marantaceae), *Hasseltia* (Salicaceae), and *Xylopia* (Annonaceae) were completely absent from the 10-year pollen record, whereas taxa such as *Erythrina costaricensis* (Fabaceae-Papilionoideae) were highly underrepresented (Table 1). These species have relatively thin pollen walls and their absence may be a result of fungal degradation.

### Annual Variability in Pollen Representation

Annual calculations of R-rel revealed a high degree of year-to-year variability in the pollen output of reproductive individuals (Figure 2). *Faramea occidentalis* and *Eugenia coloradoensis*, while overrepresented in the aggregated 10-year pollen influx, also demonstrated strong year-to-year variability in annually calculated R-rel values. *Faramea* was an underrepresented pollen component in 1999 (R-rel = 0.61), but was overrepresented in 2000 (R-rel = 20.5). Similar year-to-year variability characterized the 10-year pollen influx of *Eugenia* (1996 R-rel = 0.28; 1997 R-rel = 12.2). Together, these two taxa accounted for 4.30% of the 10-year pollen rain, which is a relatively large portion of the pollen influx. Strong year-to-year variability in pollen overrepresentation and underrepresentation was not observed for all pollen taxa located within 5 m of Lutz; *Maripa panamensis* was overrepresented in each of the 10 years of the Lutz pollen rain sampled, with an annually estimated p/v value never dropping below 10.2.

Year-to-year variability in pollen representation decreased in plant taxa beyond 5 m of pollen traps. Even at a distance of 5–10 m, large canopy trees such as *Anacardium* and *Spondias* had reduced R-rel variability (Figure 2), with standard deviations of just 0.02 and 0.12 respectively over 10 years (Table 1). However, even plant taxa at a distance >10 m from traps (e.g. *Dendropanax*, cf. *Gustavia*, *Hyeronima*) that were normally underrepresented could be overrepresented in a given year (Figure 2).

### Factors Governing Seasonal Variability in Pollen Composition

**Figure 3:**
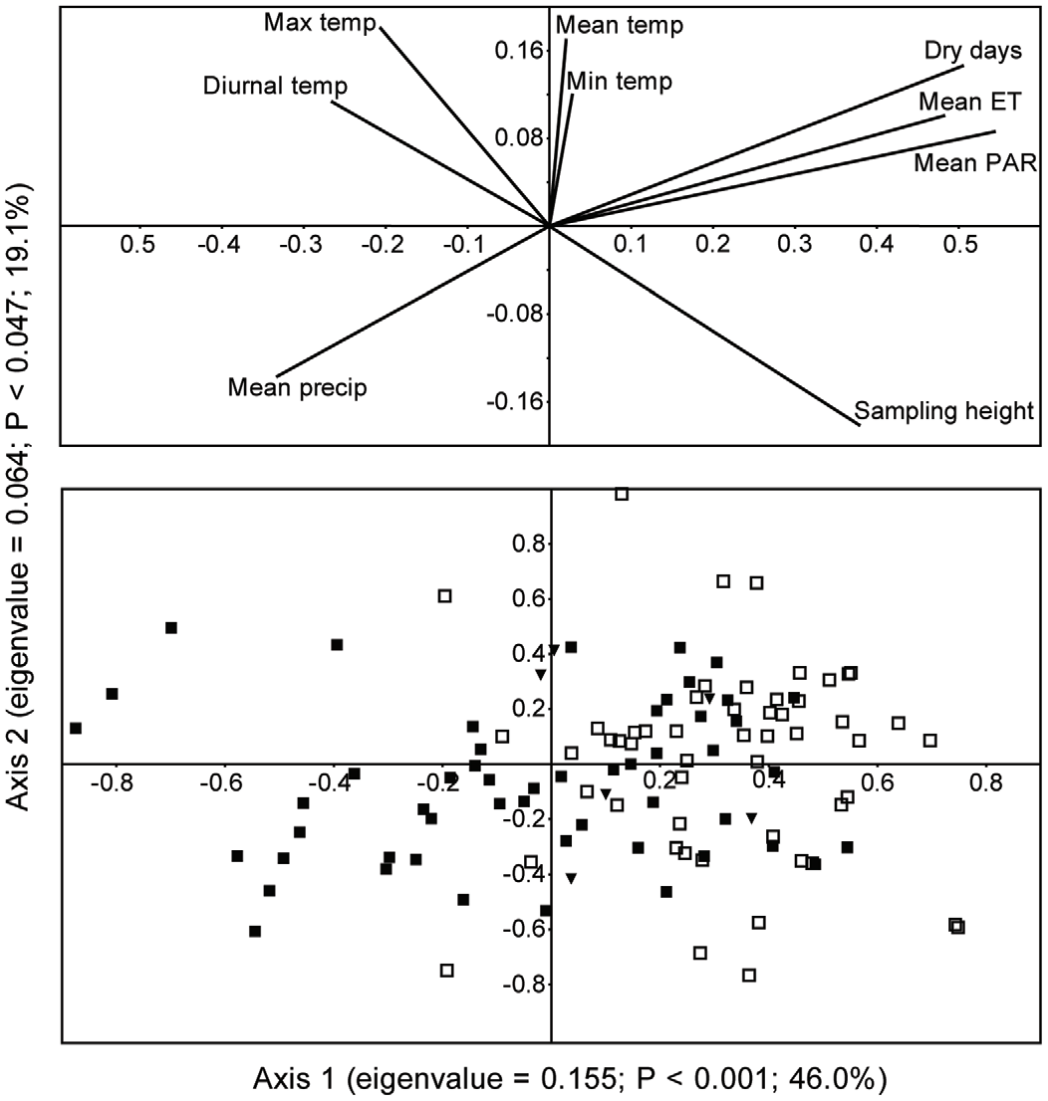
Canonical correspondence analysis of seasonal pollen influx and seasonal climatic conditions. CCA plot of the 111 seasonal pollen assemblages (restricted to the 50 most abundant pollen taxa) and nine environmental variables. The loadings of the nine environmental variables for the first two axes are plotted as vectors (top). Sample loadings for the first two axes are below, with dry season samples depicted as open boxes, wet season samples as filled boxes, and the 1998 samples as filled inverse triangles. Dry and wet season samples ordinate to the right and left, respectively.

**Figure 4:**
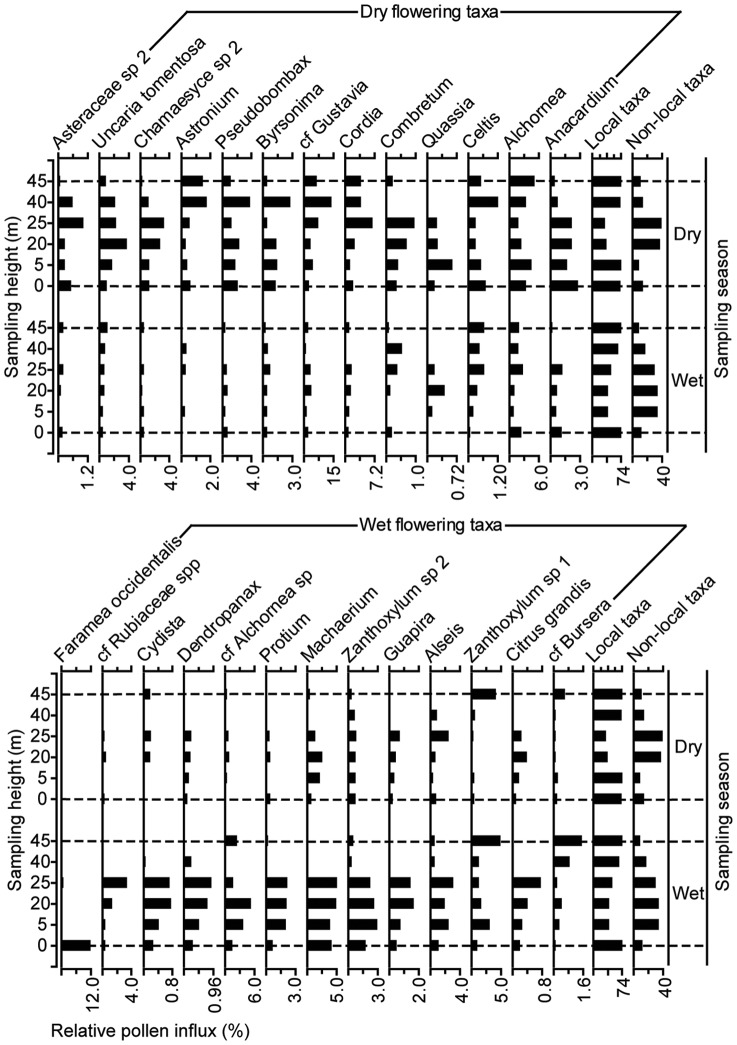
Seasonal pollen influx. Pollen diagram illustrating the compositional variability of the seasonal pollen influx, by sampling height. Pollen taxa exhibiting the most intra-annual variability in abundance over the 10-year pollen rain are graphed as their relative percentage of the total pollen influx for each season from 1996–2005. Influx data from the 1998 pollen samples are excluded because the pollen traps were not collected seasonally.

Unsurprisingly, most of the taxonomic differences in the pollen sample composition differed between wet and dry seasons. In our seasonal CCA of the 50 most abundant pollen taxa, half of the variation in the composition corresponded with seasonal environmental variables (eigenvalue = 0.155; 46.0% variation explained; *P*<0.001) (Figure 3). The relative influxes (% pollen influx) for taxa that corresponded most strongly with seasonal conditions over the nine seasonally sampled years are diagrammed in Figure 4; these are the pollen taxa with the most positive (correspondence with dry season conditions) and negative (correspondence with wet season conditions) CCA scores. Differences in the sampling methodology for the 1998 sampling period is reflected in the CCA results; all 1998 samples ordinated close to the y-axis.

The environmental variables that most accounted for differences in dry seasonal pollen influx were mean PAR, proportion of dry days and mean evapotranspiration. All these are climate measures that predictably co-vary with the reduced cloud cover and precipitation of the dry season. During the wet season, pollen influx corresponded most strongly with increases in mean precipitation.

The second CCA axis (eigenvalue = 0.064; 19.1% variation explained; *P* = 0.047) described a smaller percentage of the compositional variation in pollen influx and is characterized most strongly by sampling height. This is consistent with the observations highlighted in the previous section of the relative influx of local and non-local pollen taxa at different canopy heights. The pollen taxa that co-varied most strongly with understory sampling heights were primarily sub-canopy taxa or taxa recorded within 10 m of Lutz (*Acalypha*, *Anacardium*, *Eugenia coloradoensis*, *Faramea occidentalis*) with the exception of *Trema*; a tree taxon not recorded within 50 m of Lutz. The pollen taxa that co-varied most with increased sampling height were characterized by non-local components of the pollen rain (e.g. cf. *Alchornea* (a pollen type consistent with *Alchornea*, but distinct from *Alchornea costaricensis* and *Alchornea latifolia*), cf. *Bursera simaruba var.*, cf. Rubiaceae, *Anthurium* sp.1), with the exception of cf. *Gustavia*; an abundant sub-canopy tree taxon on BCI.

### Factors Governing Annual Variability in Pollen Composition

**Figure 5:**
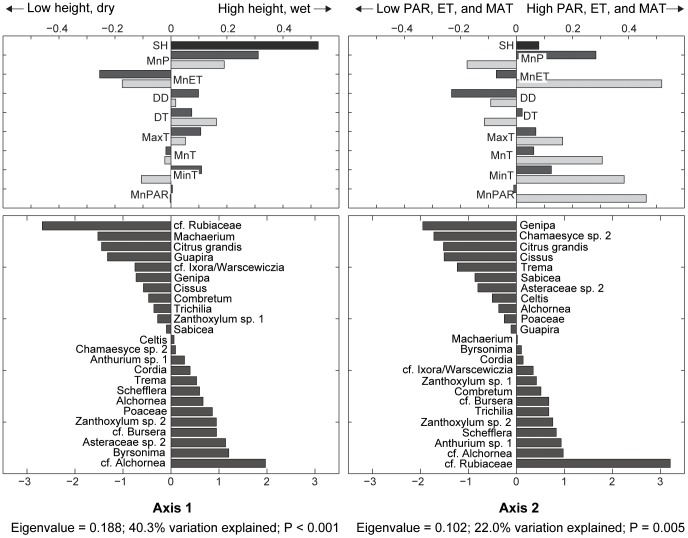
Canonical correspondence analysis of annual non-local pollen influx and current and previous years’ climatic conditions. Environmental and pollen taxon loadings for the first two ordination axes from the CCA of annual non-local pollen influx. Loadings for axis one (top left, bottom left) and loadings for axis two (top right, bottom right). Environmental loadings (top left, top right) and taxon loadings (bottom left, bottom right) are displayed as horizontal bars; the longer the bar, the stronger the correspondence of the variables for each axis. Environmental variables are denoted by the following abbreviations: SH (sampling height), MnP (mean precipitation), MnET (mean evapotranspiration), DD (proportion dry days), DT (diurnal temperature), MaxT (max temperature), MnT (mean daily temperature), MinT (minimum temperature), and MnPAR (mean photosynthetically active radiation). Light bars represent the current years’ climatic conditions; dark bars represent the previous years’ conditions. The first axis is characterized most strongly by variability in sampling height and the previous year’s precipitation. The second axis is characterized by covariance with measures of the current sampling year’s mean ET, PAR and temperature variables. CCA loadings in Table S5.

Our canonical correspondence analysis of annual pollen influx (where we summed and averaged both dry and wet season influxes by the total number of collecting days) highlighted the effect of year-to-year variation in the composition of our pollen assemblages. The CCA results for the 24 most abundant non-local taxa (taxa not within 50 m of the tower) indicated that annual variation in pollen composition co-varied most strongly with differences in sampling height and the previous year’s precipitation (eigenvalue = 0.188; 40.3% variation explained; *P*<0.001) (Figure 5, left; Table S5). Second order variation in yearly pollen composition co-varied with the current year’s evapotranspiration rates, PAR, and temperature (eigenvalue = 0.102; 22.0% variation explained; *P* = 0.005) (Figure 5, right; Table S5).

The strength of this first ordination axis was reduced (Table S6; eigenvalue = 0.106; 34.3% variation explained; *P* = 0.01) when the CCA was run with both local (26 of the 50 most abundant) and non-local pollen taxa (24 of 50 most abundant). However, for both CCAs, the first axis was dominated by differences in sampling height and mean precipitation, and the second axis of variation by evapotranspiration, PAR, and temperature (Tables S3 and Table S4). The pollen taxa that co-varied most strongly with annual differences in mean precipitation and sampling height in each analysis were cf. *Alchornea* and cf. Rubiaceae.

### Sampling Length and Pollen Influx Composition

**Figure 6:**
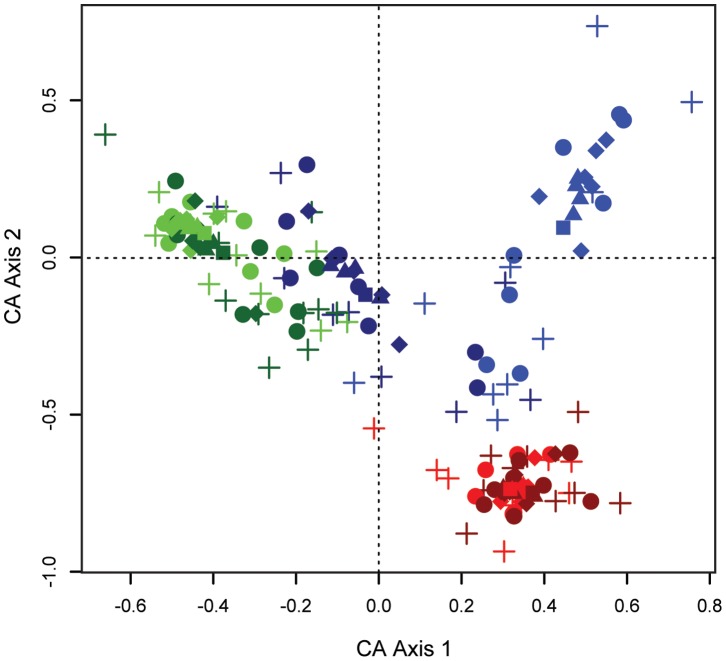
Correspondence analysis of time-averaged pollen influx by sampling height. Results of the CA for pollen samples aggregated over one, three, five, seven, and ten years, for each sampling height. Sampling heights are color-coded as follows: light blue (0 m), dark blue (5 m), light green (20 m), dark green (25 m), light red (40 m), and dark red (42/45 m). Sampling length is represented as crosses (1 year), circles (3 years), diamonds (5 years), triangles (7 years), and squares (10 years). The eigenvalues of axes one and two were 0.134 and 0.086 respectively. The largest sample-to-sample variation occurs at shorter sampling lengths and lower sampling heights.

Using CA, we compared the composition of aggregated samples spanning one, three, five, seven, and ten years. As with our CCA results, there were compositional differences in pollen influx at each sampling height (Figure 6). While the strength of the ordination axes was weak (with eigenvalues for axes one and two 0.134 and 0.086 respectively), taxonomic differences are apparent among the ground-level, mid canopy, and emergent sampling heights. Emergent 40 m (light red) and 45 m (dark red) pollen assemblages formed a tight cluster in the CA ordination, as did 20 m (light green) and 25 m (dark green) canopy pollen assemblages.

The most variation was in the samples from 0 m (light blue) and 5 m (dark blue), with the two sampling heights clustering separately. The 5 m samples were compositionally more similar to the midcanopy samples from 20 and 25 m than the 0 m ground-level samples. The composition of the 0 m samples demonstrated the most variation, with one-year and three-year samples scattering broadly along the second ordination axis. The results allowed us to heuristically determine the sampling time needed for composition to converge on the 10-year average – seven years (Figure 6).

## Discussion

Our results demonstrate the degree to which pollen output in a tropical moist forest can vary on an annual basis. A large portion of this variation, outside the expected effect of season and spatial location within the canopy, most likely correlates with small-scale, short-term variation in annual climate. Our results also illustrate the degree to which pollen-climate transfer functions based on short-term pollen rain records (<7 years) can be biased by the stochastic phenological behavior of local individuals. The high degree of variability in annual measurements of R-rel and p/v documented by our study demonstrate that paleoenvironmental interpretations based on modern tropical palynological analyses need to account for the range of natural, short-term variability in pollen productivity. Failure to do so will likely exaggerate estimates of compositional turnover over time.

In addition, our results show that analyses of modern pollen rain can detect both intra-annual and interannual variation in pollen outputs. Aerial pollen samples, perhaps taken at weekly or monthly time intervals, offer a potential means of remotely tracking tropical flowering patterns. Both past and present environmental conditions influence the variability in pollen productivity, and long-term sampling of the modern pollen rain may be one means of tracking these delayed phenological responses.

### Stratification in Pollen Influx

The placement of pollen traps at different sampling heights allowed us to assess the stratification of pollen influx within the forest canopy. Comparisons of the pollen rain at each sampling height, therefore, allowed us to test the degree to which understory pollen traps may fail to capture the full range of pollen components in analyses of modern Neotropical pollen rains. The results show that while there are differences in the pollen rain composition at different heights in the forest canopy, understory 0 and 5 m pollen traps appear to capture all the main pollen rain components stratified within the forest canopy.

Increased representation of non-local pollen taxa in upper-canopy traps (20, 25 m) suggests a greater degree of pollen movement from outside sources in the forest canopy relative to the forest understory (Figure 1). Notably, influx of long-distance, wind-dispersed pollen grains such as saccate grains (0.04% of the 10-year pollen influx) and *Alnus* (0.009% of the 10-year pollen influx) was not common at any of the sampling heights, which indicates a negligible contribution of distant montane plant taxa to modern lowland pollen assemblages.

Ground pollen samples collected within a closed canopy are thought to represent a small radius of surrounding vegetation [12]. We expected to find more non-local and long-distance dispersed taxa at higher sampling heights than our ground samples. While pollen traps at 20 and 25 m generally contained more pollen taxa and a greater relative percentage of non-local pollen influx than understory sampling heights (Figure 1), the most common non-local taxa (*Schefflera, Machaerium, Cordia,* and *Sabicea*) in canopy traps were recorded in abundance in the understory pollen traps as well – an unsurprising result considering the presence of these species elsewhere on BCI. The few non-local pollen taxa absent from the counted 0 and 5 m samples were minor components of the total pollen influx to each sampling height. Traditional ground pollen traps, therefore, capture many of the major pollen rain components, even in diverse tropical forests. When raised a few meters (e.g. 5 m) their composition additionally converges on that of canopy samples.

### Local Bias in Short-Term Pollen Counts

R-rel provides an index for identifying plant taxa that are disproportionately represented in a pollen sample relative to the standing basal area of a forest community [33], [39], with R-rel values >1 indicating plant taxa that are overrepresented and R-rel values <1 plant taxa that are underrepresented. Our results agree with previous studies of Neotropical pollen rain which demonstrated that the observed spatial variability in pollen influx to modern traps is likely influenced by proximity to different reproductive taxa [15], [19]. In this regard, our results demonstrate that modern pollen sampling protocols that fail to account for distance to nearest reproductive individual can easily over- or underestimate the long-term R-rel value of individual plant taxa in the Neotropical pollen rain.

Our annual R-rel calculations (Figure 2) show that interannual variability in R-rel is greatly exaggerated for some plant taxa located near the pollen traps, like *Faramea occidentalis* and *Eugenia coloradoensis*. Taxa that are normally underrepresented in pollen rain can easily be overrepresented when reproductive individuals are adjacent to pollen traps. It is likely that our calculated R-rel for *Faramea occidentalis* is higher previous measurements from BCI (R-rel = 0.3) [16] because of this close spatial proximity. R-rel values derived from averaging influx to spatially arranged traps are likely more reflective of the larger forest community. Our reliance on a single sampling location likely accounts for the several of the differences of our R-rel values from the published literature [7], [16], [19].

Plant taxa at a distance beyond 5 m are more consistently represented (e.g. R-rel for *Virola* ranged between 0.14 and 0.31). Nevertheless, even plant taxa that were normally underrepresented in pollen assemblages (e.g. cf. *Alseis*, *Hyeronima*) could be overrepresented in a given year. Furthermore, major basal area components (e.g. *Anacardium, Pseudobombax*) can be underrepresented – a surprising result considering that for dry environments, *Anacardium* is a major pollen component [9] and *Pseudobombax* is a strong indicator pollen type of tropical moist forests like BCI [14].

Using p/v to measure over- and underrepresentation reduced the apparent overrepresentation of some taxa (e.g. *Faramea*, Arecaceae) and increased pollen representation in other taxa (e.g. *Eugenia*, *Pseudobombax*, *Spondias*, *Virola*) (Table 1). While R-rel and p/v values tended to yield different values of underrepresentation or overrepresentation, the annual calculations of these measures for each taxa both demonstrate that on a year-to-year basis, pollen-vegetation relationships can fluctuate wildly. Together, these data illustrate that developing pollen-vegetation or pollen-climate transfer functions based on short-term pollen rain sampling will lead to results that are poor characterizations of local vegetation components.

### Phenological Behavior and Seasonal Pollen Composition

The pollen rain samples captured the seasonality of pollen production (Figures 3, 4), which was the dominant pattern of compositional change within the 10-year period. The samples captured previously described phenological patterns in many taxa. For example, positive correspondence of *Trichilia* and *Faramea* pollen with wet season conditions supports previous observations that individuals of *Trichilia tuberculata* and *Faramea occidentalis* flower in the early rainy season on BCI [40], [41]. Pollen consistent with *Cordia alliodora* was recorded in high relative abundance in mid-canopy dry season pollen samples (Figure 4), which is consistent with observations from Costa Rica that individuals of the *Cordia alliodora* flower over a six-week period in the dry season [42]. *Pseudobombax* was observed to have a strong flowering correspondence with the dry season (Figure 4), which is consistent with observations that Bombacaceous species flower throughout the dry season [43]. Dry season representation of pollen consistent with *Gustavia superba*, *Astronium graveolens* and *Anacardium excelsum* also support previous observations of flowering patterns in these species [44], [45]. Together, the results are consistent with previous studies suggesting that enhanced seasonality influences flowering activity of some Neotropical species [23], [26].

In contrast, the aseasonality observed in the pollen influx of *Hyeronima alchorneoides* is consistent for a species that has previously been shown to fruit more than once a year [46]. Similar aseasonality was observed in *Paullinia* and *Hiraea* pollen types, reflecting species previously described as having several, disjointed reproductive episodes [47], [48]. Extended flowering species such as *Spondias mombin*, which principally flowers during the dry season, but can also flower into the early rainy season [44], [49] were incorrectly also identified as aseasonal because of the timing of placement and collection of the pollen traps.

Although the coarseness of our sampling intervals means that these results are not comparable to regular forest surveys that census flowering events (e.g. [31]), our results do suggest that aerial pollen censuses are capable of capturing phenological patterns for many wind and animal-pollinated groups. When flowering events are cryptic, occur high in the canopy, or in remote locations, pollen rain traps offer a potential supplement to ground and aerial surveys. With more frequent sampling, the flowering behavior of many taxa could be tracked using aerial pollen samples.

### Interannual Variability in Pollen Rain Composition

Although the dominant pattern of variation in pollen rain composition predictably co-varied with season, second order patterns of environmental covariation indicate that composition is likely influenced by interannual differences in temperature, precipitation, ET, and PAR. The results also suggest that pollen influx may not just reflect current meteorological conditions, but are also influenced by the previous year’s weather conditions, as observed in at least one other study [50]. Our results identify the role of the previous year’s precipitation and ET and the current year’s temperature and PAR (Figure 5), suggesting the interplay of past and present environmental influences on pollen outputs.

Non-local pollen taxa (originating at a distance >50 m) demonstrated stronger covariance with our measured environmental variables than pollen originating from local taxa. The stochastic nature of the local pollen influx may mask a larger regional response to climate variability, but the spatial averaging of the regional pollen signal aggregates a broader climatic response. Pollen influx from non-local taxa reflects the flowering response of many individuals, while the local pollen signal is more likely biased by the endogenous reproductive rhythms of a few nearby individuals. In this respect, the larger regional signal that can be captured by the non-local components of the pollen rain may be an advantage to the study of landscape-scale phenological patterns.

Although our sampling interval included a severe El Niño event (1997–1998), the effects of ENSO-related weather conditions on pollen output were not apparent. Instead, pollen influx was reduced in the 1999 La Niña sampling period. However, whether this reduction in pollen influx can be directly attributed to ENSO is still speculative, as there was no obvious pattern of compositional change in the pollen rain between El Niño and La Niña years. It is possible that a clearer ENSO signal could be identified in a longer analysis of the BCI pollen rain from many spatially separated ground pollen traps, rather than the single sampling location analyzed here. Planned analyses of nearly two decades of traps placed within the BCI Center for Tropical Forest Science (CTFS) plot will attempt just that. Additionally, the repeated censuses conducted of the BCI CTFS plot will allow us to explicitly account for short-term changes in the composition of the surrounding forest, which we were unable to do in this study.

Given that fossil samples represent time-averaged samples of the pollen rain, the year-to-year variability evident in our results demonstrate that care needs to be taken when using modern pollen rain samples in paleoclimate or paleovegetation reconstructions. Modern tropical forests would be better represented by sampling intervals as long as 7 years, so that samples capture the full range of variability in the pollen rain that results from either short-term climate variability or from the endogenous reproductive behavior of these species.

### Conclusions

Quantifying the effect of climate on pollen production is a broad phenological problem requiring further research. Even studies of more easily observed phenological measures (e.g. leaf production, flowering) have had difficulty isolating the environmental variables triggering phenological responses [28], [51]–[53]. The interannual variability observed within our 10-year pollen rain suggests that year-to-year variability in pollen influx cannot be predicted based solely on MAT (e.g. [12]) or MAP (e.g. [11], [13]), but are rather a result of a combination of environmental factors. We demonstrate that even small differences in temperature, precipitation, and light availability affect the taxonomic composition and relative abundance of pollen types recorded within a pollen sample at a single site in a given year. The data also identify the taxonomic components of Neotropical pollen rain that are the most variable – and therefore may be most sensitive to any climatic variability – both in the past and the future.

Tropical pollen assemblages are not static. To improve our paleoenvironmental and paleoecological reconstructions, interpretations of pollen rain assemblages may need to be revised to accommodate an expanded understanding of the variability of the long-term pollen rain. Current practices of using one to three-year pollen rain surveys may not sufficiently capture this natural variation, and transfer functions based on these short-term datasets may grossly misrepresent the relationship between compositional change and climate. A year, or two, or three is not enough, given the variable nature of tropical plant phenology. Expanded use of surface sediments in tropical pollen calibrations (e.g. [6], [7], [13]) to analyze aerial assemblages is one solution, as sediment samples represent a longer time-averaged sample.

In this regard, samples taken from the mud-water interface likely provide a more representative record of the long-term pollen rain from which to calibrate climatological and environmental variables. However, given the flexibility afforded by aerial pollen traps in sampling different Neotropical ecosystems, the importance of modern pollen trap data in paleoenvironmental interpretations should not be devalued. A recent study comparing pollen trap data and mud-water interface samples from Bolivia demonstrated that pollen assemblages from aerial traps are comparable to surface-sediment assemblages from the same forest communities, and serve an important role in understanding how taxonomic changes within fossil pollen records correspond to changing forest environments [6]. Paleoenvironmental reconstructions based on shorter term records simply need to reflect the inherent uncertainty of these samples.

Finally, modern pollen rain studies also hold potential value for fields outside of paleoecology. The extent of male investment in reproduction can be gauged by comparing pollen rain data to other long-term phonological data sets, e.g. the floral and seed traps from the 50 ha plot on BCI [31], [46]. With finer temporal sampling, long-term aerial pollen samples may even provide a cost-effective and time-efficient way of monitoring the reproductive response of many tropical plants when flowering events are cryptic, occur high in the canopy, or in remote locations. The pollen rain effectively captures the seasonal and annual variability of tropical flowering.

## Supporting Information

Figure S1 **Seasonal pollen diagrams.** Relative abundance data for the 20 most abundant taxa at each sampling height for all 19 time periods sampled.
(PDF)

Table S1 **Relative pollen influx of all named pollen taxa recorded in the 10-year pollen rain.** Pollen taxa are ordered alphabetically with total relative influx of each pollen taxon from 1996–2005.
(PDF)

Table S2 **Species recorded in the Lutz vegetation census.** Species ordered alphabetically with the total number of reproductive individuals found in the vegetation census. The fourth column indicates whether pollen types corresponding to each species were observed in the pollen rain samples.
(PDF)

Table S3 **Seasonal pollen influx.** Measurements of pollen influx for each season at each sampling height. Mean, standard deviation of the mean, and minimum and maximum influx values of the nine years with seasonal data (1996, 1997, 1999–2005) by year and height.
(PDF)

Table S4 **Lutz tower vegetation census.** Raw census data for the 50-m survey around Lutz tower. Data on total number of individuals, relative percentage of individuals, and number of individuals in 5-m increments provided in csv format.
(CSV)

Table S5 **CCA loadings for non-local annual pollen influxes.** CCA loadings for non-local pollen taxa (taxa not found in the 50-m vegetation census) and for sampling height and the eight climatic variables described in the text. “Current” refers to the concurrent sampling year’s environmental conditions and “past” refers to the previous sampling year’s conditions.
(PDF)

Table S6 **CCA loadings for annual pollen influxes for the 50 most abundant taxa.** CCA loadings for the most abundant taxa and for sampling height and the eight climatic variables described in the text. “Current” refers to the concurrent sampling year’s environmental conditions and “past” refers to the previous sampling year’s conditions.
(PDF)

Appendix S1 **Pollen processing protocols.** Step-by-step outline of aerial trap assembly, sample processing, and slide preparation.
(PDF)

Appendix S2 **Plate images of named and unknown pollen types, Part I (Supporting Plates S1–S22).** Scale bars represent 20 µm. Multiple images are provided to highlight the texture and cross-sectional shape of each grain. Images were taken using a Plan-Apochromat SF25 (63×, 1.4NA, oil immersion) lens and a Zeiss AxioCam ICc 3 digital microscope camera.
(PDF)

Appendix S3 **Plate images of named and unknown pollen types, Part II (Supporting Plates S23–S45).** Scale bars represent 20 µm. Multiple images are provided to highlight the texture and cross-sectional shape of each grain. Images were taken using a Plan-Apochromat SF25 (63×, 1.4NA, oil immersion) lens and a Zeiss AxioCam ICc 3 digital microscope camera.
(PDF)
