# Supplementary material for: Variability within the 10-Year Pollen Rain of a Seasonal Neotropical Forest and Its Implications for Paleoenvironmental and Phenological Research
Source: PLoS One. 2013 Jan 8;8(1):e53485. doi: 10.1371/journal.pone.0053485 (PMC3540050; doi:10.1371/journal.pone.0053485)
Supplement: Figure S1 — Seasonal pollen diagrams. Relative abundance data for the 20 most abundant taxa at each sampling height for all 19 time periods sampled. (PDF) [file pone.0053485.s001.pdf]

## SUPPORTING INFORMATION

**HASELHORST, MORENO AND PUNYASENA**

***Variability within the 10-year pollen rain of a seasonal Neotropical forest and its implications for paleoenvironmental and phenological research***

**Figure S1. Seasonal pollen diagrams.** Relative abundance data for the 20 most abundant taxa at each sampling height for all 19 time periods sampled.

0 meters

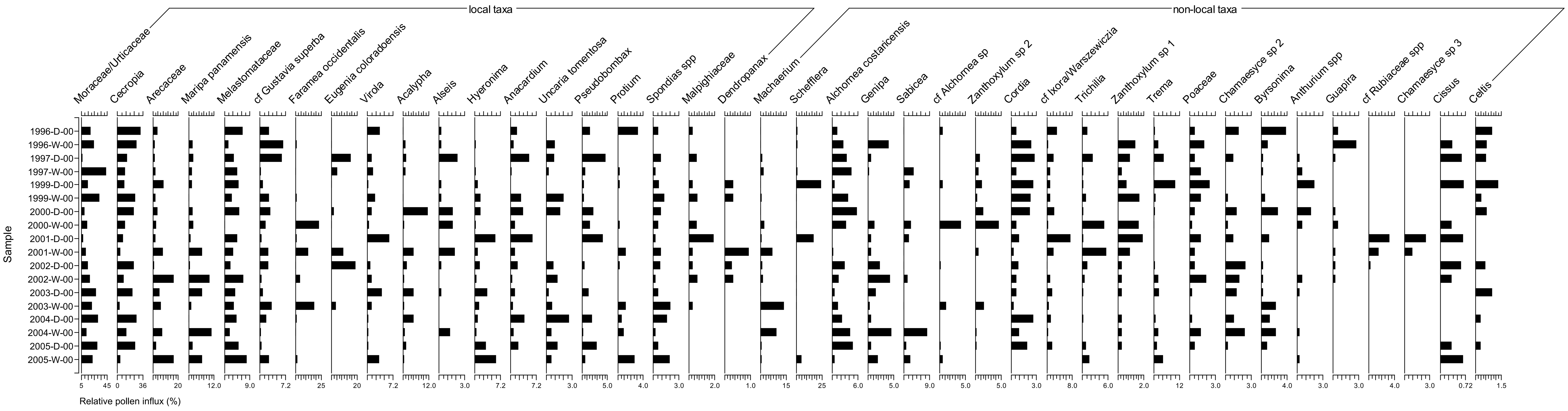

local taxa
non-local taxa
Moraceae/Urticaceae
Cecropia
Arecaceae
Maripa panamensis
Melastomataceae
cf Gustavia superba
Faramea occidentalis
Eugenia coloradoensis
Virola
Acalypha
Alseis
Hyeronima
Anacardium
Uncaria tomentosa
Pseudobombax
Protium
Spondias spp
Malpighiaceae
Dendropanax
Machaerium
Schefflera
Alchornea costaricensis
Genipa
Sabicea
cf Alchornea sp
Zanthoxylum sp 2
Cordia
cf Ixora/Warszewiczia
Trichilia
Zanthoxylum sp 1
Trema
Poaceae
Chamaesyce sp 2
Byrsonima
Anthurium spp
Guapira
cf Rubiaceae spp
Chamaesyce sp 3
Cissus
Celtis
Sample
1996-D-00
1996-W-00
1997-D-00
1997-W-00
1999-D-00
1999-W-00
2000-D-00
2000-W-00
2001-D-00
2001-W-00
2002-D-00
2002-W-00
2003-D-00
2003-W-00
2004-D-00
2004-W-00
2005-D-00
2005-W-00
Relative pollen influx (%)

5 meters

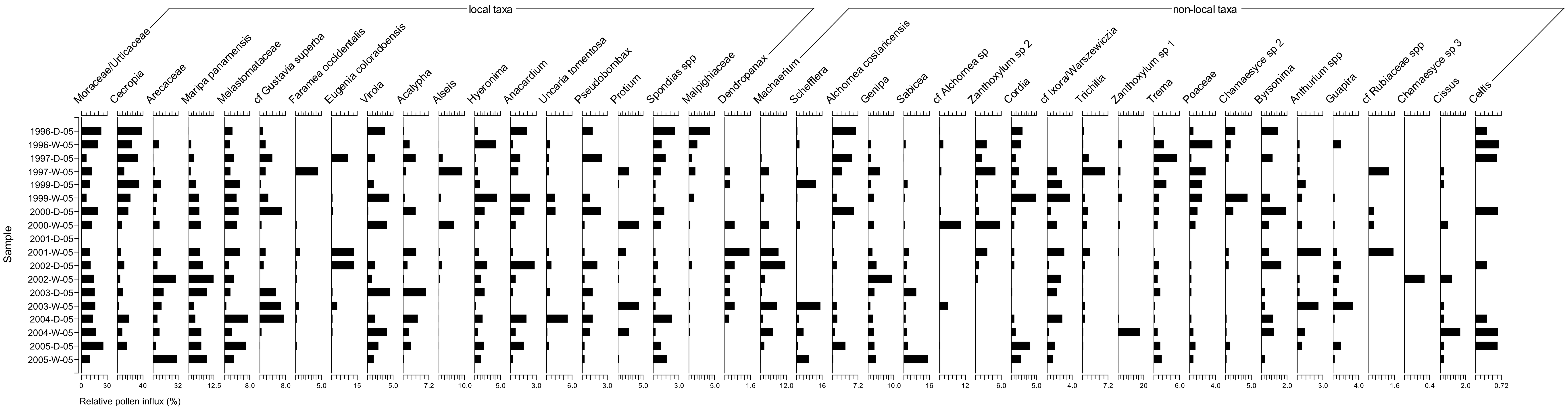
local taxa
non-local taxa
Moraceae/Urticaceae
Cecropia
Arecaceae
Mariipa panamensis
Melastomataceae
cf Gustavia superba
Faramea occidentalis
Eugenia coloradoensis
Virola
Acalypha
Alseis
Hyeronima
Anacardium
Uncaria tomentosa
Pseudobombax
Protium
Spondias spp
Malpighiaceae
Dendropanax
Machaerium
Schefflera
Alchornea costaricensis
Genipa
Sabicea
cf Alchornea sp
Zanthoxylum sp 2
Cordia
cf Ixora/Warszewiczia
Trichilia
Zanthoxylum sp 1
Trema
Poaceae
Chamaesyce sp 2
Byrsonima
Anthurium spp
Guapira
cf Rubiaceae spp
Chamaesyce sp 3
Cissus
Celtis
Sample
1996-D-05
1996-W-05
1997-D-05
1997-W-05
1999-D-05
1999-W-05
2000-D-05
2000-W-05
2001-D-05
2001-W-05
2002-D-05
2002-W-05
2003-D-05
2003-W-05
2004-D-05
2004-W-05
2005-D-05
2005-W-05
0 30 40 32 12.5 8.0 8.0 5.0 15 5.0 7.2 10.0 5.0 3.0 6.0 3.0 5.0 3.0 5.0 1.6 12.0 16 7.2 10.0 16 12 6.0 5.0 4.0 7.2 20 6.0 4.0 5.0 2.0 3.0 4.0 1.6 0.4 2.0 0.72
Relative pollen influx (%)

20 meters

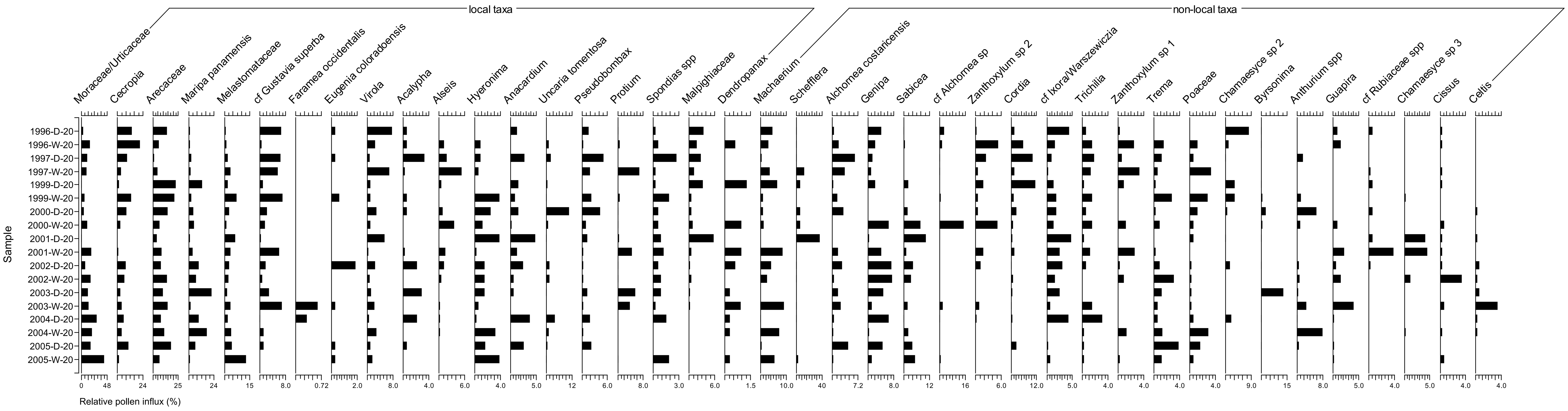
local taxa
non-local taxa
Moraceae/Urticaceae
Cecropia
Arecaceae
Maripa panamensis
Melastomataceae
cf Gustavia superba
Faramea occidentalis
Eugenia coloradoensis
Virola
Acalypha
Alseis
Hyeronima
Anacardium
Uncaria tomentosa
Pseudobombax
Protium
Spondias spp
Malpighiaceae
Dendropanax
Machaerium
Schefflera
Alchornea costaricensis
Genipa
Sabicea
cf Alchornea sp
Zanthoxylum sp 2
Cordia
cf Ixora/Warszewiczia
Trichilia
Zanthoxylum sp 1
Trema
Poaceae
Chamaesyce sp 2
Byrsonima
Anthurium spp
Guapira
cf Rubiaceae spp
Chamaesyce sp 3
Cissus
Celtis
Sample
1996-D-20
1996-W-20
1997-D-20
1997-W-20
1999-D-20
1999-W-20
2000-D-20
2000-W-20
2001-D-20
2001-W-20
2002-D-20
2002-W-20
2003-D-20
2003-W-20
2004-D-20
2004-W-20
2005-D-20
2005-W-20
Relative pollen influx (%)

25 meters

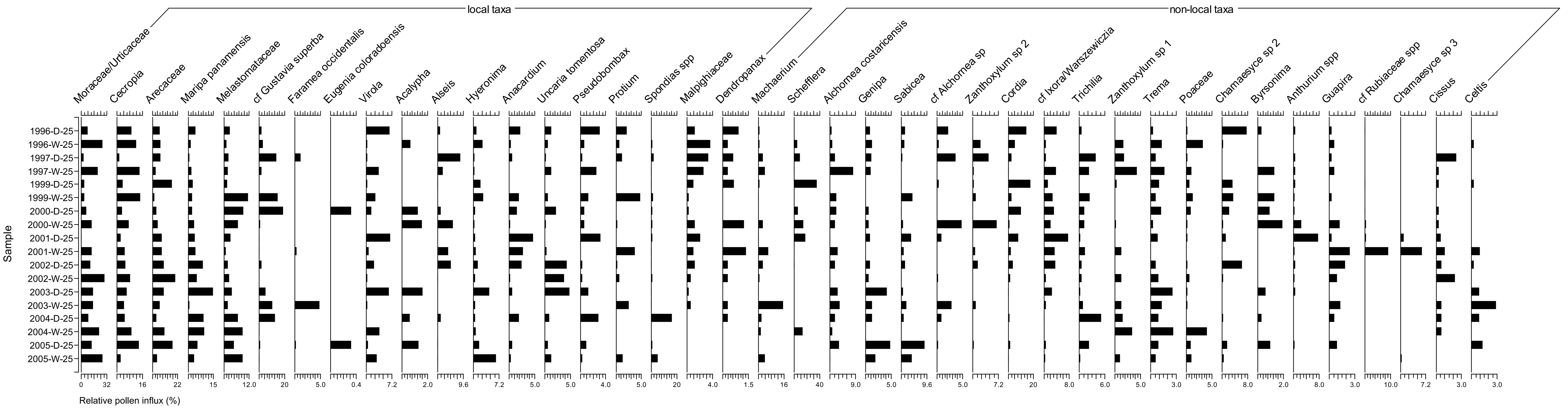

local taxa
non-local taxa
Moraceae/Urticaceae
Cecropia
Arecaceae
Maripa panamensis
Melastomataceae
cf Gustavia superba
Faramea occidentalis
Eugenia coloradoensis
Virola
Acalypha
Alseis
Hyeronima
Anacardium
Uncaria tomentosa
Pseudobombax
Protium
Spondias spp
Malpighiaceae
Dendropanax
Machaerium
Schefflera
Alchornea costaricensis
Genipa
Sabicea
cf Alchornea sp
Zanthoxylum sp 2
Cordia
cf Ixora/Warszewiczia
Trichilia
Zanthoxylum sp 1
Trema
Poaceae
Chamaesyce sp 2
Byrsonima
Anthurium spp
Guapira
cf Rubiaceae spp
Chamaesyce sp 3
Cissus
Celtis
Sample
1996-D-25
1996-W-25
1997-D-25
1997-W-25
1999-D-25
1999-W-25
2000-D-25
2000-W-25
2001-D-25
2001-W-25
2002-D-25
2002-W-25
2003-D-25
2003-W-25
2004-D-25
2004-W-25
2005-D-25
2005-W-25
0
32
16
22
15
12.0
20
5.0
0.4
7.2
2.0
9.6
7.2
5.0
5.0
4.0
5.0
20
4.0
1.5
16
40
9.0
5.0
9.6
5.0
7.2
20
8.0
6.0
5.0
3.0
5.0
8.0
2.0
8.0
3.0
10.0
7.2
3.0
3.0
Relative pollen influx (%)

40 meters

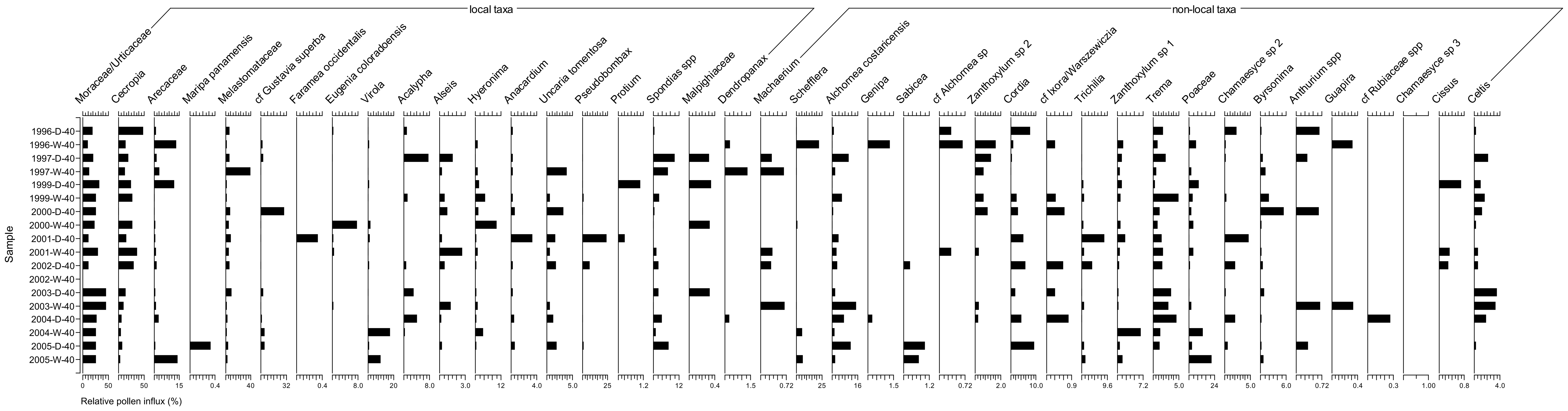
local taxa
non-local taxa
Moraceae/Urticaceae
Cecropia
Arecaceae
Maripa panamensis
Melastomataceae
cf Gustavia superba
Faramea occidentalis
Eugenia coloradoensis
Virola
Acalypha
Alseis
Hyeronima
Anacardium
Uncaria tomentosa
Pseudobombax
Protium
Spondias spp
Malpighiaceae
Dendropanax
Machaerium
Schefflera
Alchornea costaricensis
Genipa
Sabicea
cf Alchornea sp
Zanthoxylum sp 2
Cordia
cf Ixora/Warszewiczia
Trichilia
Zanthoxylum sp 1
Trema
Poaceae
Chamaesyce sp 2
Byrsonima
Anthurium spp
Guapira
cf Rubiaceae spp
Chamaesyce sp 3
Cissus
Celtis
Sample
1996-D-40
1996-W-40
1997-D-40
1997-W-40
1999-D-40
1999-W-40
2000-D-40
2000-W-40
2001-D-40
2001-W-40
2002-D-40
2002-W-40
2003-D-40
2003-W-40
2004-D-40
2004-W-40
2005-D-40
2005-W-40
0 50 50 15 0.4 40 32 0.4 8.0 20 8.0 3.0 12 4.0 5.0 25 1.2 12 0.4 1.5 0.72 25 16 1.5 1.2 0.72 2.0 10.0 0.9 9.6 7.2 5.0 24 5.0 6.0 0.72 0.4 0.3 1.00 0.8 4.0
Relative pollen influx (%)

45 meters

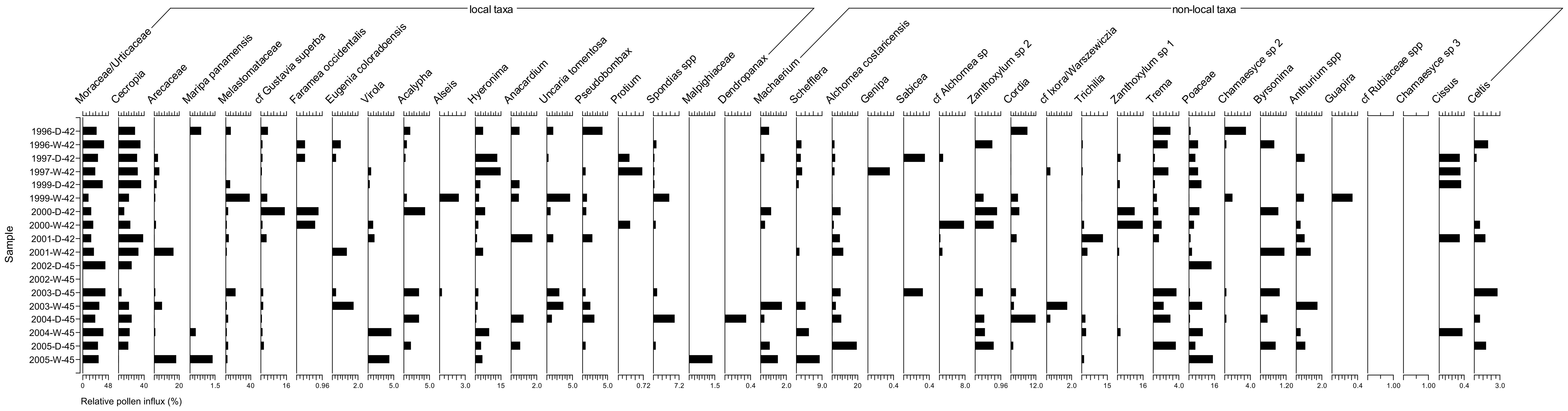
local taxa
non-local taxa
Moraceae/Urticaceae
Cecropia
Arecaceae
Maripa panamensis
Melastomataceae
cf Gustavia superba
Faramea occidentalis
Eugenia coloradoensis
Virola
Acalypha
Alseis
Hyeronima
Anacardium
Uncaria tomentosa
Pseudobombax
Protium
Spondias spp
Malpighiaceae
Dendropanax
Machaerium
Schefflera
Alchornea costaricensis
Genipa
Sabicea
cf Alchornea sp
Zanthoxylum sp 2
Cordia
cf Ixora/Warszewiczia
Trichilia
Zanthoxylum sp 1
Trema
Poaceae
Chamaesyce sp 2
Byrsonima
Anthurium spp
Guapira
cf Rubiaceae spp
Chamaesyce sp 3
Cissus
Celtis
Sample
1996-D-42
1996-W-42
1997-D-42
1997-W-42
1999-D-42
1999-W-42
2000-D-42
2000-W-42
2001-D-42
2001-W-42
2002-D-45
2002-W-45
2003-D-45
2003-W-45
2004-D-45
2004-W-45
2005-D-45
2005-W-45
0 48 40 20 1.5 40 16 0.96 2.0 5.0 5.0 3.0 15 2.0 5.0 5.0 0.72 7.2 1.5 0.4 2.0 9.0 20 0.4 0.4 8.0 0.96 12.0 2.0 15 16 4.0 16 4.0 1.20 2.0 0.4 1.00 1.00 0.4 3.0
Relative pollen influx (%)
