# Supplementary material for: Variability within the 10-Year Pollen Rain of a Seasonal Neotropical Forest and Its Implications for Paleoenvironmental and Phenological Research
Source: PLoS One. 2013 Jan 8;8(1):e53485. doi: 10.1371/journal.pone.0053485 (PMC3540050; doi:10.1371/journal.pone.0053485)
Supplement: Table S1 — Relative pollen influx of all named pollen taxa recorded in the 10-year pollen rain. Pollen taxa are ordered alphabetically with total relative influx of each pollen taxon from 1996–2005. (PDF) [file pone.0053485.s002.pdf]

SUPPORTING INFORMATION

HASELHORST, MORENO AND PUNYASENA

Variability within the 10-year pollen rain of a seasonal Neotropical forest and its implications for paleoenvironmental and phenological research

Table S1. Relative pollen influx of all named pollen taxa recorded in the 10-year pollen rain. Pollen taxa are ordered alphabetically with total relative influx of each pollen taxon from 1996-2005.

Pollen Taxa	Family	Relative % Total Pollen Influx
Acalypha	Euphorbiaceae	1.49
Alchornea costaricensis	Euphorbiaceae	2.31
Alchornea latifolia	Euphorbiaceae	0.03
cf. *Alchornea*	Euphorbiaceae	1.81
Alnus	Betulaceae	0.01
Alseis	Rubiaceae	1.47
Anacardium	Anacardiaceae	1.22
Anthurium sp.1	Araceae	0.73
Anthurium sp.2	Araceae	0.01
cf. *Apeiba* sp.	Tiliaceae	0.04
Arecaceae	Arecaceae	7.50
Arrabidaea	Bignoniaceae	0.31
Asteraceae sp.1	Asteraceae	0.14
Asteraceae sp.2	Asteraceae	0.23
Astronium	Anacardiaceae	0.30
Bombacopsis	Malvaceae	0.16
Bursera	Burseraceae	0.14
cf. *Bursera*	Burseraceae	0.22
Byrsonima	Malpighiaceae	0.81
Casearia	Salicaceae	0.08
Cavanillesia	Malvaceae	0.05
Cecropia	Urticaceae	11.9
Cedrela	Meliaceae	0.01
Ceiba	Malvaceae	0.13
Celtis	Cannabaceae	0.35
aff. *Cespedezia*	Ochnaceae	0.03
Chamaesyce sp.1	Euphorbiaceae	0.20

Chamaesyce sp.2	Euphorbiaceae	0.86
Chamaesyce sp.3	Euphorbiaceae	0.45
Chamissoa	Amaranthaceae	0.01
Chenopodium	Amaranthaceae	0.02
Cissus	Vitaceae	0.35
Citrus grandis	Rutaceae	0.26
Citrus sp.1	Rutaceae	0.12
Combretum-type	Combretaceae	0.28
Combretum sp.2	Combretaceae	.001
Cordia	Boraginaceae	1.61
Coussarea	Rubiaceae	0.08
Croton	Euphorbiaceae	0.03
Cryosophila	Arecaceae	0.15
Cydista	Bignoniaceae	0.29
Dalechampia	Euphorbiaceae	0.01
Dendropanax	Araliaceae	0.36
Schefflera	Araliaceae	2.55
Erythrina costaricensis	Fabaceae - Papilionoideae	0.03
Eugenia coloradoensis	Myrtaceae	2.13
Eugenia sp.	Myrtaceae	0.10
unknown Euphorbiaceae sp.1	Euphorbiaceae	0.20
unknown Euphorbiaceae sp.2	Euphorbiaceae	0.07
Faramea occidentalis	Rubiaceae	2.15
Faramea sp.2	Rubiaceae	0.01
Faramea sp.3	Rubiaceae	0.01
Ficus	Moraceae	0.24
Genipa	Rubiaceae	1.97
Guapira	Nyctaginaceae	0.67
cf. *Gustavia superba*	Lecythidaceae	3.10
Hedyosmum sp.	Chloranthaceae	0.08
Hippocratea	Hippocrataceae	0.05
Hyeronima	Euphorbiaceae	1.44
cf. *Hyptis*	Lamiaceae	0.03
Ilex sp.	Aquifoliaceae	0.02
cf. *Inga*	Fabaceae - Mimosoideae	0.05
Jacaranda	Bignoniaceae	0.01
Lacmellea panamensis	Apocynaceae	0.05
cf. *Laetia procera*	Salicaceae	0.08
Machaerium	Fabaceae - Papilionoideae	2.95
Hiraea-type	Malpighiaceae	0.65
Mansoa	Bignoniaceae	.003
Maripa panamensis	Convolvulaceae	4.17
cf. *Melochia*	Sterculiaceae	0.01

Melothria	Cucurbitaceae	0.05
Mendoncia gracilis	Acanthaceae	0.01
Melastomataceae	Melastomataceae	3.86
Mimosoideae sp.2	Fabaceae - Mimosoideae	.001
Moraceae/Urticaceae	Moraceae/Urticaceae	17.6
Myrcia	Myrtaceae	0.04
Oryctanthus	Loranthaceae	.005
Paullinia	Sapindaceae	0.23
Pinus/Podocarpus	---	0.04
Piper	Piperaceae	0.19
Poaceae	Poaceae	1.03
Posoqueria	Rubiaceae	0.08
Pouteria	Sapotaceae	0.04
Protium	Burseraceae	0.98
Pseudobombax	Malvaceae	1.03
Psychotria sp.1	Rubiaceae	0.21
Psychotria sp. 2	Rubiaceae	0.03
Quararibea asterolepis	Malvaceae	.003
Quassia	Simaroubaceae	0.22
Roupala montana	Proteaceae	0.03
cf. *Rubiaceae* spp.	Rubiaceae	0.65
cf. Rutaceae spp.	Rutaceae	.004
Sabicea	Rubiaceae	1.86
Sapium	Euphorbiaceae	0.13
Serjania	Sapindaceae	0.05
Simarouba	Simaroubaceae	0.05
aff. *Sida*	Malvaceae	.003
Socratea	Arecaceae	0.15
Solanum	Solanaceae	0.32
Spondias	Anacardiaceae	0.88
Spondias sp.2	Anacardiaceae	0.10
Tabebuia	Bignoniaceae	0.07
Tocoyena pittieri	Rubiaceae	.004
Trema	Cannabaceae	1.22
Trichilia	Meliaceae	1.43
Uncaria tomentosa	Rubiaceae	1.06
Virola	Myristicaceae	1.50
cf. *Vismia*	Hypericaceae	0.13
Vochisyia	Vochysiaceae	0.09
cf. *Warscewiczia*/*Ixora*	Rubiaceae	1.57
Zanthoxylum sp.1	Rutaceae	1.24
Zanthoxylum sp.2	Rutaceae	1.63
