# Supplementary material for: Variability within the 10-Year Pollen Rain of a Seasonal Neotropical Forest and Its Implications for Paleoenvironmental and Phenological Research
Source: PLoS One. 2013 Jan 8;8(1):e53485. doi: 10.1371/journal.pone.0053485 (PMC3540050; doi:10.1371/journal.pone.0053485)
Supplement: Table S2 — Species recorded in the Lutz vegetation census. Species ordered alphabetically with the total number of reproductive individuals found in the vegetation census. The fourth column indicates whether pollen types corresponding to each species were observed in the pollen rain samples. (PDF) [file pone.0053485.s003.pdf]

SUPPORTING INFORMATION

HASELHORST, MORENO AND PUNYASENA

Variability within the 10-year pollen rain of a seasonal Neotropical forest and its implications for paleoenvironmental and phenological research

Table S2. Species recorded in the Lutz vegetation census.

Species ordered alphabetically with the total number of reproductive individuals found in the vegetation census. The fourth column indicates whether pollen types corresponding to each species were observed in the pollen rain samples.

**Species**	**Family**	**# Individuals**	**Pollen Rain (Y/N)**
Acalypha diversifolia	Euphorbiaceae	4	Y
Alibertia edulis	Rubiaceae	2	N
Allophylus psilospermus	Sapindaceae	1	N
Alseis blackiana	Rubiaceae	12	Y
Anacardium excelsum	Anacardiaceae	12	Y
Annona spraguei	Annonaceae	1	N
Arrabidaea candicans	Bignoniaceae	2	Y
Astrocaryum standleyanum	Arecaceae	3	Y
Astronium graveolens	Anacardiaceae	1	Y
Bombacopsis quinata	Malvaceae	1	Y
Brosimum alicastrum	Moraceae	3	Y
Calathea inocephala	Marantaceae	6	N
Capparis frondosa	Capparaceae	83	N
Casearia aculeata	Salicaceae	5	Y
Cassipourea elliptica	Rhizophoraceae	1	N
Cecropia insignis	Urticaceae	2	Y
Cestrum megalophyllum	Solanaceae	1	N
Chrysochlamys eclipes	Clusiaceae	2	N
Chrysophyllum argenteum	Sapotaceae	2	N
Clitoria javitensis	Fabaceae-Papilionoideae	2	N
Cupania sylvatica	Sapindaceae	1	N
Cydista aequinoctialis	Bignoniaceae	1	Y
Dendropanax arboreus	Araliaceae	1	Y
Desmopsis panamensis	Annonaceae	15	N
Dieffenbachia sp.	Araceae	4	N
Dipteryx oleifera	Fabaceae-Papilionoideae	1	N

Doliocarpus dentatus	Dilleniaceae	1	N
Doliocarpus olivaceus	Dilleniaceae	10	N
Erythrina costaricensis	Fabaceae-Papilionoideae	3	Y
Eugenia coloradoensis	Myrtaceae	1	Y
Eugenia oerstediana	Myrtaceae	1	Y
Faramea occidentalis	Rubiaceae	43	Y
Ficus insipida	Moraceae	3	Y
Garcinia intermedia	Clusiaceae	2	N
Garcinia madruno	Clusiaceae	2	N
Guettarda foliacea	Rubiaceae	1	N
Gustavia superba	Lecythidaceae	26	Y
Hamelia axillaris	Rubiaceae	1	N
Hasseltia floribunda	Salicaceae	2	N
Heisteria acuminata	Olacaceae	3	N
Heisteria concinna	Olacaceae	2	N
Herrania purpurea	Malvaceae	9	N
Heteropterys laurifolia	Malpighiaceae	1	N
Hyeronima alchorneoides	Euphorbiaceae	4	Y
Hiraea grandifolia	Malpighiaceae	35	Y
Hirtella triandra	Chrysobalanaceae	61	N
Hybanthus prunifolius	Violaceae	8	N
Inga goldmanii	Fabaceae-Mimosoideae	1	Y
Inga nobilis	Fabaceae-Mimosoideae	4	Y
Lacistema aggregatum	Lacistemataceae	6	N
Lacmellea panamensis	Apocynaceae	1	Y
Licania platypus	Chrysobalanaceae	1	N
Macrocnemum roseum	Rubiaceae	31	N
Maripa panamensis	Convolvulaceae	1	Y
Mendoncia gracilis	Acanthaceae	1	Y
Mendoncia litoralis	Acanthaceae	1	Y
Mikania leiostachya	Asteraceae	9	N
Mouriri myrtilloides	Melastomataceae	21	Y
Myriocarpa longipes	Urticaceae	10	N
Oenocarpus mapora	Arecaceae	1	Y
Omphalea diandra	Euphorbiaceae	7	N
Ouratea lucens	Ochnaceae	1	N
Paullinia baileyi	Sapindaceae	1	Y
Paullinia fibrigera	Sapindaceae	1	Y
Pentagonia macrophylla	Rubiaceae	8	N
Petrea volubilis	Verbenaceae	9	N
Phryganocydia corymbosa	Bignoniaceae	5	N
Picramnia latifolia	Picramniaceae	1	N
Piper aequale	Piperaceae	3	Y
Piper arboreum	Piperaceae	6	Y
Piper auritum	Piperaceae	1	Y
Piper colonense	Piperaceae	3	Y

Piper cordulatum	Piperaceae	6	Y
Piper grande	Piperaceae	361	Y
Piper perlasense	Piperaceae	7	Y
Piper reticulatum	Piperaceae	8	Y
Platypodium elegans	Fabaceae-Papilionoideae	1	N
Pleiostachya pruinosa	Marantaceae	1	N
Posoqueria latifolia	Rubiaceae	6	Y
Poulsenia armata	Moraceae	4	Y
Pouteria reticulata	Sapotaceae	1	Y
Prionostemma aspera	Celastraceae	1	N
Protium costaricense	Burseraceae	1	Y
Protium panamense	Burseraceae	2	Y
Protium tenuifolium	Burseraceae	12	Y
Pseudobombax septenatum	Malvaceae	1	Y
Pseudolmedia spuria	Moraceae	1	N
Psychotria horizontalis	Rubiaceae	78	Y
Psychotria limonensis	Rubiaceae	54	Y
Psychotria marginata	Rubiaceae	3	Y
Psychotria suerrensis	Rubiaceae	5	Y
Quararibea asterolepis	Malvaceae	4	Y
Quassia amara	Simaroubaceae	8	Y
Randia armata	Rubiaceae	2	N
Siparuna pauciflora	Siparunaceae	2	N
Socratea exorrhiza	Arecaceae	3	Y
Solanum aturense	Solanaceae	1	Y
Sorocea affinis	Moraceae	26	Y
Spondias mombin	Anacardiaceae	1	Y
Spondias radlkoferi	Anacardiaceae	1	Y
Stylogyne turbacensis	Primulaceae	1	N
Swartzia simplex var.grandiflora	Fabaceae-Papilionoideae	23	N
Swartzia simplex var.ochnacea	Fabaceae-Papilionoideae	1	N
Synechanthus warscewiczianus	Arecaceae	4	N
Terminalia oblonga	Combretaceae	1	N
Tetragastris panamensis	Burseraceae	1	N
Tontelea richardii	Celastraceae	6	N
Trophis racemosa	Moraceae	3	N
Uncaria tomentosa	Rubiaceae	12	Y
Unonopsis pittieri	Annonaceae	4	N
Virola sebifera	Myristicaceae	3	Y
Virola surinamensis	Myristicaceae	5	Y
Xylopia macrantha	Annonaceae	4	N
