# Supplementary material for: Variability within the 10-Year Pollen Rain of a Seasonal Neotropical Forest and Its Implications for Paleoenvironmental and Phenological Research
Source: PLoS One. 2013 Jan 8;8(1):e53485. doi: 10.1371/journal.pone.0053485 (PMC3540050; doi:10.1371/journal.pone.0053485)
Supplement: Table S3 — Seasonal pollen influx. Measurements of pollen influx for each season at each sampling height. Mean, standard deviation of the mean, and minimum and maximum influx values of the nine years with seasonal data (1996, 1997, 1999–2005) by year and height. (PDF) [file pone.0053485.s004.pdf]

SUPPORTING INFORMATION

HASELHORST, MORENO AND PUNYASENA

Variability within the 10-year pollen rain of a seasonal Neotropical forest and its implications for paleoenvironmental and phenological research

Table S3. Seasonal pollen influx. Measurements of pollen influx for each season at each sampling height. . Mean, standard deviation of the mean, and minimum and maximum influx values of the nine years with seasonal data (1996, 1997, 1999 – 2005) by year and height.

Sample	Mean influx (pollen grains/cm2/day)	Standard deviation (σ)	Min influx (pollen grains/cm2/day)	Max influx (pollen grains/cm2/day)
00 m dry	46.77	29.5	18.14	114.68
00 m wet	49.77	46.68	9.02	130.84
05 m dry	55.88	71.5	13.07	230.11
05 m wet	49.08	39.87	9.71	125.91
20 m dry	31.69	26.44	7.5	79.21
20 m wet	36.22	44.93	3.48	128.71
25 m dry	25.91	20.54	7.04	58.47
25 m wet	26.39	30.48	4.16	96.95
40 m dry	10.11	13.72	0.57	43.15
40 m wet	6.92	8.24	0.27	25.18
45 m dry	3.61	3.3	0.43	11.19
45 m wet	3.41	3.11	0.19	8.27
