# Supplementary material for: Variability within the 10-Year Pollen Rain of a Seasonal Neotropical Forest and Its Implications for Paleoenvironmental and Phenological Research
Source: PLoS One. 2013 Jan 8;8(1):e53485. doi: 10.1371/journal.pone.0053485 (PMC3540050; doi:10.1371/journal.pone.0053485)
Supplement: Table S5 — CCA loadings for non-local annual pollen influxes. CCA loadings for non-local pollen taxa (taxa not found in the 50-m vegetation census) and for sampling height and the eight climatic variables described in the text. “Current” refers to the concurrent sampling year’s environmental conditions and “past” refers to the previous sampling year’s conditions. (PDF) [file pone.0053485.s006.pdf]

SUPPORTING INFORMATION

HASELHORST, MORENO AND PUNYASENA

Variability within the 10-year pollen rain of a seasonal Neotropical forest and its implications for paleoenvironmental and phenological research

Table S5. CCA loadings for non-local annual pollen influxes. CCA loadings for non-local pollen taxa (taxa not found in the 50-m vegetation census) and for sampling height and the eight climatic variables described in the text. "Current" refers to the concurrent sampling year's environmental conditions and "past" refers to the previous sampling year's conditions.

	**CCA loadings** (non-local pollen components)	**Axis 1** (eigenvalue = 0.188; 40.3% variation explained; $P < 0.001$)	**Axis 2** (eigenvalue = 0.102; 22.0% variation explained; $P = 0.005$)	**Axis 3** (eigenvalue = 0.068; 14.7% variation explained; $P = 0.022$)
**Environmental loadings**	Sampling height	0.526	0.080	-0.237
	Mean ET past	-0.255	-0.072	0.045
	Mean ET current	-0.174	0.519	0.273
	Mean PAR past	0.005	-0.010	-0.030
	Mean PAR current	-0.003	0.464	0.072
	Mean precip. past	0.312	0.284	-0.008
	Mean precip. current	0.190	-0.176	-0.513
	Mean temp. past	-0.018	0.062	-0.228
	Mean temp. current	-0.022	0.307	0.142
	Min temp. past	0.109	0.125	-0.097
	Min temp. current	-0.106	0.385	-0.005
	Max temp. past	0.106	0.069	-0.407
	Max temp. current	0.051	0.165	0.099
	Diurnal temp past	0.074	0.020	-0.451
	Diurnal temp current	0.162	-0.114	0.138
	Dry days past	0.098	-0.232	-0.316
	Dry days current	0.017	-0.091	0.141

**Taxon loadings**	*Guapira*	-1.323	-0.111	0.564
	Alchornea	0.668	-0.368	0.648
	Anthurium sp.1	0.281	0.933	-0.039
	Asteraceae sp.2	1.139	-0.802	0.409
	cf. *Bursera*	0.948	0.673	1.139
	Byrsonima	1.204	0.105	0.578
	Celtis	0.062	-0.502	-0.520
	Cissus	-0.573	-1.505	-0.073
	Citrus grandis	-1.445	-1.519	0.232
	Combretum	-0.465	0.511	0.819
	Cordia	0.397	0.139	-0.923
	Schefflera	0.597	0.831	-2.012
	Genipa	-0.726	-1.949	0.573
	Chamaesyce sp. 2	0.093	-1.718	-0.103
	cf. *Alchornea* sp.	1.968	0.974	1.792
	cf. *Warscewiczia*	-0.748	0.349	-0.030
	cf. Rubiaceae spp.	-2.680	3.204	0.124
	Machaerium	-1.523	0.019	0.435
	Poaceae	0.862	-0.252	-0.991
	Sabicea	-0.095	-0.862	-0.044
	Trema	0.535	-1.231	-1.409
	Trichilia	-0.355	0.674	0.741
	Zanthoxylum sp.1	-0.276	0.418	-1.279
	Zanthoxylum sp.2	0.945	0.756	1.271
