# Supplementary material for: Variability within the 10-Year Pollen Rain of a Seasonal Neotropical Forest and Its Implications for Paleoenvironmental and Phenological Research
Source: PLoS One. 2013 Jan 8;8(1):e53485. doi: 10.1371/journal.pone.0053485 (PMC3540050; doi:10.1371/journal.pone.0053485)
Supplement: Table S6 — CCA loadings for annual pollen influxes for the 50 most abundant taxa. CCA loadings for the most abundant taxa and for sampling height and the eight climatic variables described in the text. “Current” refers to the concurrent sampling year’s environmental conditions and “past” refers to the previous sampling year’s conditions. (PDF) [file pone.0053485.s007.pdf]

SUPPORTING INFORMATION

HASELHORST, MORENO AND PUNYASENA

Variability within the 10-year pollen rain of a seasonal Neotropical forest and its implications for paleoenvironmental and phenological research

Table S6. CCA loadings for annual pollen influxes for the 50 most abundant taxa. CCA loadings for the most abundant taxa and for sampling height and the eight climatic variables described in the text. "Current" refers to the concurrent sampling year's environmental conditions and "past" refers to the previous sampling year's conditions.

	**CCA loadings** (50 most abundant components)	**Axis 1** (eigenvalue = 0.106; 34.3% variation explained; *P* < 0.01)	**Axis 2** (eigenvalue = 0.062; 20.1% variation explained; *P* = 0.175)	**Axis 3** (eigenvalue = 0.056; 18.2% variation explained; *P* = 0.002)
**Environmental loadings**	Sampling height	-0.609	0.060	0.323
	Mean ET past	0.252	-0.063	0.152
	Mean ET current	0.252	0.287	-0.480
	Mean PAR past	0.058	-0.105	0.063
	Mean PAR current	0.060	0.238	-0.418
	Mean precip. past	-0.326	0.258	0.059
	Mean precip. current	-0.277	-0.061	0.379
	Mean temp. past	0.032	0.006	0.059
	Mean temp. current	0.113	0.120	-0.331
	Min temp. past	-0.096	0.101	-0.166
	Min temp. current	0.150	0.206	-0.249
	Max temp. past	-0.119	0.010	0.130
	Max temp. current	0.053	0.010	-0.225
	Diurnal temp past	-0.097	-0.040	0.247
	Diurnal temp current	-0.060	-0.166	-0.085
	Dry days past	-0.087	-0.216	0.339
	Dry days current	0.050	-0.164	-0.081
**Taxon loadings**	*Acalypha*	0.280	-0.410	-2.325
	Guapira	1.549	1.360	1.113
	Alchornea	-0.771	-0.418	-0.732
	Alseis	-0.249	1.009	-0.736
	Anacardium	0.595	-0.078	-0.350
	Anthurium sp.1	-0.466	1.671	-0.439
	Arecaceae	0.550	-0.212	0.849
	Arrabidaea	-0.480	-0.454	0.673
	Asteraceae sp.2	-1.562	-0.517	-0.107

cf. Bursera	-1.345	1.626	-0.663
Byrsonima	-1.581	0.333	-0.858
Cecropia	-0.519	-0.890	-0.228
Celtis	-0.454	0.520	0.970
Cissus	0.563	-0.656	1.646
Citrus grandis	1.628	-0.091	1.972
Combretum	0.582	1.415	-0.123
Cordia	-0.845	1.027	0.962
Cydista	2.889	3.013	0.886
Schefflera	-1.037	1.322	0.293
Faramea occidentalis	1.250	-0.165	-3.915
Genipa	0.822	-1.193	1.404
Hyeronima	-0.679	0.193	0.587
Chamaesyce sp. 2	-0.313	-1.029	1.232
Dendropanax	1.786	1.989	0.038
Psychotria	2.042	1.939	2.264
Eugenia coloradoensis	2.550	-1.868	-1.391
cf. Gustavia	-1.243	1.610	-0.030
cf. Alchornea sp.	-2.406	1.141	-2.537
cf. Warscewiczia	0.940	1.119	0.283
cf. Rubiaceae spp.	3.477	5.125	0.235
Astronium	-1.366	0.438	0.676
Uncaria tomentosa	-1.368	0.132	0.567
Machaerium	2.051	0.781	0.187
Malpighiaceae	0.484	1.021	0.548
Maripa	0.581	-0.487	0.439
Melastomataceae	-0.629	0.344	0.652
Moraceae/Urticaceae	-0.019	-0.594	0.281
Paullinia	-0.898	0.838	-0.890
Poaceae	-1.407	0.282	0.741
Protium	1.513	2.386	-0.243
Pseudobombax	-0.695	0.070	-0.512
Quassia	-1.004	0.377	-1.175
Sabicea	0.047	-0.636	0.715
Solanum	-0.996	0.481	-0.192
Spondias	-0.538	0.587	1.363
Trema	-0.796	-1.447	0.651
Trichilia	0.714	0.805	-1.234
Virola	-0.112	-0.154	0.388
Zanthoxylum sp.1	0.085	1.032	0.698
Zanthoxylum sp.2	-0.964	0.778	-2.002
