# Supplementary material for: Variability within the 10-Year Pollen Rain of a Seasonal Neotropical Forest and Its Implications for Paleoenvironmental and Phenological Research
Source: PLoS One. 2013 Jan 8;8(1):e53485. doi: 10.1371/journal.pone.0053485 (PMC3540050; doi:10.1371/journal.pone.0053485)
Supplement: Appendix S1 — Pollen processing protocols. Step-by-step outline of aerial trap assembly, sample processing, and slide preparation. (PDF) [file pone.0053485.s008.pdf]

SUPPORTING INFORMATION

HASELHORST, MORENO AND PUNYASENA

Variability within the 10-year pollen rain of a seasonal Neotropical forest and its implications for paleoenvironmental and phenological research

Appendix S1. Pollen processing protocols. Step-by-step outline of aerial trap assembly, sample processing, and slide preparation.

Funnel trap assembly

The pollen traps used in this study were assembled following the methods and materials described in [32]. Assembled pollen traps consist of a plastic funnel that is lined with a filter (Whatman GF/D 7 micrometer) and inserted with rayon fiber to capture and retain the sampled pollen rain. The funnel portion of the trap is then affixed to a plastic bottle from which accumulated precipitation can drain, thereby preventing the pollen from being flooded out of the trap funnel and allowing for extended sampling periods. Pollen traps can be quickly assembled using a plastic funnel, plastic bottle, Whatman GF/D filter, rayon, mesh window, silicone (glue), needles, nylon, and scissors (Figure S1.1a). The outline of the trap assemblage is as follows:

1. Glue a small square of screen mesh to the base of the inner funnel to prevent the loss of the Whatman filter and rayon fiber (Figure S1.1b,c).
2. Over the top of the mesh netting, glue a Whatman GF/D 7 micrometer filter to the funnel so that the filter is firmly pressed to the sides of the funnel (Figure S1.1d,e).
3. Add a small ball of rayon fiber to the funnel (Figure S1.1f). The rayon fiber serves as the pollen trapping medium.
4. Cover the top of the funnel with a mesh window screen (Figure S1.1g). The mesh prevents insects and leaves from entering the trap funnel.
5. Fix the funnel to the plastic bottle (Figure S1.1h). To do so, poke two small water drainage holes towards the top of the plastic bottle, and then tie the funnel to the bottle using a needle and nylon.
6. If sampling aerially at different heights, use a PVC tube as an extension (ca. 1 meter long) to fix the pollen trap to the base of the tower structure you are sampling from (Figure S1.1i). The PVC extension is a useful way to prevent animals and people working on the tower from disturbing the pollen traps.

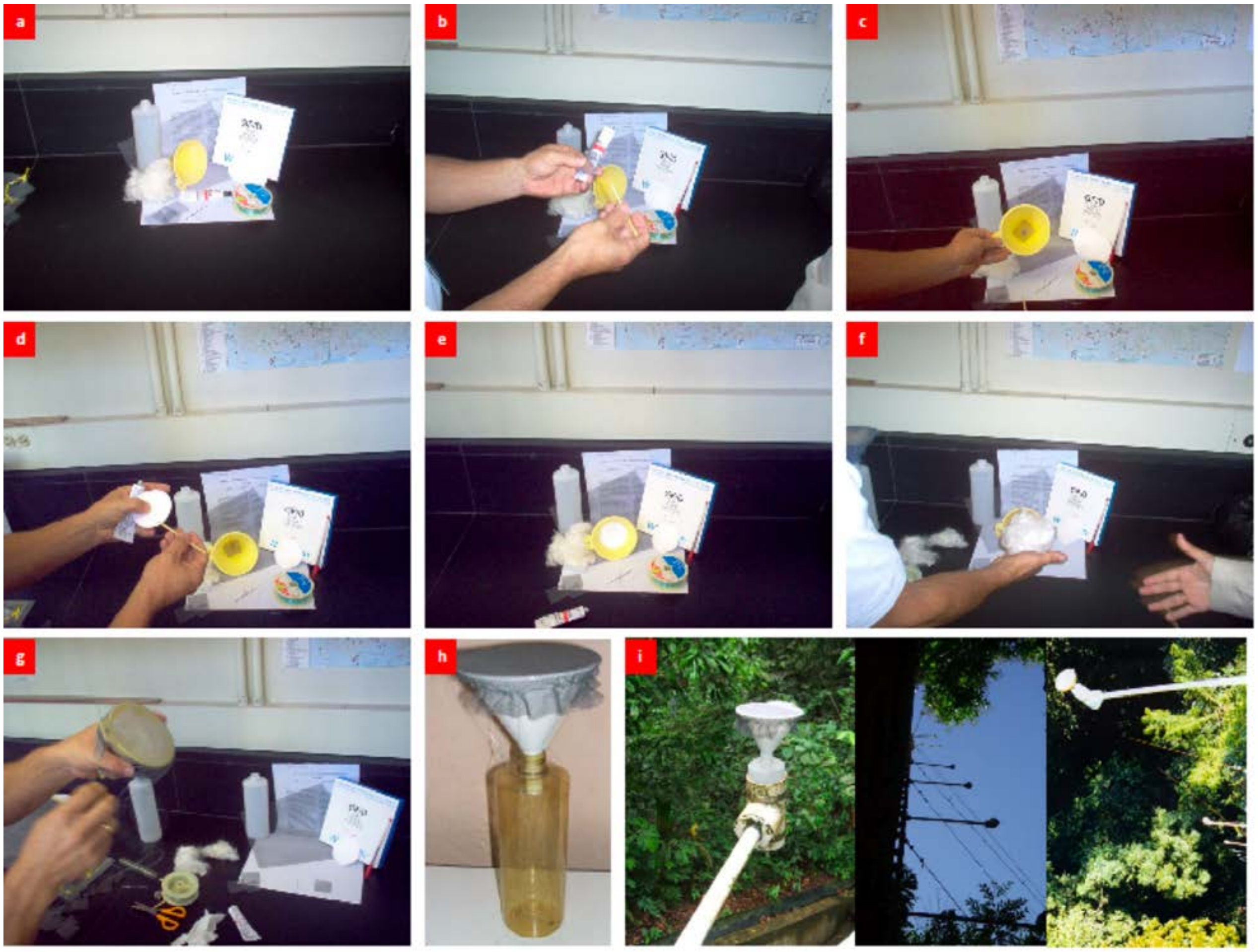

Figure S1.1. Materials required for the assembly of a pollen trap include: a plastic funnel, mesh window, silicone (glue), rayon, plastic bottle, needles, nylon, Whatman GF/D filter, and scissors (a). Glue a mesh square to the inner funnel (b,c). Glue the filter to the funnel using silicone glue (d,e). Add a small ball of rayon (f). Tie the plastic funnel to the bottle (h). Extend the pollen trap with PVC tubing (i).

Laboratory protocol

The laboratory procedure used to process the pollen samples in this study is outlined below. The procedure follows closely the laboratory procedures outlined in [32-34], with some slight modifications. Materials required for the processing of pollen samples include: a complete set of personal protection equipment (laboratory coat, goggles, gloves), fume hood, polypropylene laboratory supplies (test tubes, cylinders, beakers, stirrer rods, wash bottles), electrical equipment (centrifuge, heat-block), distilled water, chemical substances (hydrochloric acid, acetic acid, hydrofluoric acid, sulfuric acid, potassium hydroxide), mesh (500 microns), *Lycopodium* tablets, and pipettes (Figure S1.2a-d). The processing steps are as follows:

1. At the field site, transfer the rayon fiber and filter into a Whirl-Pak bag (Figure S1.2e). Store samples at a temperature of ca. 4 ºC.
2. In the laboratory, dissolve one *Lycopodium* tablet into a test tube by adding some drops of 10% Hydrochloric Acid. Afterwards, add some distilled water to neutralize.
3. Add the *Lycopodium* solution to the Whirl-Pak bag. At the same time, fill the bag with 5% Potassium Hydroxide.
4. Manually squeeze the bag several times, then pass the liquid fraction over a 500 micrometer copper mesh and recover the liquid in a 50 ml plastic tube. Concentrate the sample by centrifuging at 2700 rpm for 5 minutes. Repeat this procedure as many times as needed (Figure S1.2f).
5. To ensure that the pollen is washed completely from the filter and fiber, transfer the sample (amorphous mix of rayon and filter) to a beaker (Figure S1.2g). In the beaker, use forceps and manually separate the fiber and filter. After separating the material, wash the sample with distilled water as many times as needed until the material has changed to a clear, almost white color – the color change indicates that the organic debris and pollen have been freed from the fiber. After this has been done, take the entire liquid fraction of the sample, pass it through a copper mesh, recover the sample in the 50 ml plastic tube used previously, and concentrate it again by centrifugation.
6. Apply two more washings with distilled water and concentrations by centrifugation.
7. Transfer the sample residue into a 15 ml polypropylene test tube (Figure S1.2h).
8. De-hydrate sample with Glacial Acetic Acid prior to Acetolysis.
9. Apply an Acetolysis treatment to the sample. The Acetolysis is a 9:1 mix of Acetic Acid and Sulfuric Acid. Add the mix to the sample and heat at 100 ºC for ca. 5 minutes – the sample will change from a more transparent color to a darker color, indicating that the Acetolysis treatment was completed.
10. Wash the sample and concentrate by centrifugation three times.
11. If the sample is very dark indicating a high presence of organic matter, submit the sample to an additional treatment with 5% Potassium Hydroxide. Heat the sample for 5 minutes, then wash and concentrate with distilled water and centrifugation two more times.
12. Add concentrated Hydrofluoric Acid to the sample and heat for 5 minutes. This will remove any wind-blown or mineral deposits and ensures the complete removal of any remaining Rayon micro-fibers from the sample.
13. Treat the sample with 10% Hydrochloric Acid to eliminate all possible flocculates produced by the Hydrofluoric Acid.
14. Wash the sample with distilled water two more times. Fill the test tube with absolute alcohol for sample de-hydration and decant the sample into a 5 ml plastic vial. Remove the alcohol by centrifugation and dry the sample at 60 ºC using a temperature controlled oven.

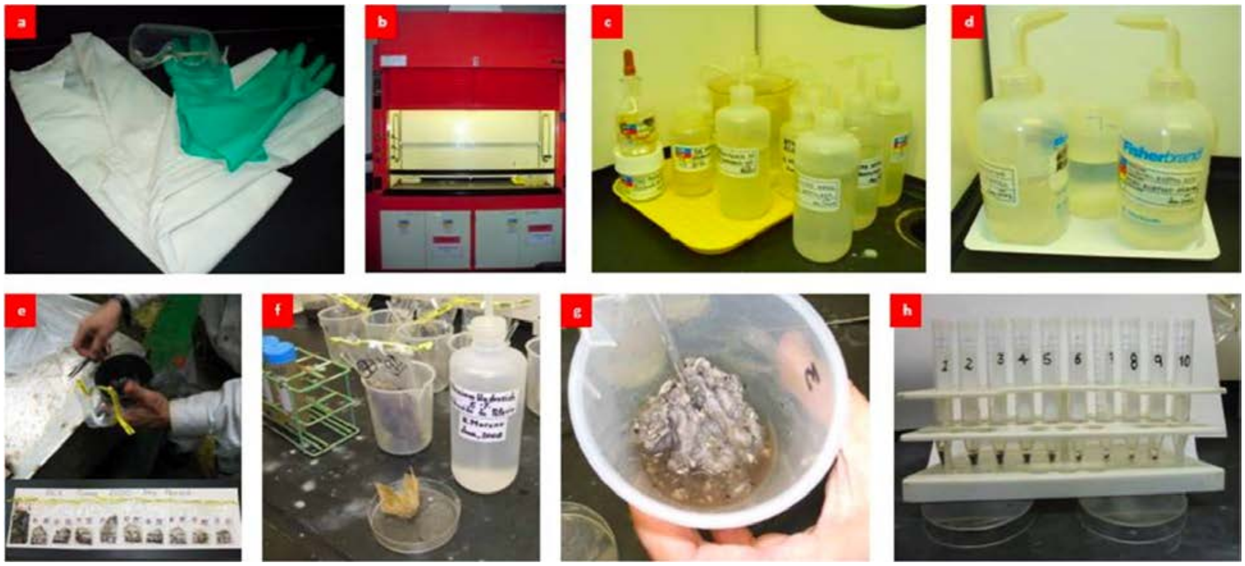

Figure S1.2. Materials required for pollen sample processing include: safety equipment, a fume hood, processing chemicals, and distilled water (a-d). Transfer the pollen samples (rayon fiber and filter) to Whirl-pak bags in the field (e). Separate the liquid fraction from the rayon and filter by manually squeezing the sample and isolating the liquid in 50 ml test tubes (f). To completely separate the sample from the rayon and filter, wash the sample with distilled water until the sample is a clear, almost white color (g). Samples are transferred to 15 ml polypropylene test tubes (h) prior to acetolysis.

Pollen mounting protocol

Depending on the amount of pollen residue, try to make two permanent slides from each sample, the first using a Silicone Oil (Dow Corning 200 Fluid, 1000 cs viscosity) mounting media and the second using a Jelly Glycerin mounting media. There are benefits and downsides to each. The Silicone Oil procedure takes a relatively long time to complete due to the time it takes for the sealed slide to dry. It is also a bit more difficult to obtain a homogenously mixed sample compared to Jelly Glycerin, but the overall preservation of the sample is superior. While Jelly Glycerin is a much more quick and economic alternative, prolonged exposure of pollen grains to the mounting medium can cause grains to swell and increase in size between 20-25% of the time. A short summary of each procedure is outlined below:

Silicone procedure:

1. Place a small drop of Silicone Oil on a pre-cleaned glass microscopy slide.
2. Take an aliquot of the pollen sample with a pipette and place it on the silicone drop.

3. Warm the sample on a heater plate (ca. 50 ºC) and mix the sample so that it is homogenously dispersed.
4. Place a glass cover slide over the top of the sample and keep the slide positioned horizontally until the silicone dries – a process that can take weeks.

Jelly Glycerin procedure:

1. Add some drops of Glycerin (Glycerol) to the 5 ml polypropilene sample vial and mix the pollen residue with a laboratory needle. Heat the vial at 60 ºC in an oven until the alcohol has evaporated.
2. Add a small amount of Jelly Glycerin on a pre-cleaned glass microscopy slide (Figure S1.3a).
3. Take an aliquot of the pollen sample with a pipette and place it on the Jelly Glycerin (Figure S1.3b,c).
4. Warm the sample on a heater plate (ca. 50 ºC) and mix the sample so that it is homogenously dispersed (Figure S1.3d).
5. Place a glass cover slide over the top of the sample (Figure S1.3e).
6. Place the slide back on the heater plate and seal the cover of the slide with a liquid hot paraffin sealant (Figure S1.3f).
7. After the paraffin has dried, clean up and remove the solid remnants with a blade.
8. Clean off the slide.
9. Add nail polish to the edges of the cover slide to further seal the sample.

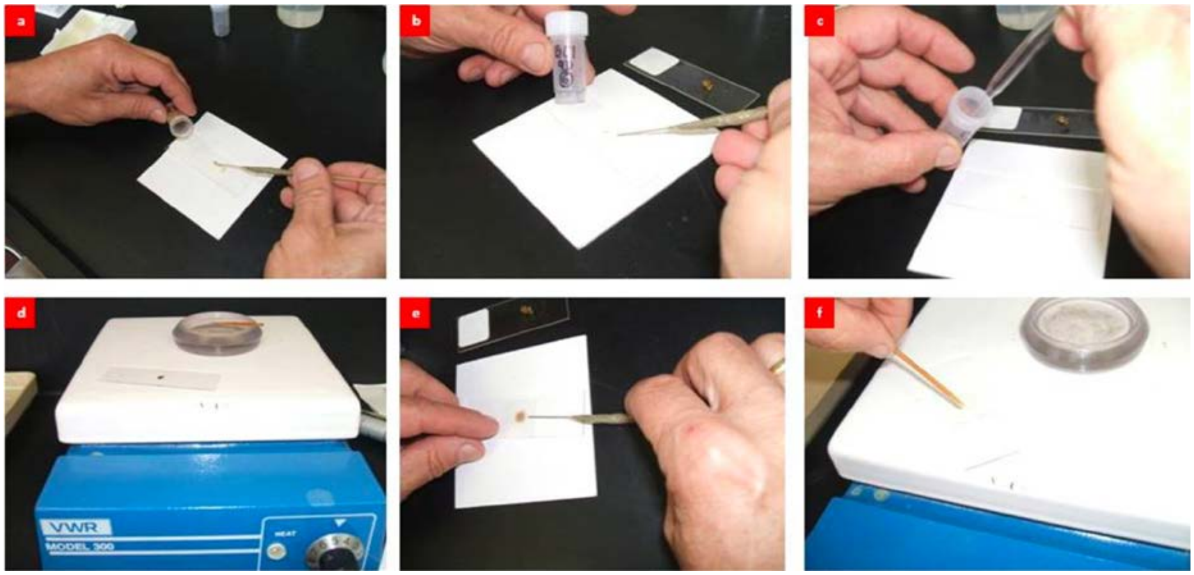

Figure S1.3. If using the jelly glycerin mounting medium, add a small amount on to a clean microscope slide (a). Pipette an aliquot of the pollen sample on to the jelly glycerin (b,c). Warm and homogenously mix the sample and jelly glycerin on a heater plate (d). Add a clean glass cover slip to the slide (e). Seal the slide with paraffin on the heater plate (f).
