# Supplementary material for: Variability within the 10-Year Pollen Rain of a Seasonal Neotropical Forest and Its Implications for Paleoenvironmental and Phenological Research
Source: PLoS One. 2013 Jan 8;8(1):e53485. doi: 10.1371/journal.pone.0053485 (PMC3540050; doi:10.1371/journal.pone.0053485)
Supplement: Appendix S2 — Plate images of named and unknown pollen types, Part I (Supporting Plates S1–S22). Scale bars represent 20 µm. Multiple images are provided to highlight the texture and cross-sectional shape of each grain. Images were taken using a Plan-Apochromat SF25 (63×, 1.4NA, oil immersion) lens and a Zeiss AxioCam ICc 3 digital microscope camera. (PDF) [file pone.0053485.s009.pdf]

SUPPORTING INFORMATION

HASELHORST, MORENO AND PUNYASENA

Variability within the 10-year pollen rain of a seasonal Neotropical forest and its implications for paleoenvironmental and phenological research

Appendix S2: Supporting Plates S1-S22. Plate images of named and unknown pollen types, Part I. Scale bars represent 20 µm. Multiple images are provided to highlight the texture and cross-sectional shape of each grain. Images were taken using a Plan-Apochromat SF25 (63x, 1.4NA, oil immersion) lens and a Zeiss AxioCam ICc 3 digital microscope camera.

Plate S1. Acanthaceae: *Mendoncia gracilis* (A1-A3, image scaled 50%); Amaranthaceae: *Chamisoa* sp. (B1-B3); *Chenopodium* sp. (C1-C3)

Plate S2. Anacardiaceae: *Anacardium* sp. (A1-A6); *Astronium graveolens* (B1-B3)

Plate S3. Anacardiaceae: *Spondias* sp.1 (A1-A3, image scaled 75%); *Spondias* sp.2 (B1-B3); cf. Anacardiaceae sp. (C1-C3, image scaled 75%); Aquifoliaceae: *Ilex* sp. (D1-D3)

Plate S4. Apocynaceae: *Lacmellea panamensis* (A1-A3); Araceae: *Anthurium* sp.1 (B1-B3); *Anthurium* sp.2 (C1-C3); Araliaceae: *Dendropanax* sp. (D1-D3); *Schefflera* sp. (E1-E3)

Plate S5. Arecaceae: Arecaceae spp. (A1-A6); cf. *Astrocaryum* sp. (A1-A3); cf. *Oenocarpus* sp. (A4-A6)

Plate S6. Arecaceae: *Cryosophila* sp. (A1-A3); *Socratea* sp. (B1-B2); Asteraceae: sp.2 (C1-C3)

Plate S7. Asteraceae: sp.1 (A1-A3); Betulaceae: *Alnus* sp. (B1-B4)

Plate S8. Bignoniaceae: *Arrabidaea* sp.1 (A1-A6)

Plate S9. Bignoniaceae: *Arrabidaea* sp.2 (B1-B4, image scaled 75%); *Cydista* sp. (C1-C3, image scaled 75%); *Jacaranda* sp. (A1-A3, image scaled 75%)

Plate S10. Bignoniaceae: cf. *Mansoa* sp. (A1-A3, image scaled 50%) *Tabebuia* sp. (B1-B4, image scaled 75%)

Plate S11. Boraginaceae: *Cordia* sp. (A1-A6)

Plate S12. Burseraceae: *Bursera simaruba* (A1-A3); cf. *Bursera simaruba* var. (B1-B6)

Plate S13. Burseraceae: *Protium* sp. (A1-A3); Cannabaceae: *Celtis* sp. (B1-B4); *Trema* sp. (C1-C3)

Plate S14. Chloranthaceae: *Hedyosmum* sp. (A1-A6); Combretaceae: *Combretum* sp.1 (B1-B3); *Combretum* sp.2 (C1-C3)

Plate S15. Convolvulaceae: *Maripa* sp. (A1-A3, image scaled 75%); Cucurbitaceae: *Melothria* sp. (B1-B3); Euphorbiaceae: *Acalypha* spp. (C1-C6)

Plate S16. Euphorbiaceae: *Alchornea costaricensis* (A1-A6); *Alchornea latifolia* (B1-B3); *Chamaesyce* sp.2 (D1-D3); *Chamaesyce* sp.3 (E1-E3); *Chamaesyce* sp.1 (C1-C3)

Plate S17. Euphorbiaceae: *Croton* sp. (A1-A3, image scaled 50%); *Dalechampia* sp. (B1-B4, image scaled 50%); *Hyeronima* sp. (C1-C3)

Plate S18. Euphorbiaceae: *Sapium* sp. (B1-B4, image scaled 75%)

Plate S19. Euphorbiaceae: cf. *Alchornea* sp. (C1-C6); unknown sp.1 (B1-B3); unknown sp.2 (A1-A4)

Plate S20. Fabaceae (Mimosoideae): cf. *Inga* sp. (A1-A3, image scaled 50%); unknown sp. 2 (B1-B3, image scaled 50%)

Plate S21. Fabaceae (Papilionoideae): *Erythrina costaricensis* (A1-A3); *Machaerium* sp. (B1-B4); Flacouticaceae/Salicaceae: cf. *Laetia procera* (C1-C4, image scaled 50%)

Plate S22. Hippocrataceae: *Hippocratea volubilis* (A1-A3, image scaled 50%); Lecythidaceae: cf. *Gustavia* superba (B1-B3); Loranthaceae: *Oryctanthus* sp. (C1-C3, image scaled 50%)

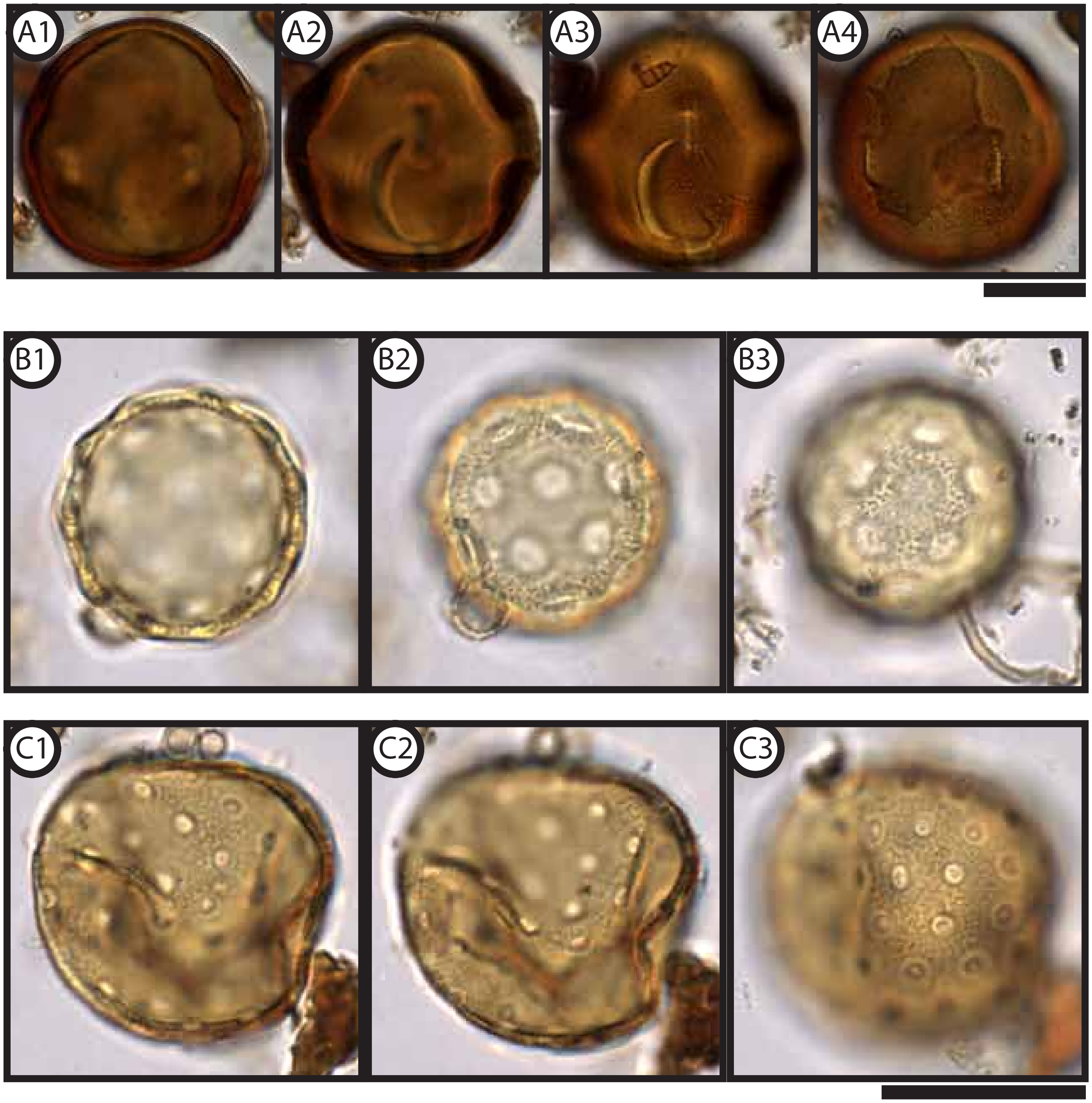

Plate S1. Acanthaceae: *Mendoncia gracilis* (A1-A3, image scaled 50%); Amaranthaceae: *Chamisoa* sp. (B1-B3); *Chenopodium* sp. (C1-C3)

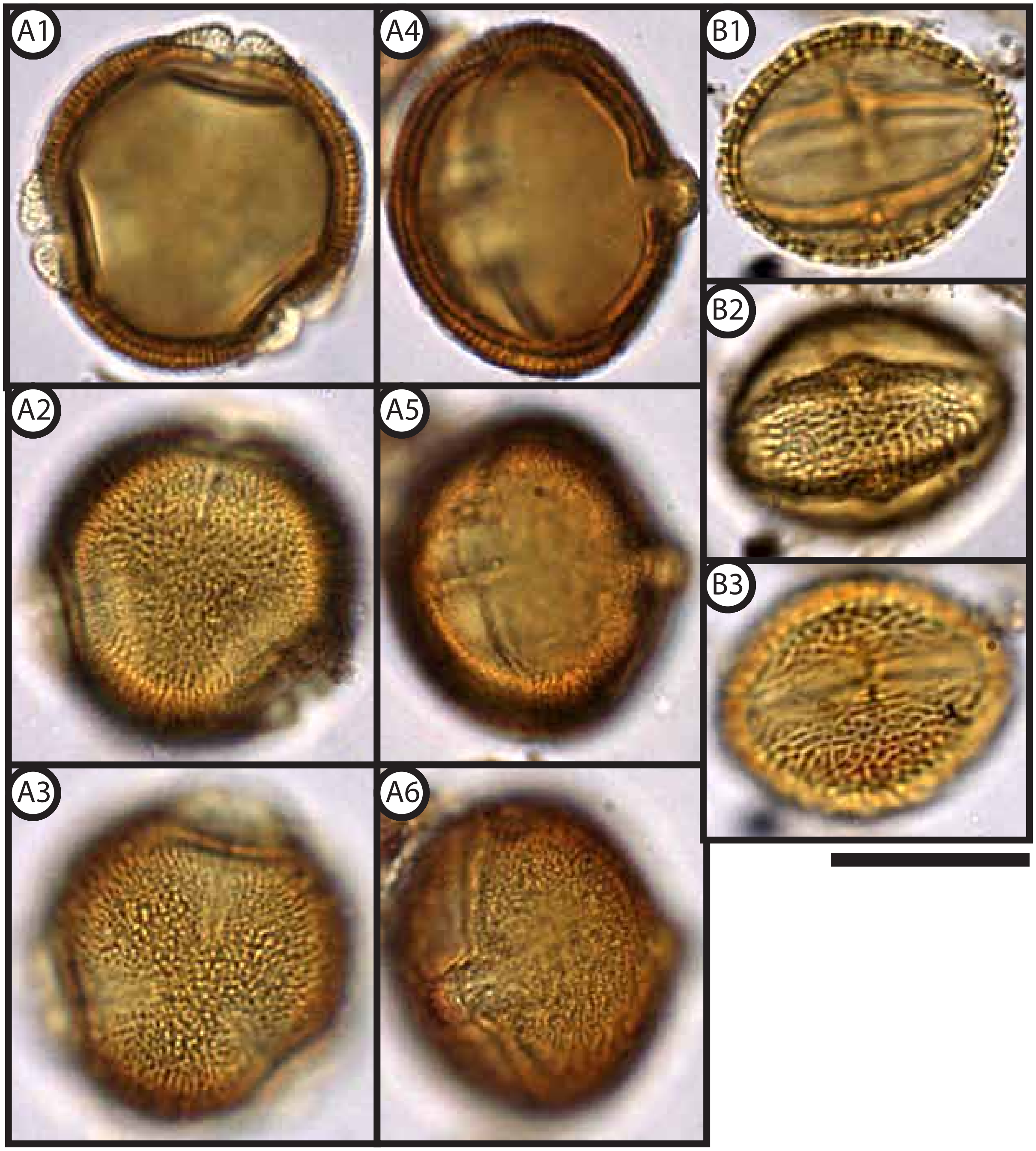

Plate S2. Anacardiaceae: *Anacardium* sp. (A1-A6); *Astronium graveolens* (B1-B3)

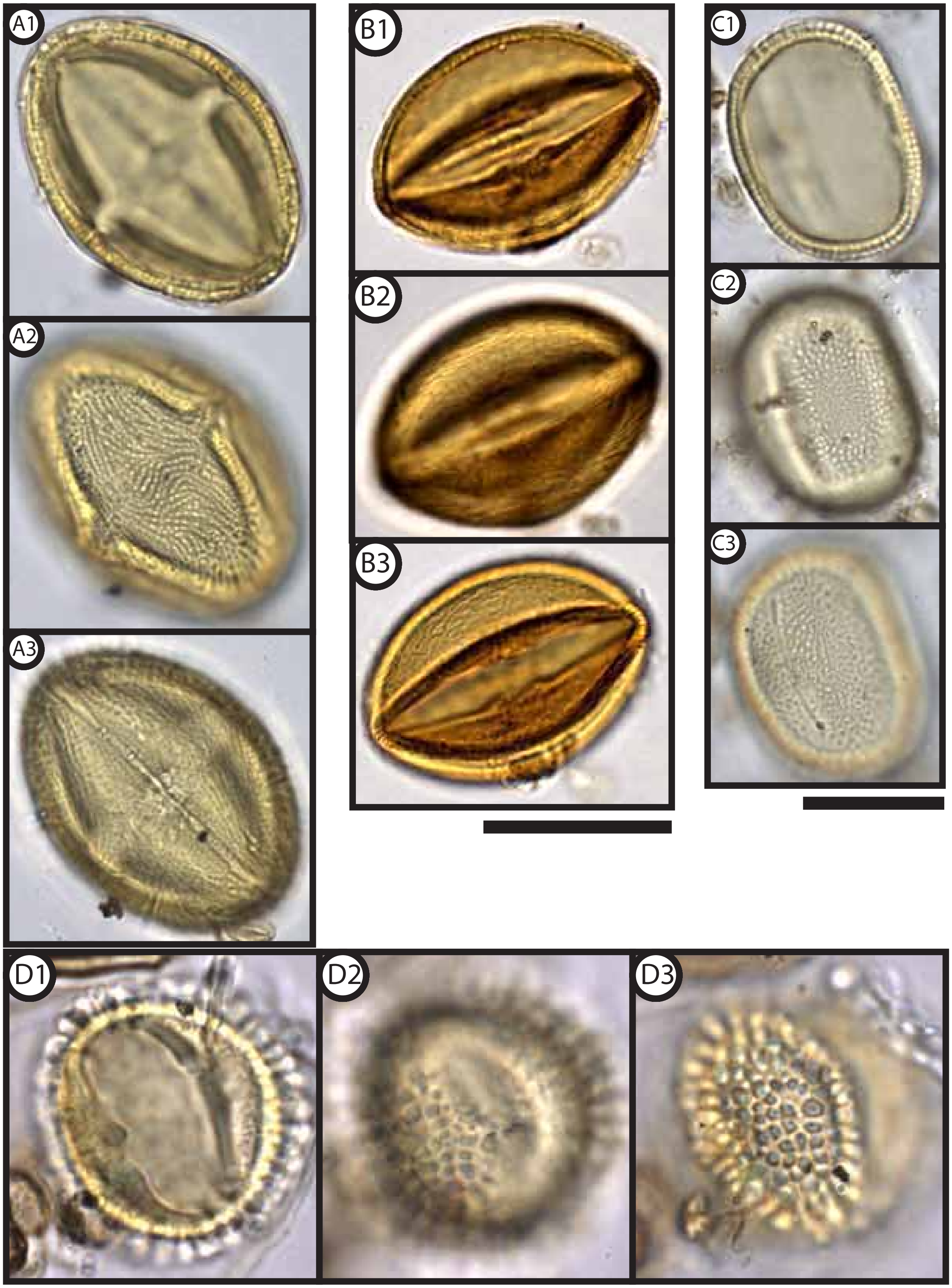

Plate S3. Anacardiaceae: *Spondias* sp.1 (A1-A3, image scaled 75%); *Spondias* sp.2 (B1-B3); cf. Anacardiaceae sp. (C1-C3, image scaled 75%); Aquifoliaceae: *Ilex* sp. (D1-D3)

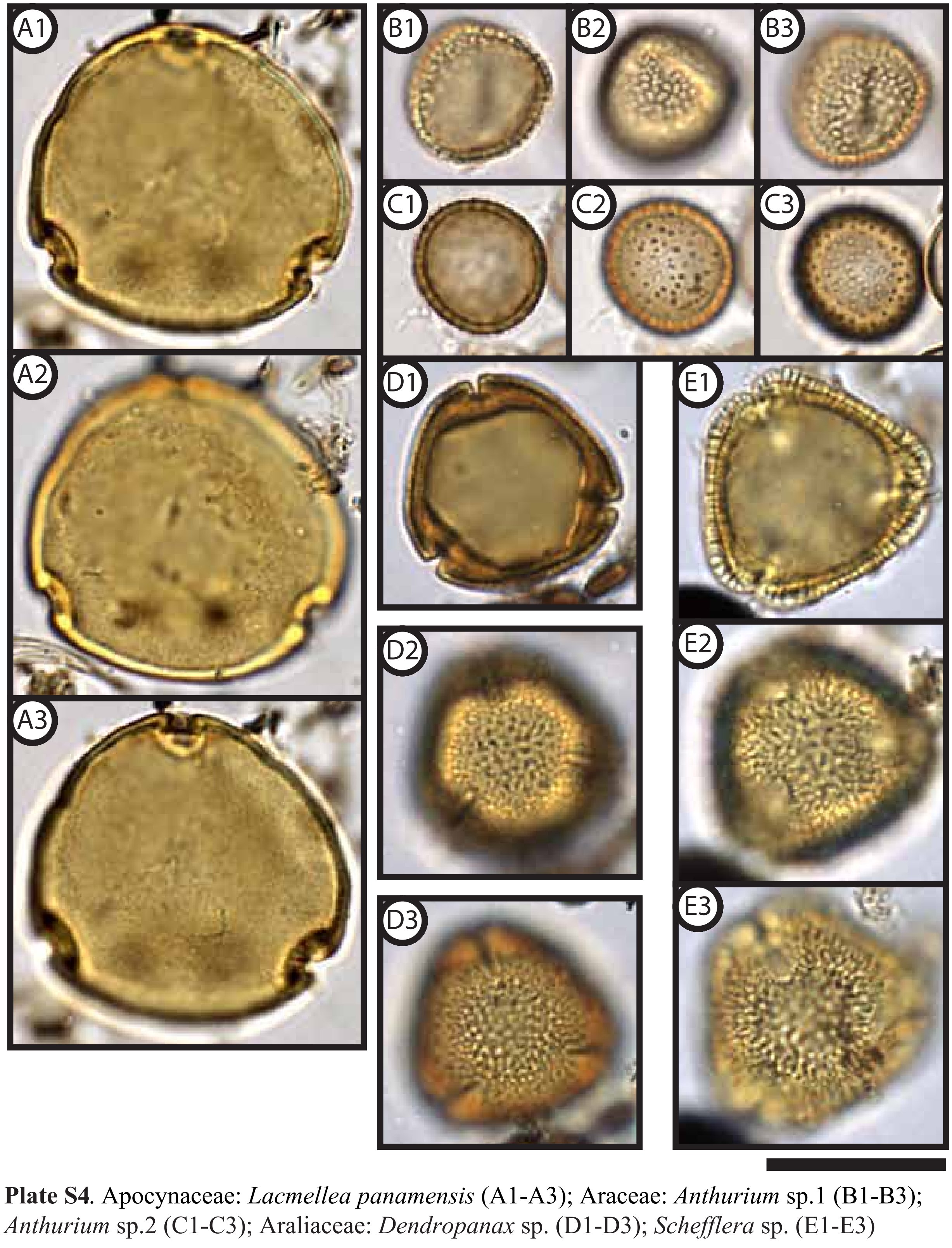

Plate S4. Apocynaceae: *Lacmellea panamensis* (A1-A3); Araceae: *Anthurium* sp.1 (B1-B3); *Anthurium* sp.2 (C1-C3); Araliaceae: *Dendropanax* sp. (D1-D3); *Schefflera* sp. (E1-E3)

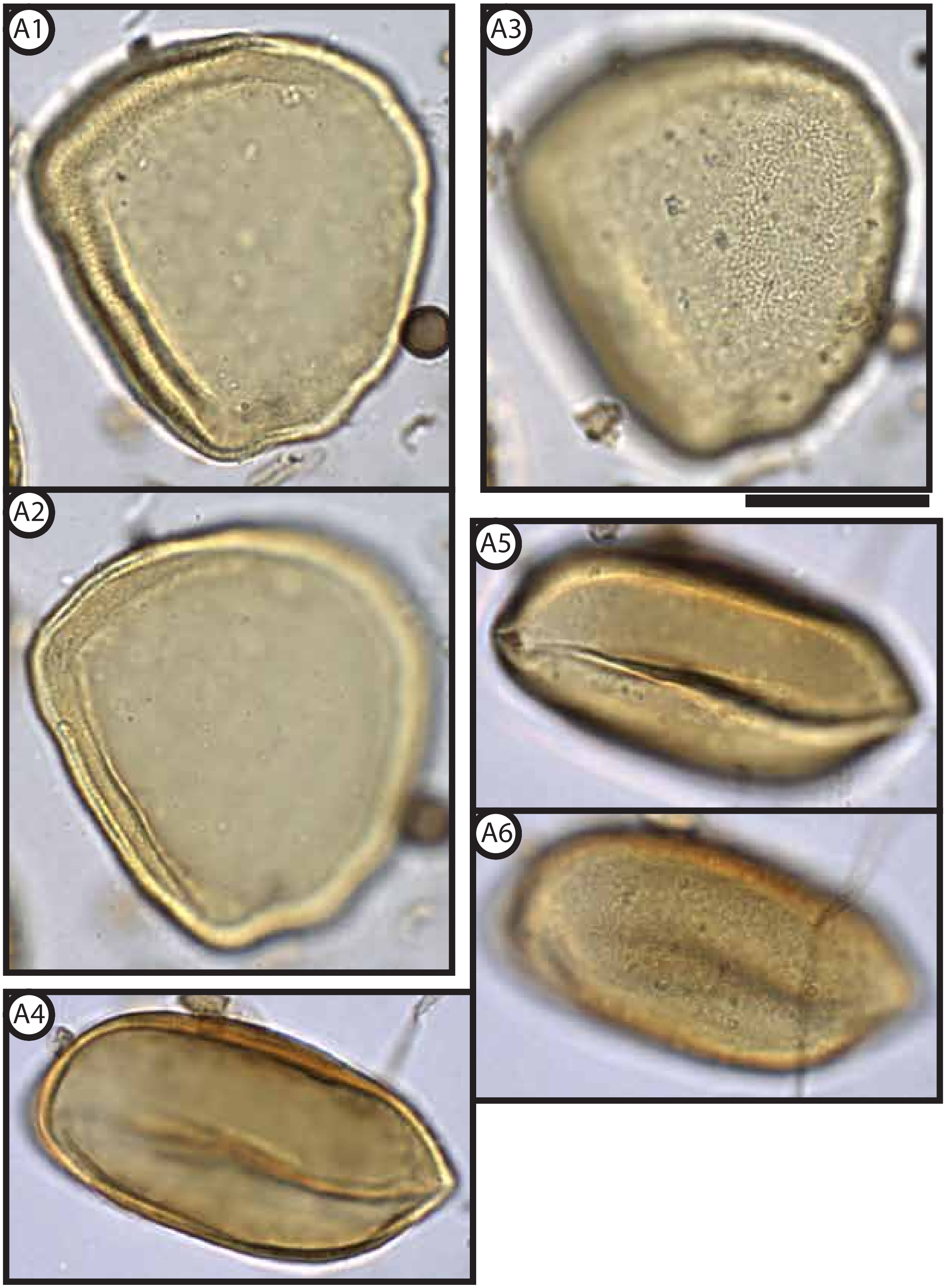

Plate S5. Arecaceae: Arecaceae spp. (A1-A6); cf. *Astrocaryum* sp. (A1-A3); cf. *Oenocarpus* sp. (A4-A6)

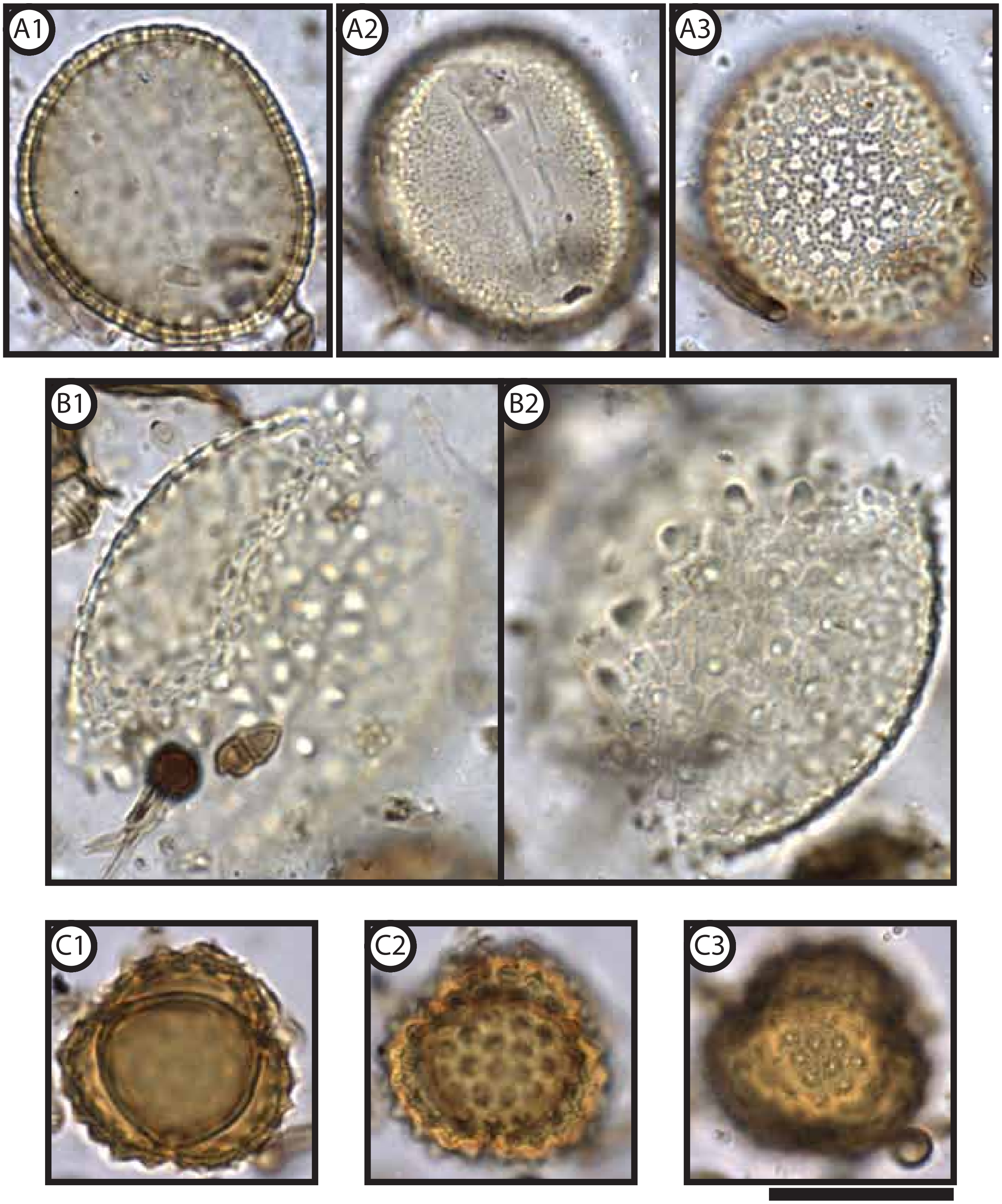

Plate S6. Arecaceae: *Cryosophila* sp. (A1-A3); *Socratea* sp. (B1-B2); Asteraceae: sp.2 (C1-C3)

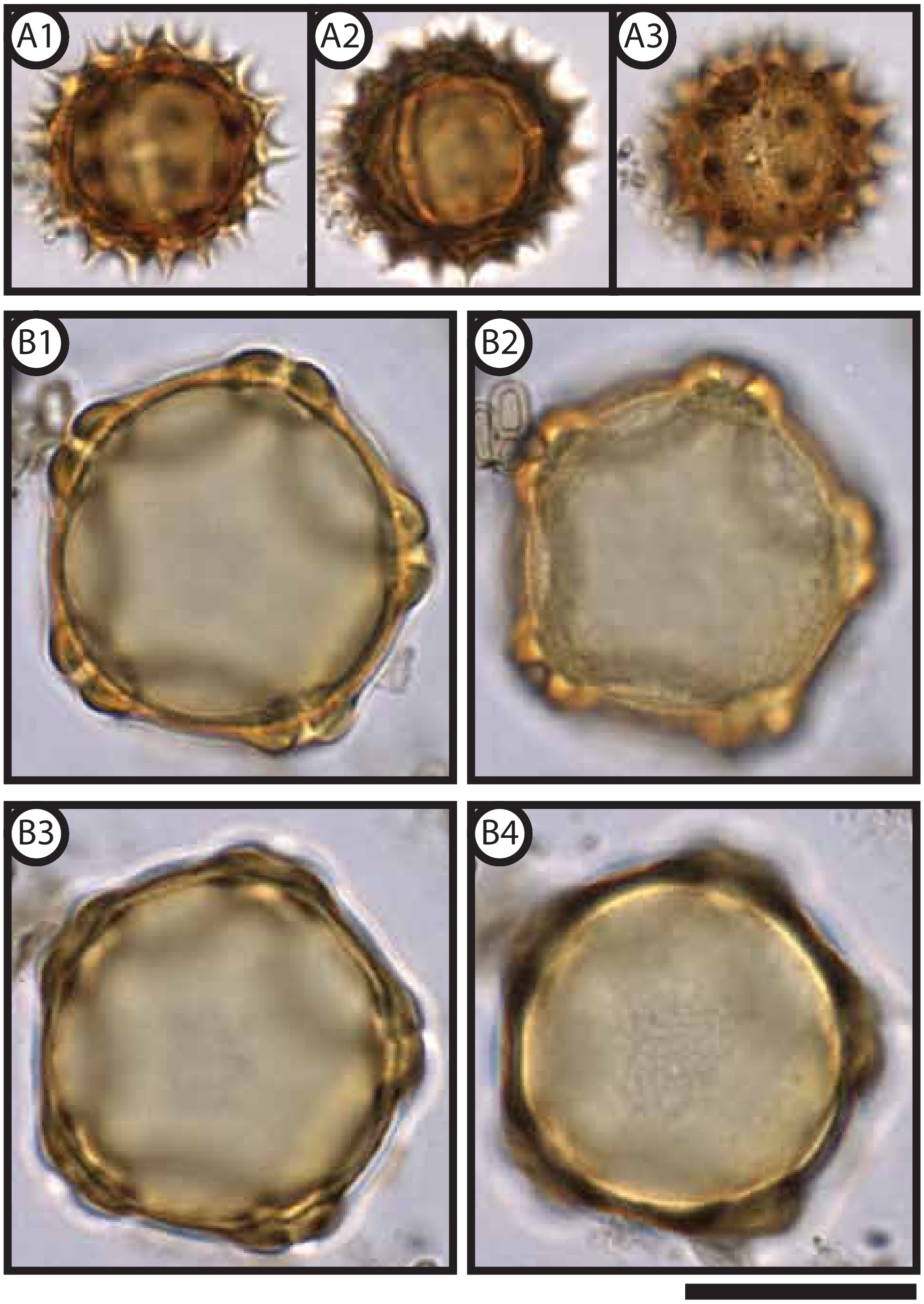

Plate S7. Asteraceae: sp.1 (A1-A3); Betulaceae: *Alnus* sp. (B1-B4)

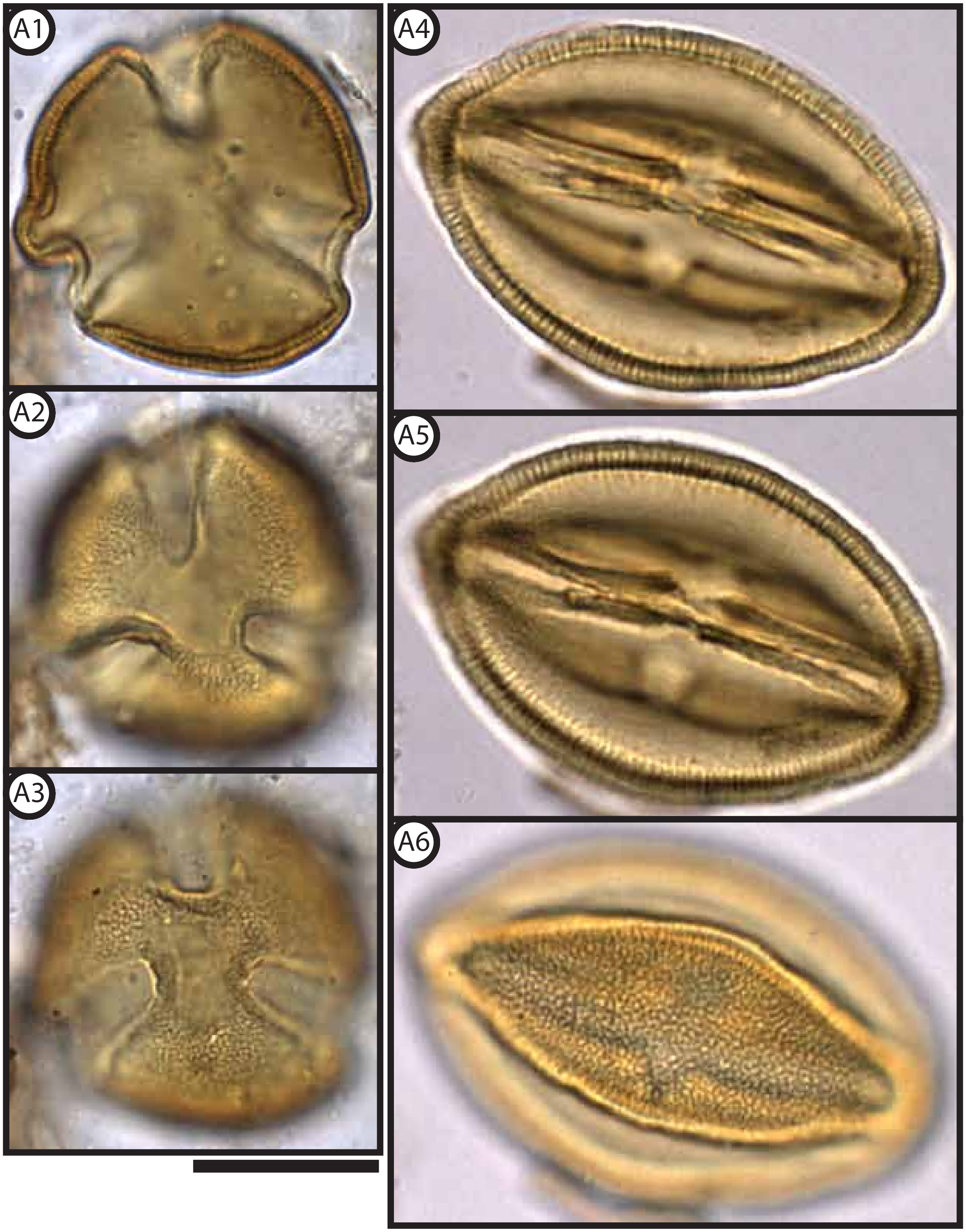

Plate S8. Bignoniaceae: *Arrabidaea* sp.1 (A1-A6)

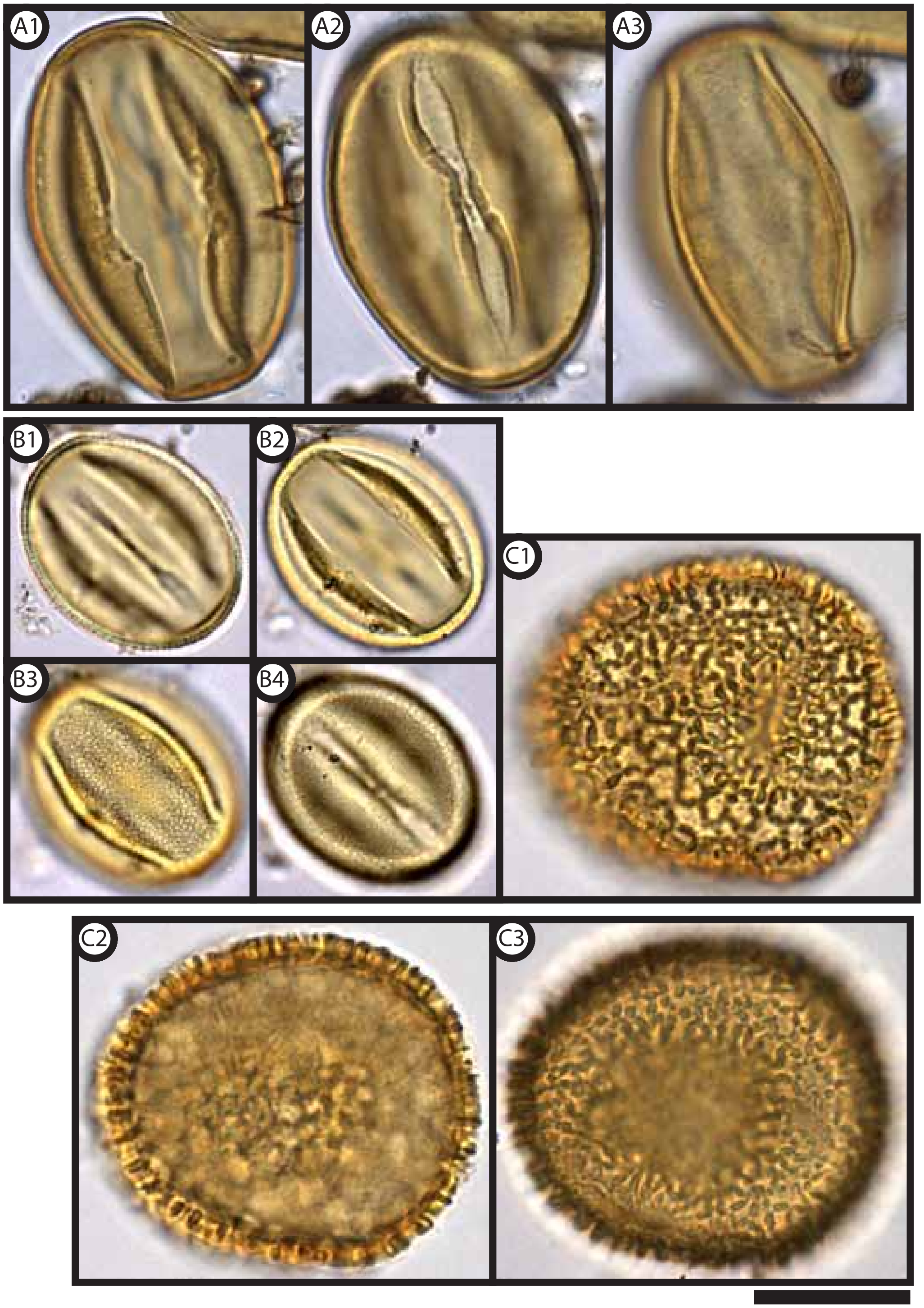

Plate S9. Bignoniaceae: *Arrabidaea* sp.2 (B1-B4, image scaled 75%); *Cydista* sp. (C1-C3, image scaled 75%); *Jacaranda* sp. (A1-A3, image scaled 75%)

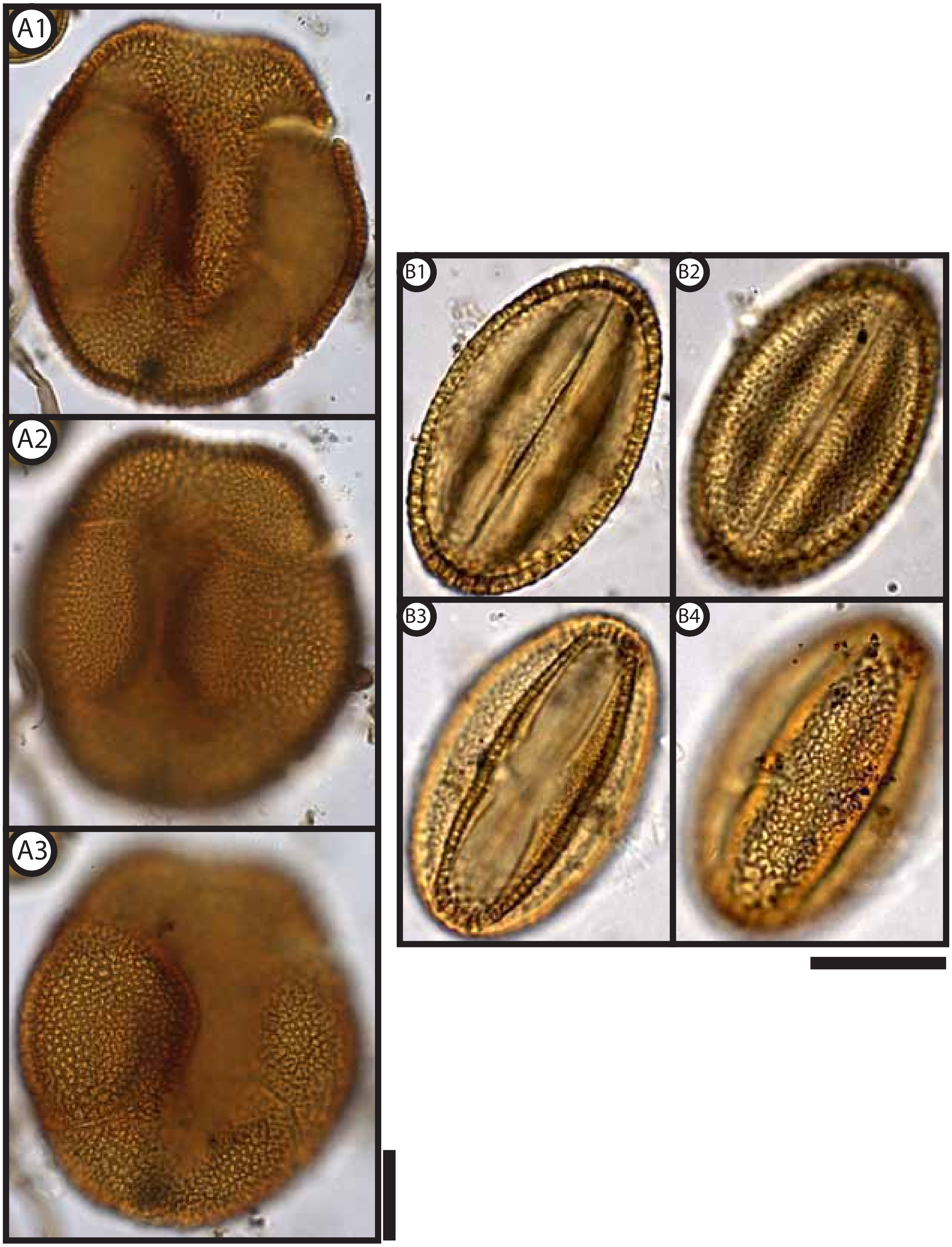

Plate S10. Bignoniaceae: cf. *Mansoa* sp. (A1-A3, image scaled 50%)
Tabebuia sp. (B1-B4, image scaled 75%)

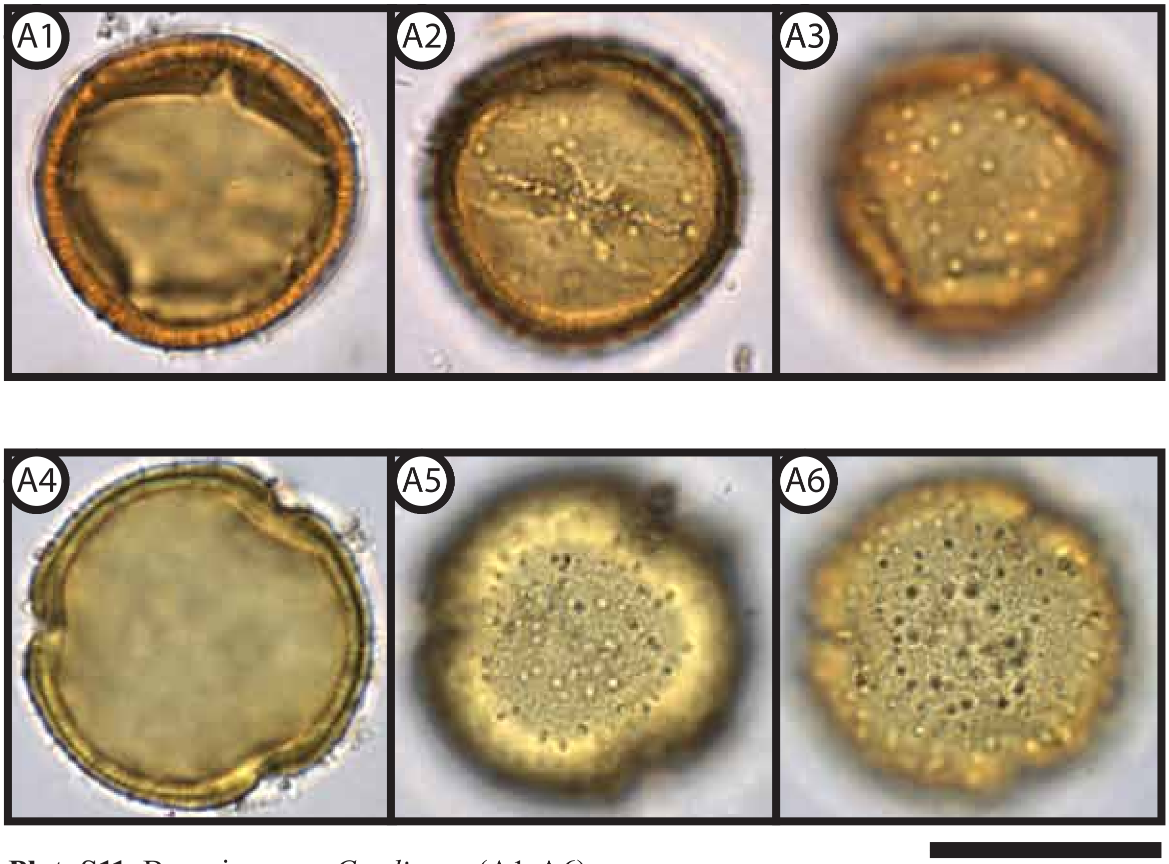

Plate S11. Boraginaceae: *Cordia* sp. (A1-A6)

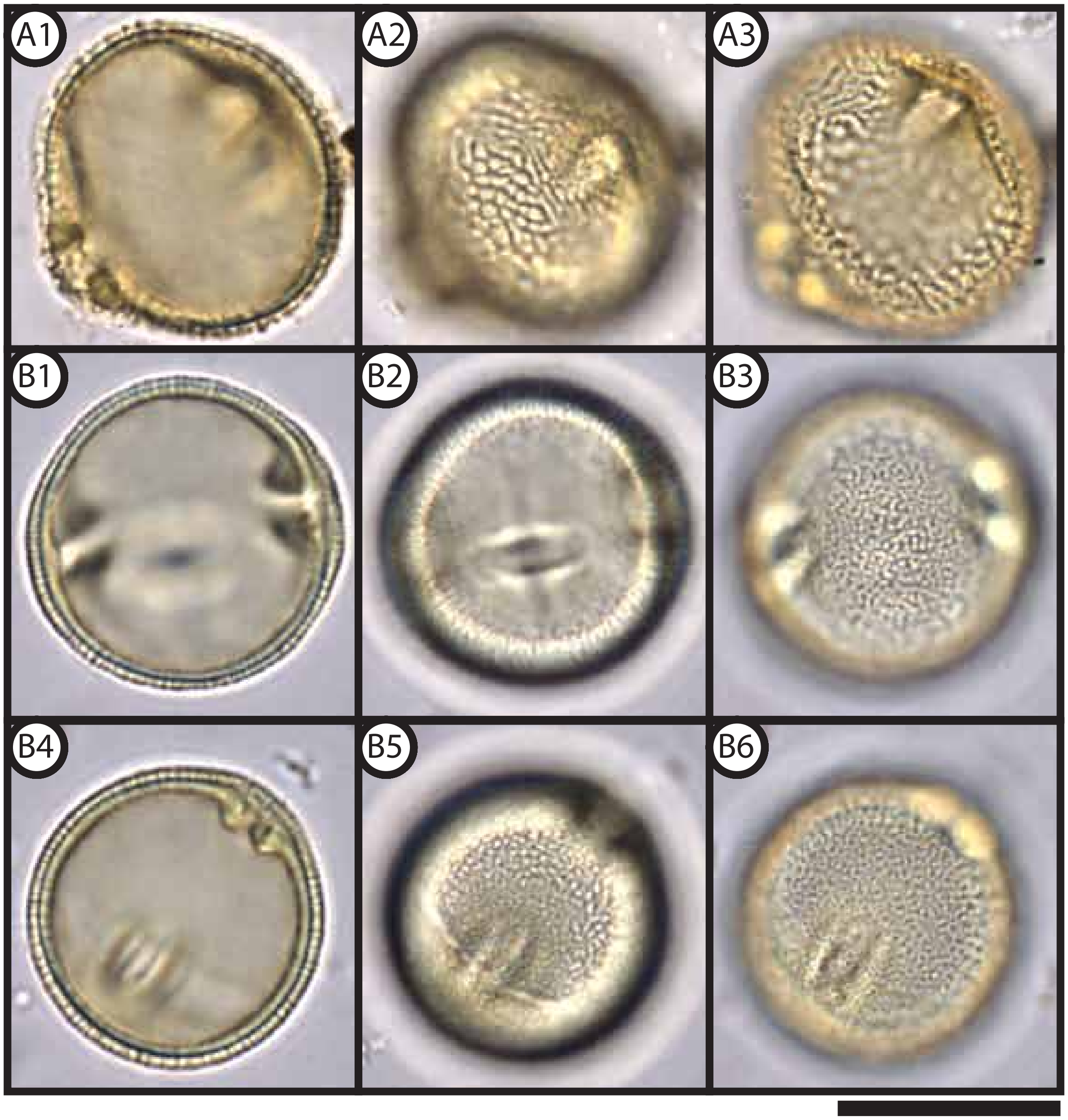

Plate S12. Burseraceae: *Bursera simaruba* (A1-A3); cf. *Bursera simaruba* var. (B1-B6)

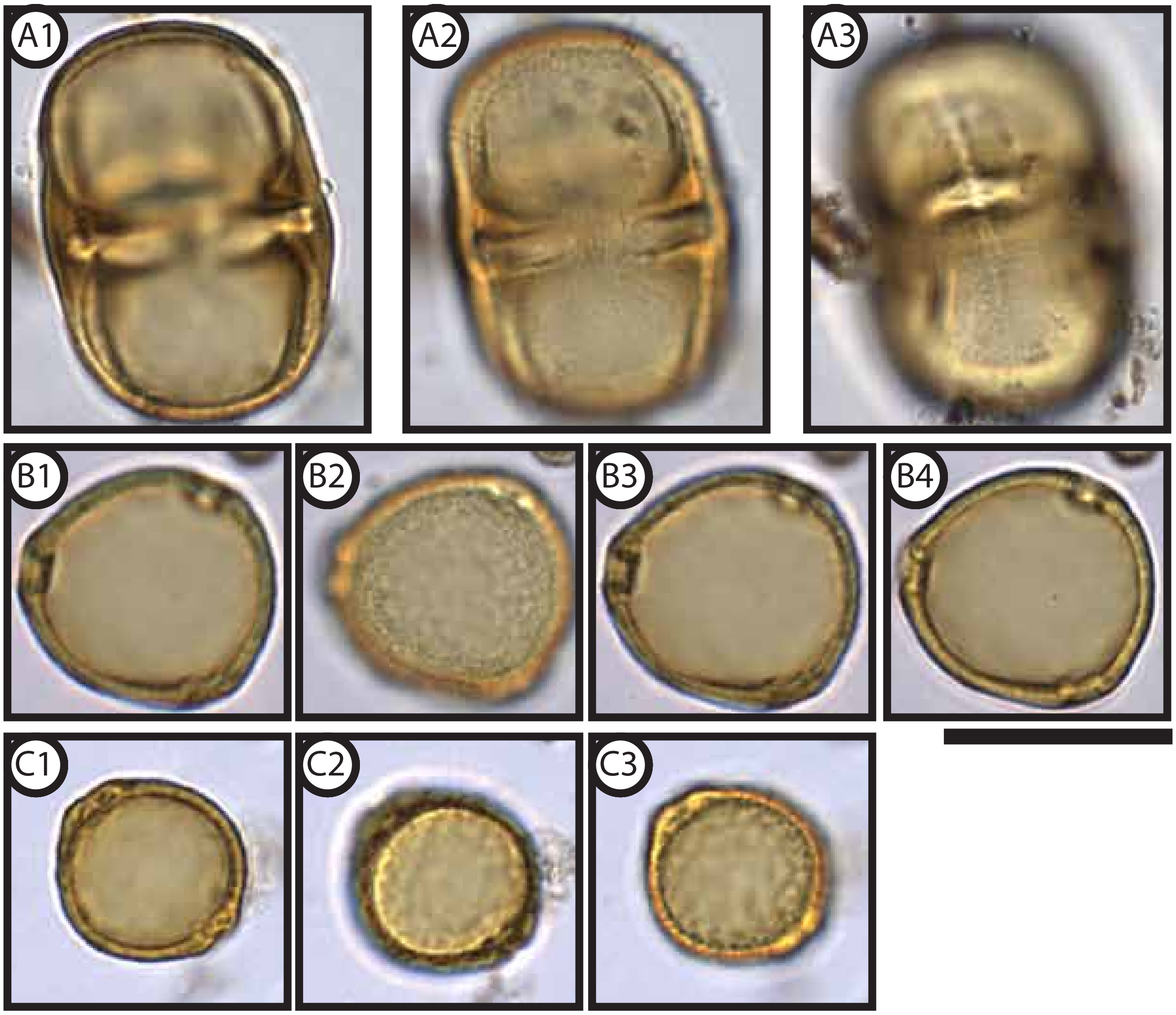

Plate S13. Burseraceae: *Protium* sp. (A1-A3); Cannabaceae: *Celtis* sp. (B1-B4); *Trema* sp. (C1-C3)

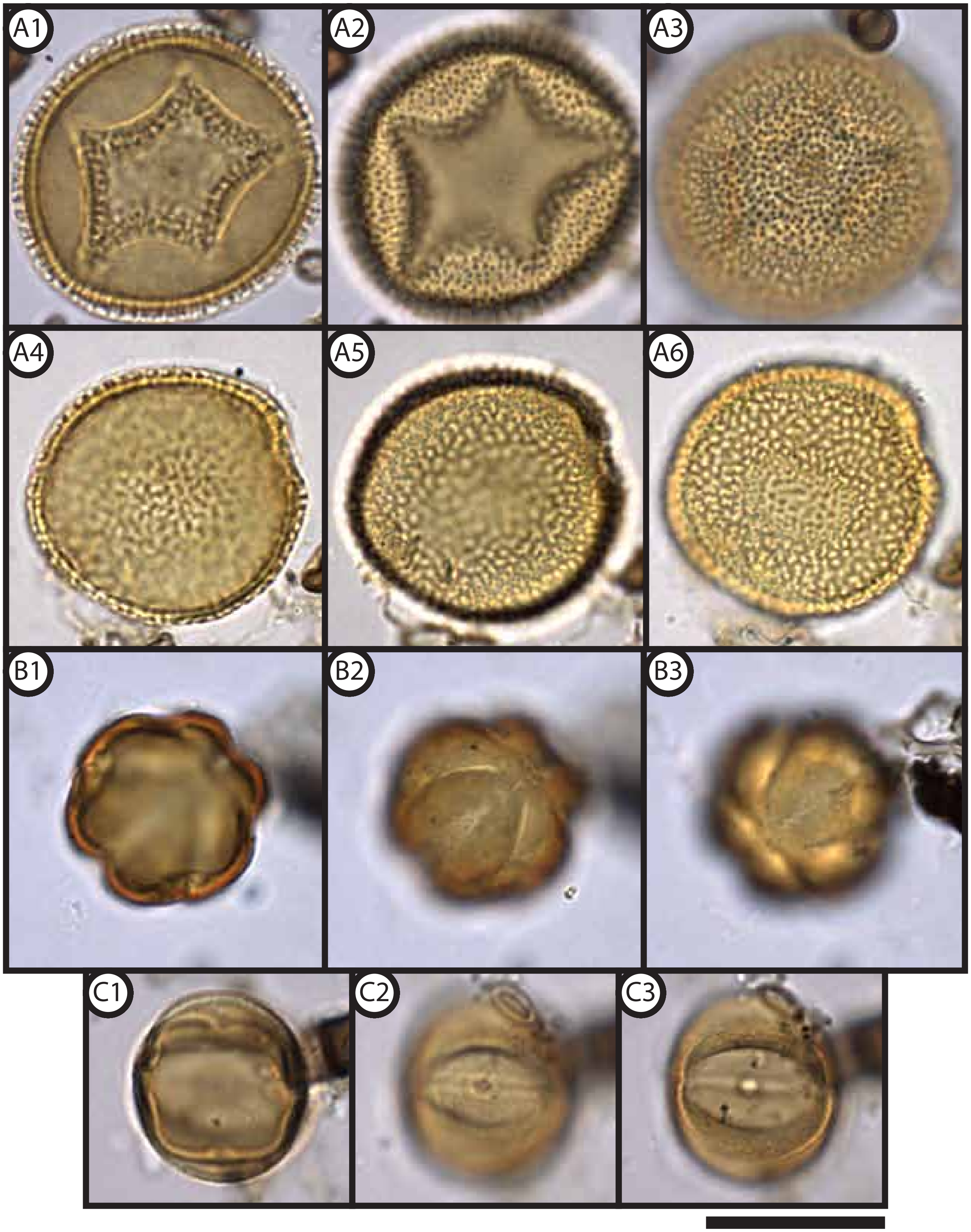

Plate S14. Chloranthaceae: *Hedyosmum* sp. (A1-A6);
Combretaceae: *Combretum* sp.1 (B1-B3); *Combretum* sp.2 (C1-C3)

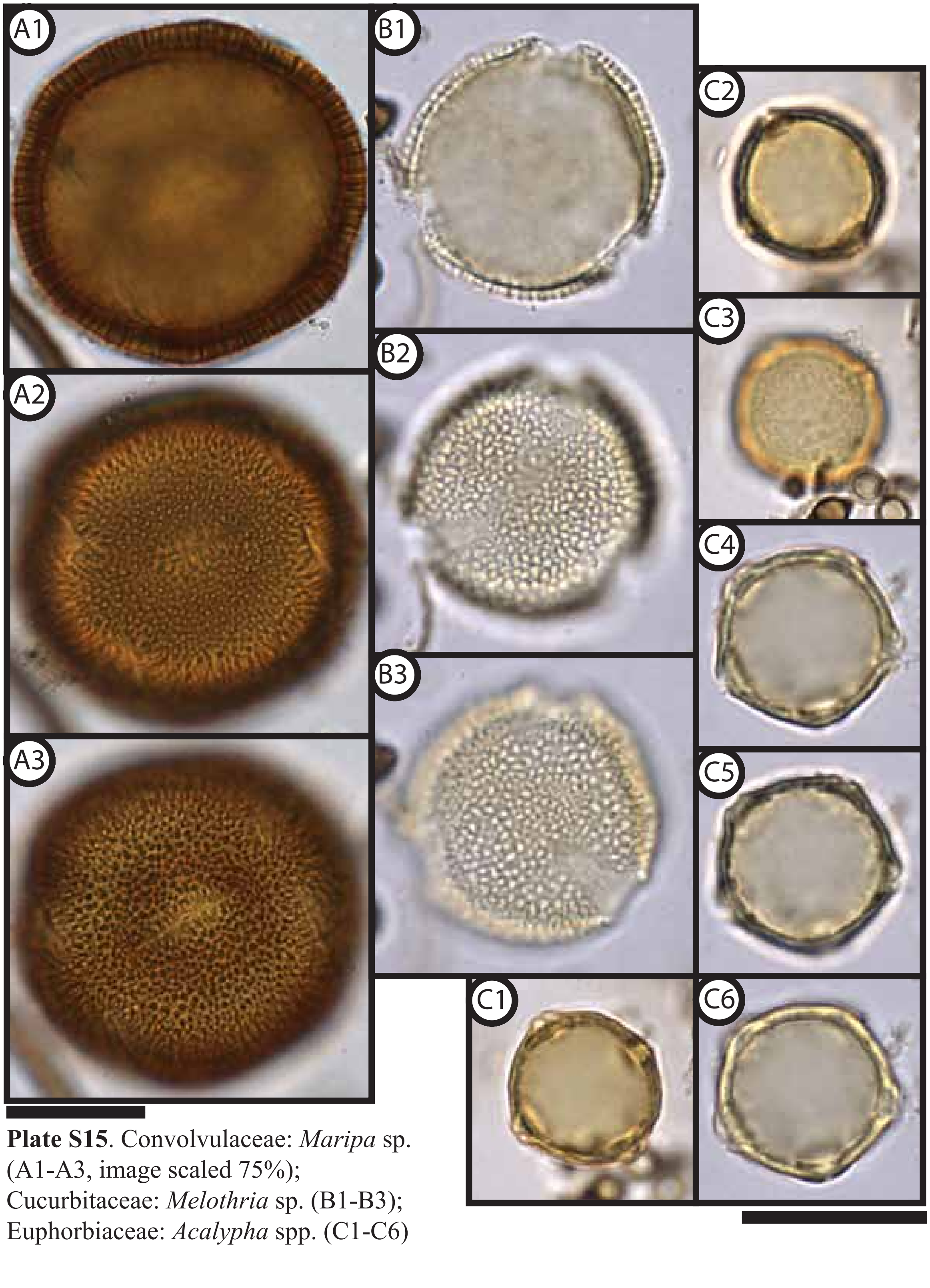

Plate S15. Convolvulaceae: *Maripa* sp. (A1-A3, image scaled 75%); Cucurbitaceae: *Melothria* sp. (B1-B3); Euphorbiaceae: *Acalypha* spp. (C1-C6)

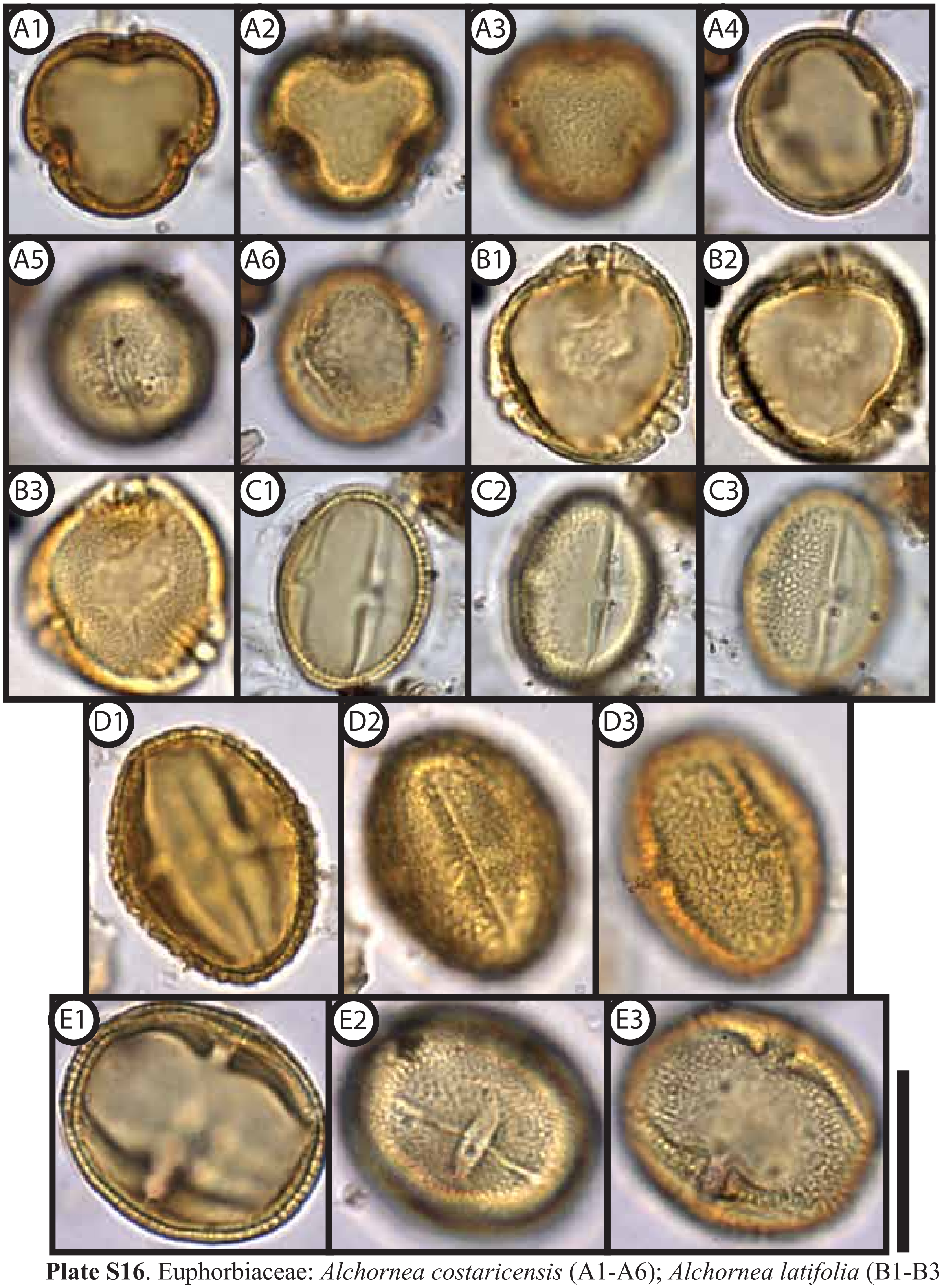

Plate S16. Euphorbiaceae: *Alchornea costaricensis* (A1-A6); *Alchornea latifolia* (B1-B3); *Chamaesyce* sp.2 (D1-D3); *Chamaesyce* sp.3 (E1-E3); *Chamaesyce* sp.1 (C1-C3)

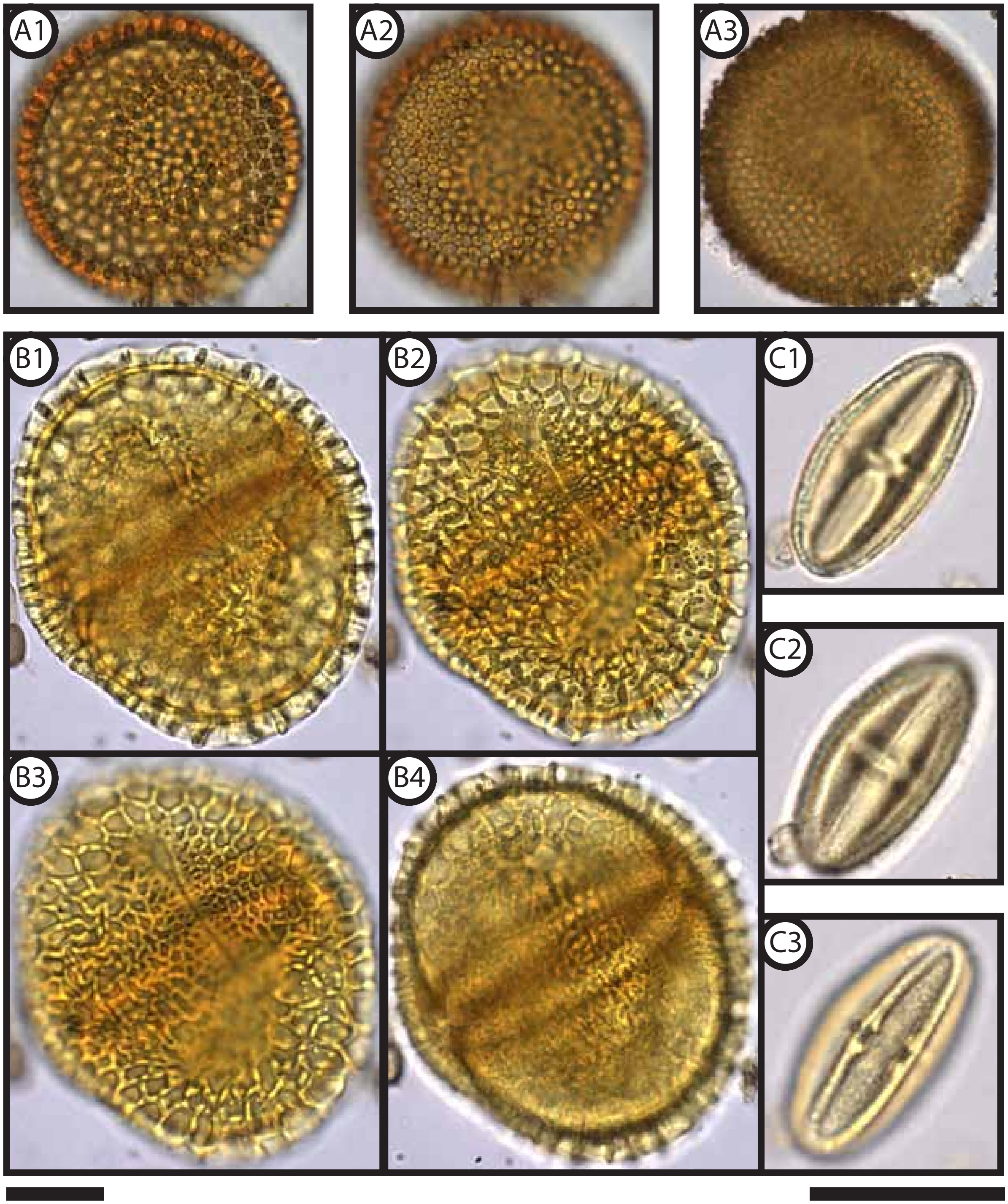

Plate S17. Euphorbiaceae: *Croton* sp. (A1-A3, image scaled 50%); *Dalechampia* sp. (B1-B4, image scaled 50%); *Hyeronima* sp. (C1-C3)

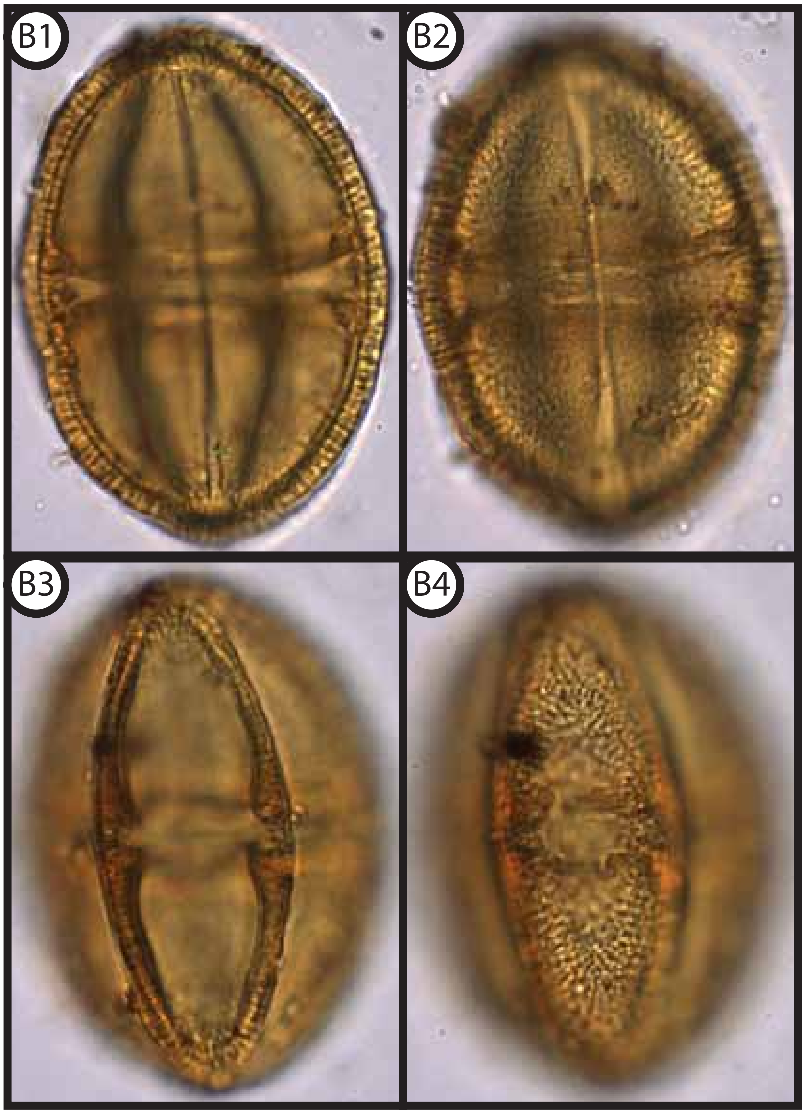

Plate S18. Euphorbiaceae: *Sapium* sp.
(B1-B4, image scaled 75%)

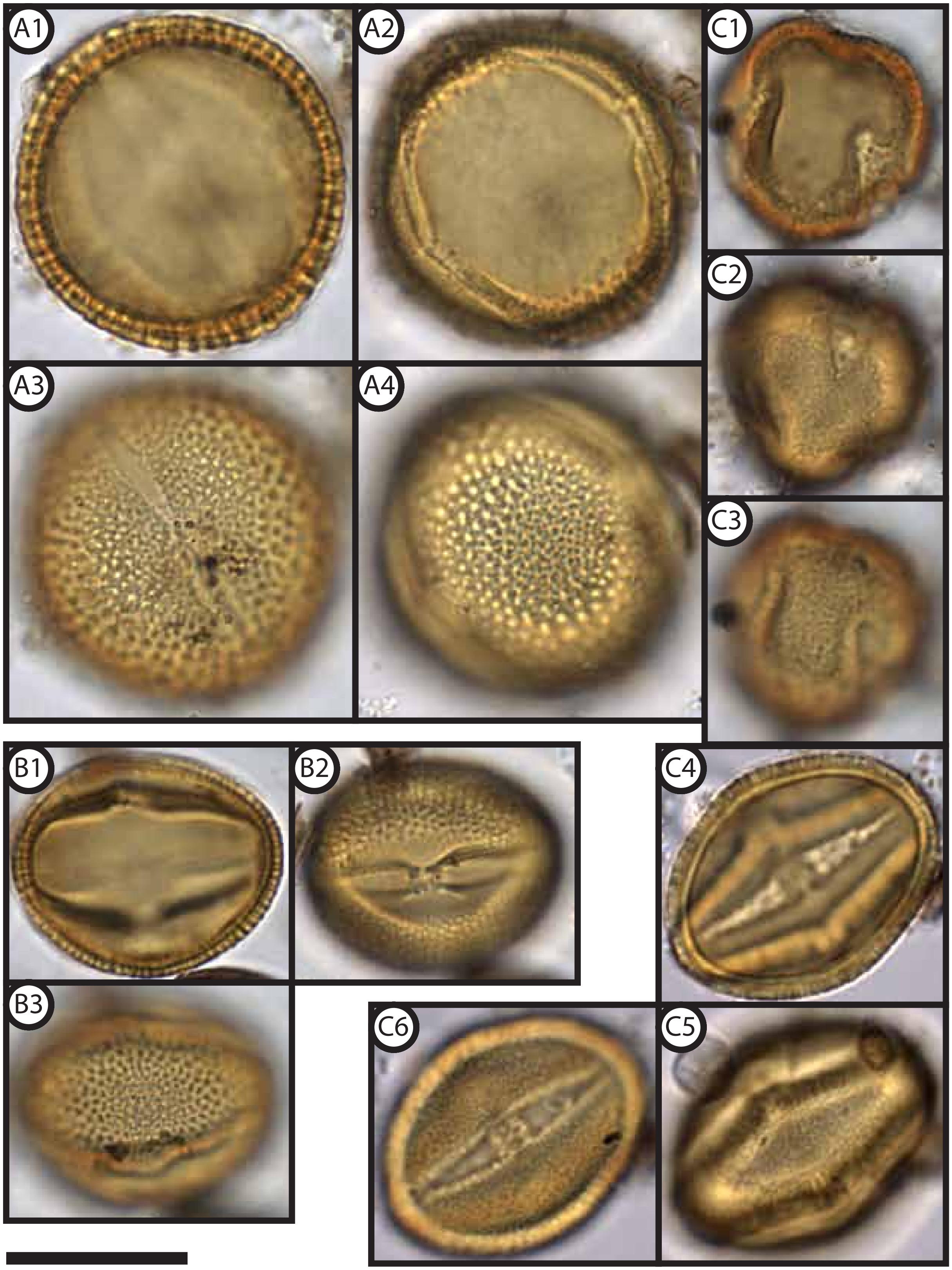

Plate S19. Euphorbiaceae: cf. *Alchornea* sp. (C1-C6); unknown sp.1 (B1-B3); unknown sp.2 (A1-A4)

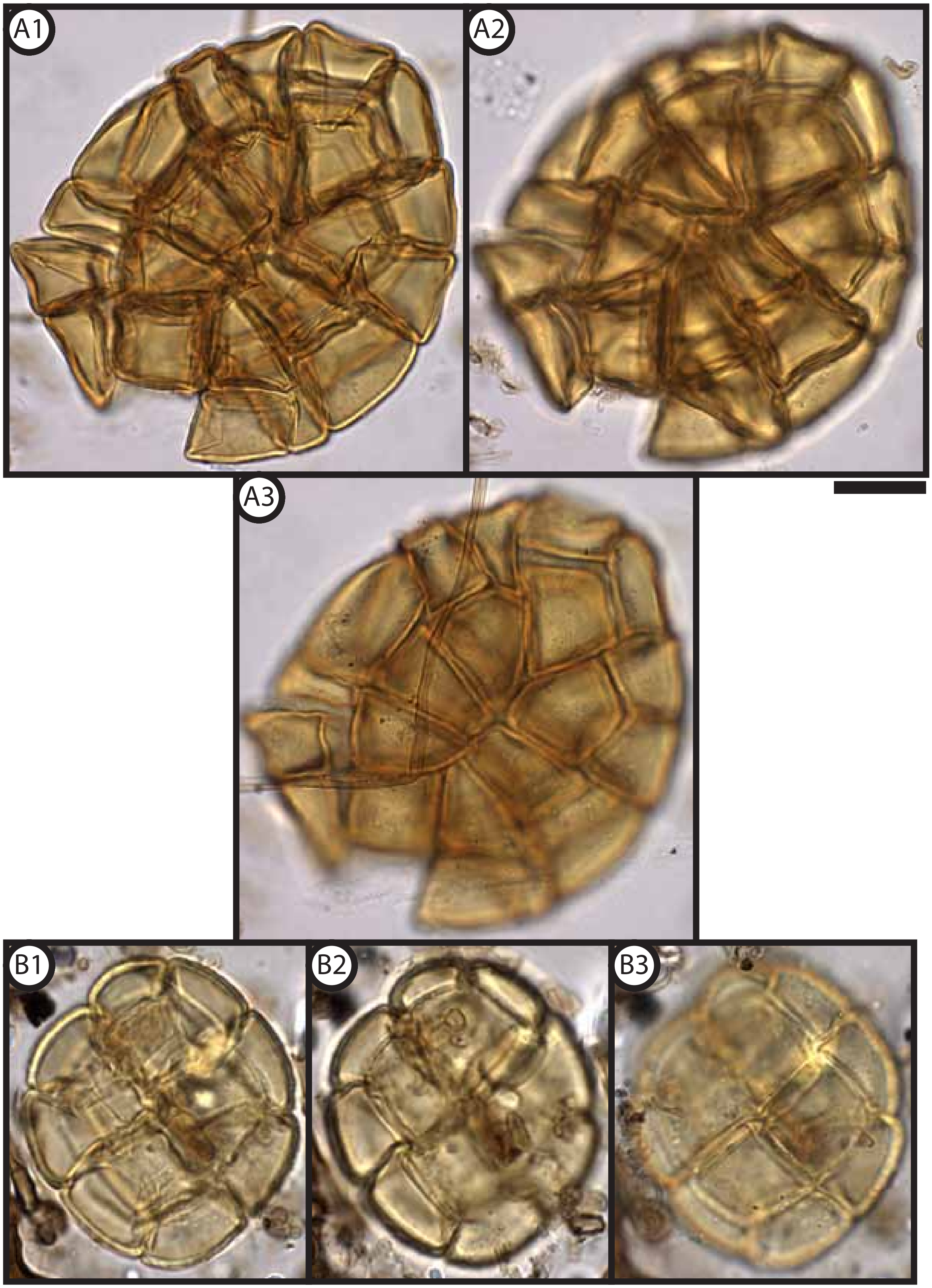

Plate S20. Fabaceae (Mimosoideae): cf. *Inga* sp. (A1-A3, image scaled 50%); unknown sp. 2 (B1-B3, image scaled 50%)

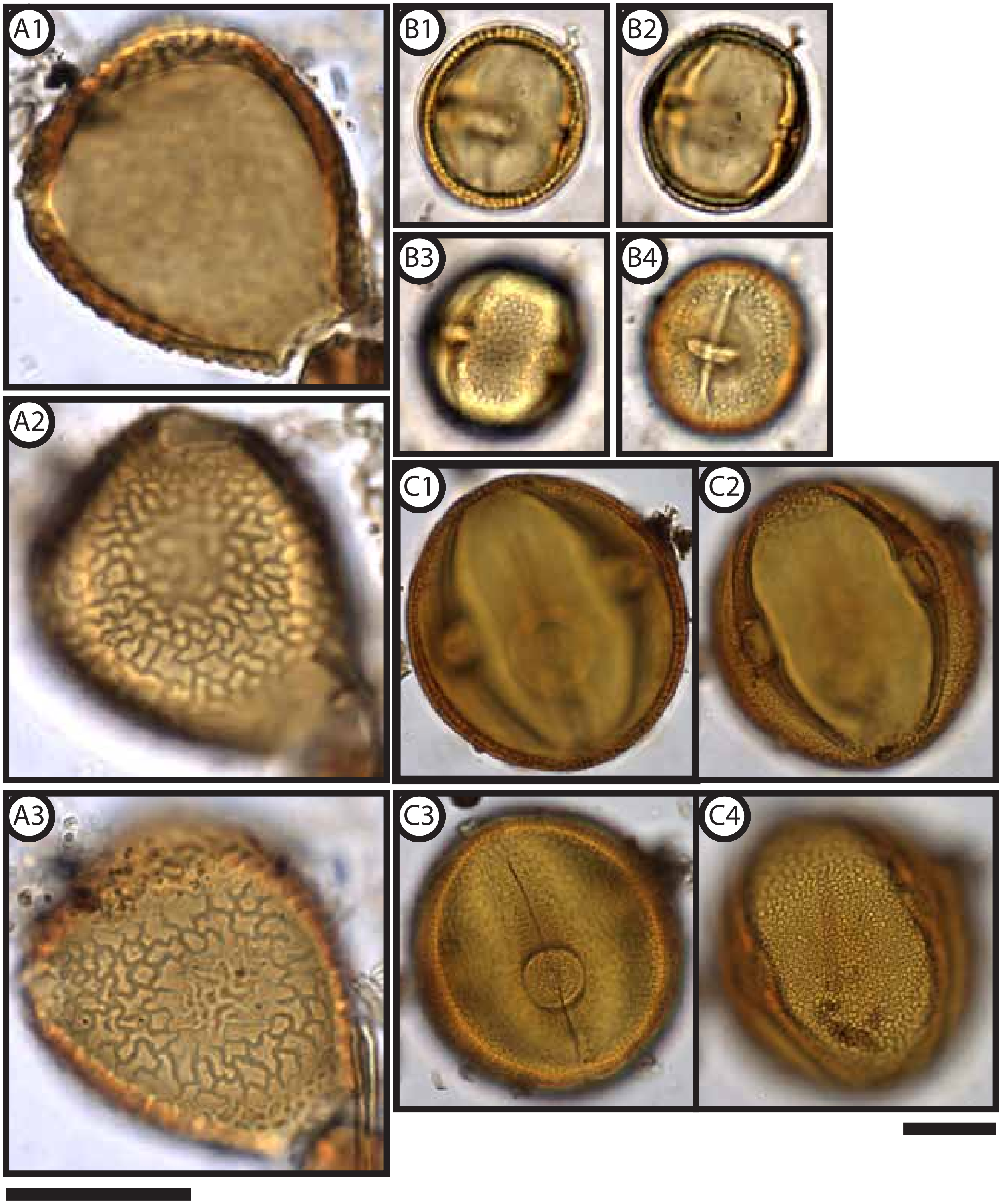

Plate S21. Fabaceae (Papilionoideae): *Erythrina costaricensis* (A1-A3); *Machaerium* sp. (B1-B4); Flacouticaceae/Salicaceae: cf. *Laetia procera* (C1-C4, image scaled 50%)

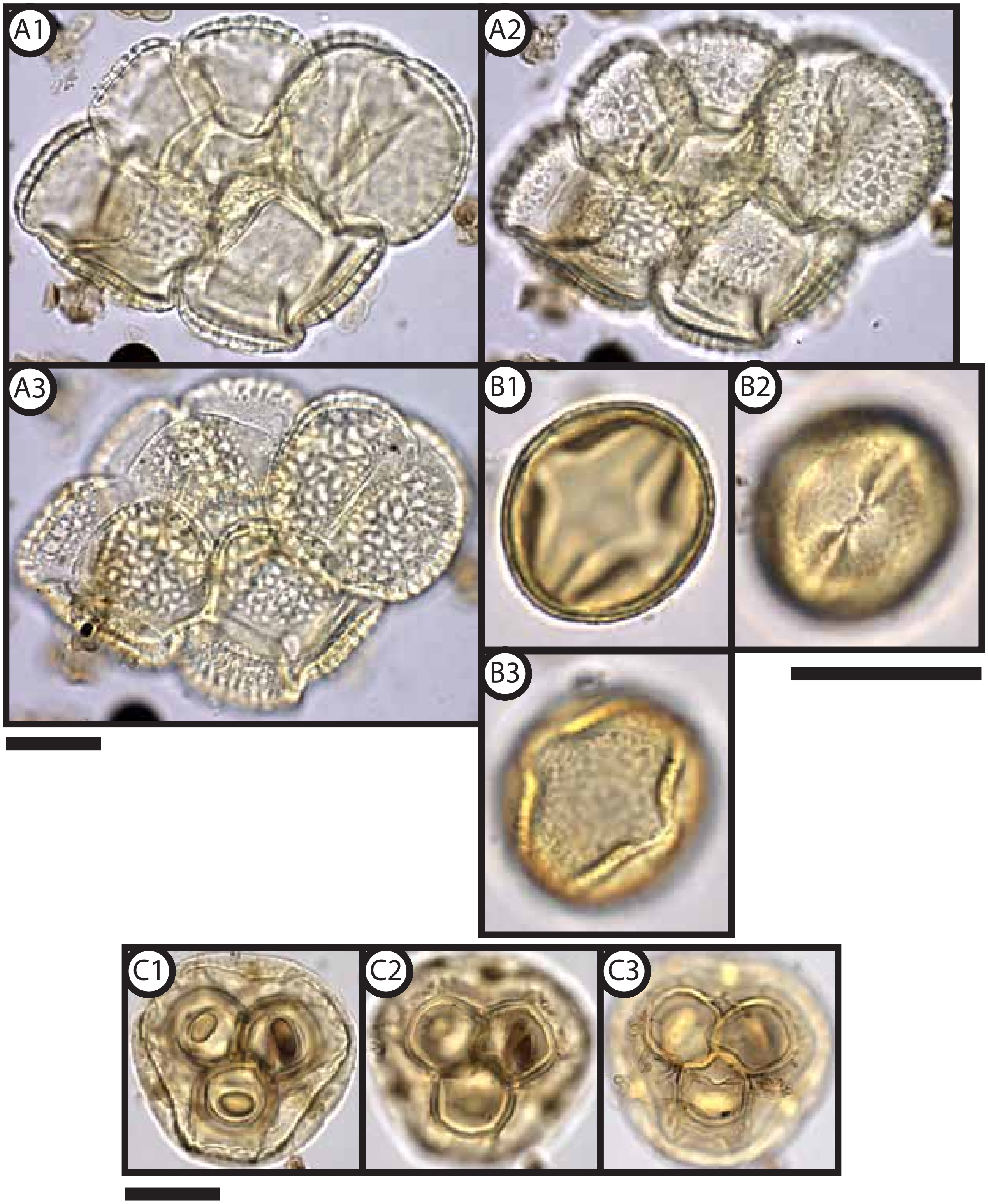

Plate S22. Hippocrataceae: *Hippocratea volubilis* (A1-A3, image scaled 50%); Lecythidaceae: cf. *Gustavia superba* (B1-B3); Loranthaceae: *Oryctanthus* sp. (C1-C3, image scaled 50%)
