# Supplementary material for: Variability within the 10-Year Pollen Rain of a Seasonal Neotropical Forest and Its Implications for Paleoenvironmental and Phenological Research
Source: PLoS One. 2013 Jan 8;8(1):e53485. doi: 10.1371/journal.pone.0053485 (PMC3540050; doi:10.1371/journal.pone.0053485)
Supplement: Appendix S3 — Plate images of named and unknown pollen types, Part II (Supporting Plates S23–S45). Scale bars represent 20 µm. Multiple images are provided to highlight the texture and cross-sectional shape of each grain. Images were taken using a Plan-Apochromat SF25 (63×, 1.4NA, oil immersion) lens and a Zeiss AxioCam ICc 3 digital microscope camera. (PDF) [file pone.0053485.s010.pdf]

SUPPORTING INFORMATION

HASELHORST, MORENO AND PUNYASENA

Variability within the 10-year pollen rain of a seasonal Neotropical forest and its implications for paleoenvironmental and phenological research

Appendix S3: Supporting Plates S23-S45. Plate images of named and unknown pollen type, Part II. Scale bars represent 20 µm. Multiple images are provided to highlight the texture and cross-sectional shape of each grain. Images were taken using a Plan-Apochromat SF25 (63x, 1.4NA, oil immersion) lens and a Zeiss AxioCam ICc 3 digital microscope camera.

Plate S23. Malpighiaceae: *Byrsonima* sp. (B1-B3); *Hiraea*-type (A1-A3); Malvaceae: cf. *Sida* sp. (C1-C3, image scaled 50%); Malvaceae (Bombacoideae): *Bombacopsis* sp. (D1-D3, image scaled 50%)

Plate S24. Malvaceae (Bombacoideae): *Cavanillesia* sp. (B1-B3, image scaled 50%); cf. *Ceiba/Ochroma* sp. (A1-A3, image scaled 50%)

Plate S25. Malvaceae (Bombacoideae): cf. *Ceiba/Ochroma* sp. (A1-A3, image scaled 50%); *Pseudobombax* sp. (B1-B3, image scaled 50%); *Quararibea asterolepis* (C1-C5, image scaled 50%)

Plate S26. Melastomataceae: *Miconia*-type (A1-A6); Meliaceae: *Cedrela* sp. (B1-B3); *Trichilia* sp. (C1-C3)

Plate S27. Meliaceae: *Trichilia* sp. (A1-A6); *Trichilia* sp. (B1-B6)

Plate S28. Moraceae/Urticaceae: *Brosimum*-type (A1-A3); *Ficus* sp. (B1-B3); Myristicaceae: Virola sp. (C1-C4)

Plate S29. Myrtaceae: *Eugenia coloradensis* (A1-A3); *Eugenia* sp. (B1-B3); *Eugenia* sp.2 (E1-E3); *Myrcia* sp. (C1-C3); Nyctaginaceae: *Guapira standleyana* (F1-F4, image scaled 50%); Piperaceae: *Piper* spp. (D1-D3)

Plate S30. Poaceae: unknown sp.1 (A1-A3); unknown sp.2 (B1-B3, image scaled 50%); Pinaceae/Podocarpaceae: unknown sp. (C1-C3, image scaled 50%); Proteaceae: cf. *Roupala montana* (D1-D3, image scaled 50%)

Plate S31. Rubiaceae: *Alseis* sp. (A1-A3); *Coussarea* sp. (B1-B3); *Faramea occidentalis* (C1-C4); *Faramea* sp. 2 (D1-D3); *Faramea* sp.3 (E1-E3)

Plate S32. Rubiaceae: *Genipa* sp. (A1-A6); cf. *Ixora coccinea* (B1-B6)

Plate S33. Rubiaceae: *Posoqueria* sp. (A1-A3, image scaled 50%); *Psychotria* sp.1 (B1-B3, image scaled 50%); *Psychotria* sp.2 (C1-C3, image scaled 50%); *Sabicea* sp. (E1-E3); *Tocoyena pittieri* (D1-D3, image scaled 50%)

Plate S34. Rubiaceae: *Uncaria tomentosa* (A1-A6); cf. *Warszewiczia* sp. (B1-B3); Rutaceae: *Citrus* sp.1 (C1-C4); *Citrus* sp.2 cf. *Citrus grandis* (D1-D3, image scaled 75%)

Plate S35. Rutaceae: *Zanthoxylum* sp.1 (A1-A6); *Zanthoxylum* sp.2 (B1-B3); cf. Rutaceae spp. (C1-C4)

Plate S36. Salicaceae: cf. *Casearia* sp. (A1-A6); Sapindaceae: *Paulinia* sp. (B1-B3, image scaled 75%); *Serajnia* sp. (C1-C3, image scaled 75%)

Plate S37. Sapotaceae: *Pouteria* sp. (A1-A6); Simaroubaceae: *Quassia* sp. (B1-B3, image scaled 75%); *Simarouba* sp. (C1-C3)

Plate S38. Solanaceae: *Solanum* spp. (A1-A7); Tiliaceae: cf. *Apeiba* sp. (B1-B3, image scaled 75%); Urticaceae: *Cecropia* sp. (C1-C3); Vitaceae: *Cissus* sp. (D1-D3, image scaled 75%)

Plate S39. Vochysiaceae: *Vochysia* sp. (A1-A3); unknown sp. (B1-B3)

Plate S40. unknown sp./cf. *Vismia* sp. (A1-A4); unknown sp./ cf. Rubiaceae spp. (B1-B3); unknown sp./cf. Ochnaceae: *Cespedezia* sp. (C1-C3)

Plate S41. unknown sp. (A1-A3); unknown sp. (B1-B3); unknown sp. (C1-C3)

Plate S42. unknown sp./cf. Malpighiaceae: *Tetrapteris* sp. (A1-A3); unknown sp. (B1-B6); unknown sp./cf. Rubiaceae spp. (C1-C4)

Plate S43. unknown sp. (A1-A4, image scaled 50%); unknown sp. (B1-B3, image scaled 50%); unknown sp. (C1-C3, scaled to 75%)

Plate S44. unknown sp. (A1-A4) unknown sp. (B1-B3); unknown sp./cf. Lamiaceae: *Hyptis* sp. (C1-C3)

Plate S45. unknown sp. (A1-A3); unknown sp./cf. Sterculiaceae: *Melochia* sp. (B1-B5)

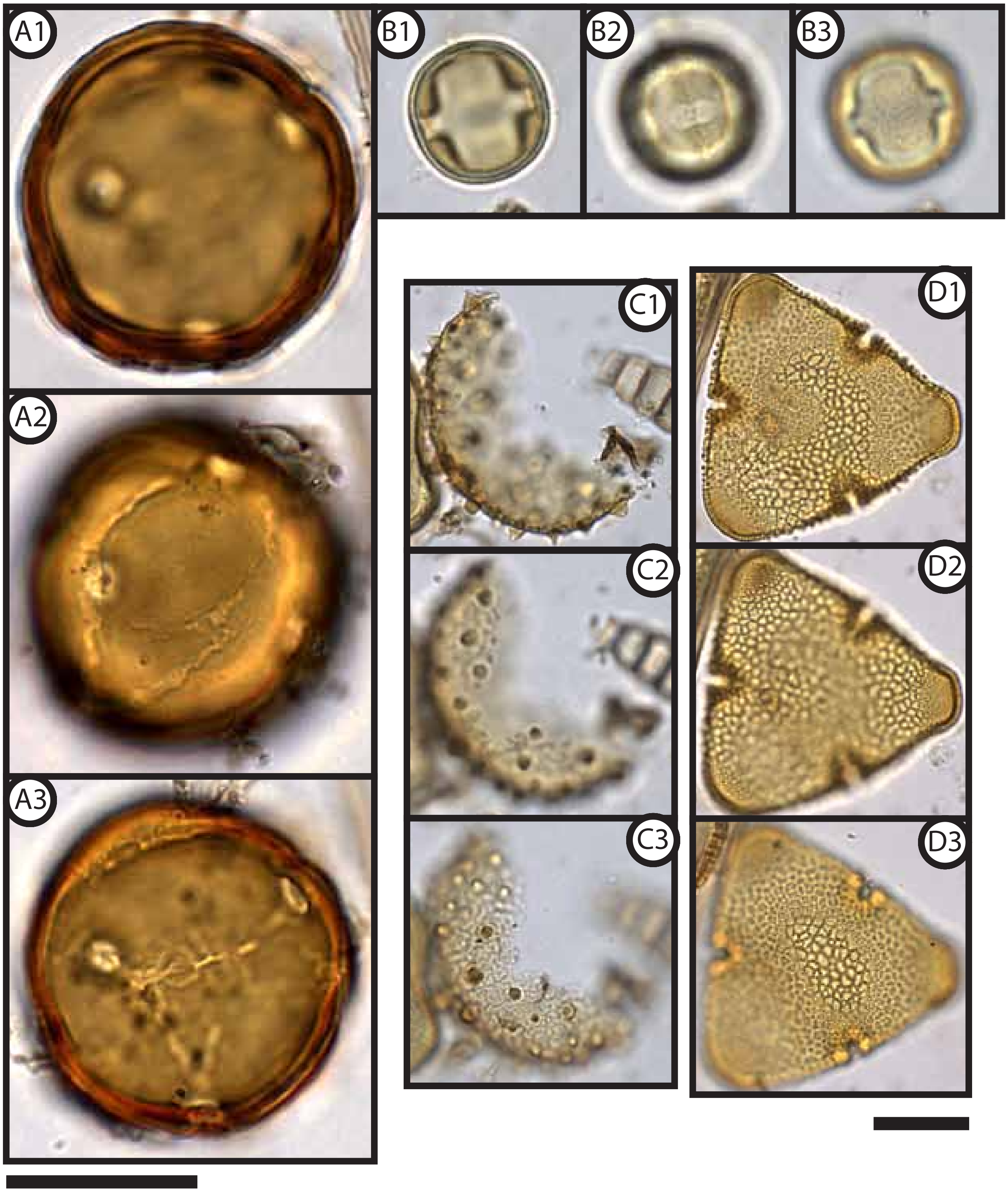

Plate S23. Malpighiaceae: *Byrsonima* sp. (B1-B3); *Hiraea*-type (A1-A3); Malvaceae: cf. *Sida* sp. (C1-C3, image scaled 50%); Malvaceae (Bombacoideae): *Bombacopsis* sp. (D1-D3, image scaled 50%)

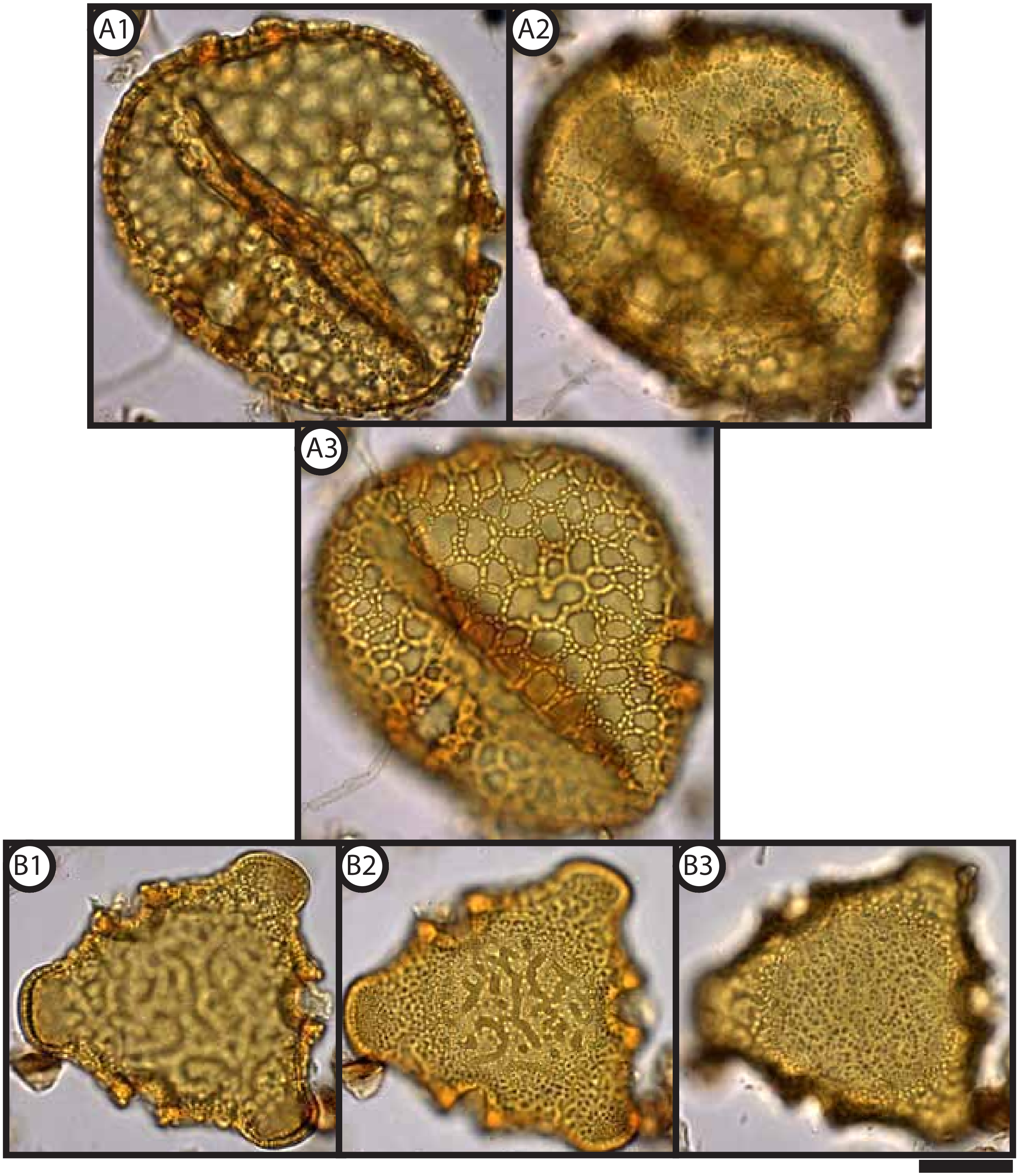

Plate S24. Malvaceae (Bombacoideae): *Cavanillesia* sp. (B1-B3, image scaled 50%); cf. *Ceiba/Ochroma* sp. (A1-A3, image scaled 50%)

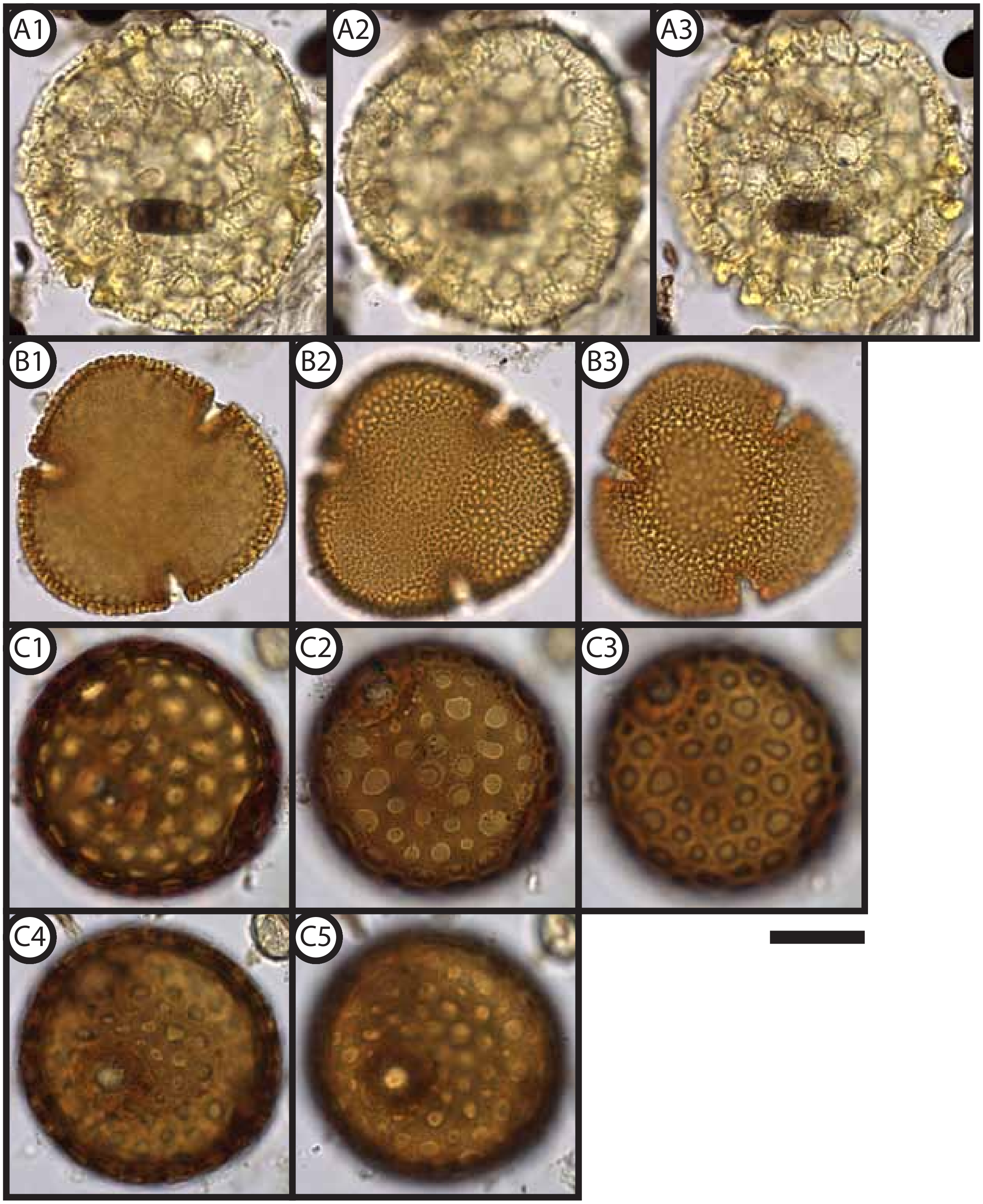

Plate S25. Malvaceae (Bombacoideae): cf. *Ceiba/Ochroma* sp. (A1-A3, image scaled 50%); *Pseudobombax* sp. (B1-B3, image scaled 50%); *Quararibea asterolepis* (C1-C5, image scaled 50%)

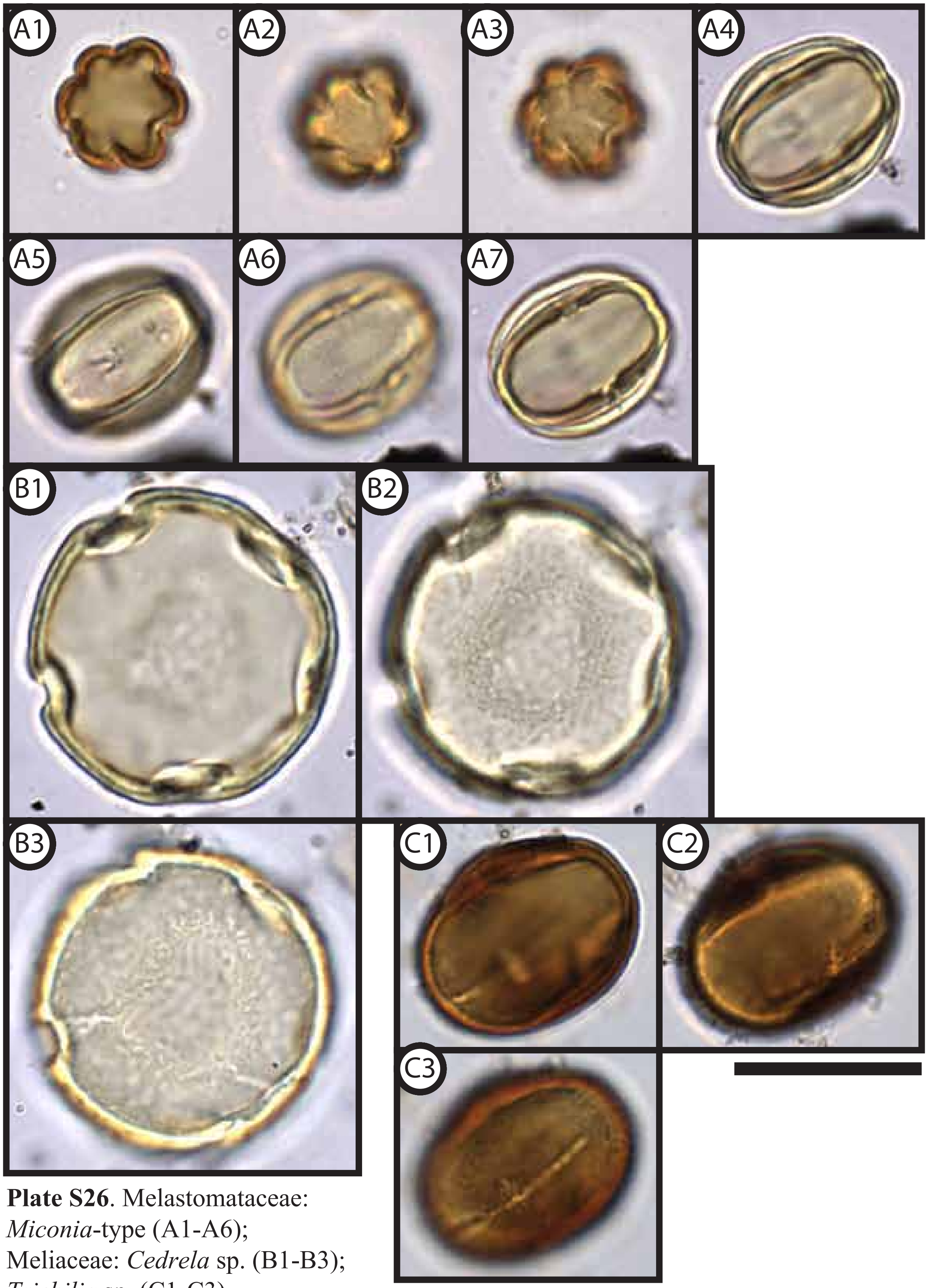

Plate S26. Melastomataceae: *Miconia*-type (A1-A6); Meliaceae: *Cedrela* sp. (B1-B3); *Trichilia* sp. (C1-C3)

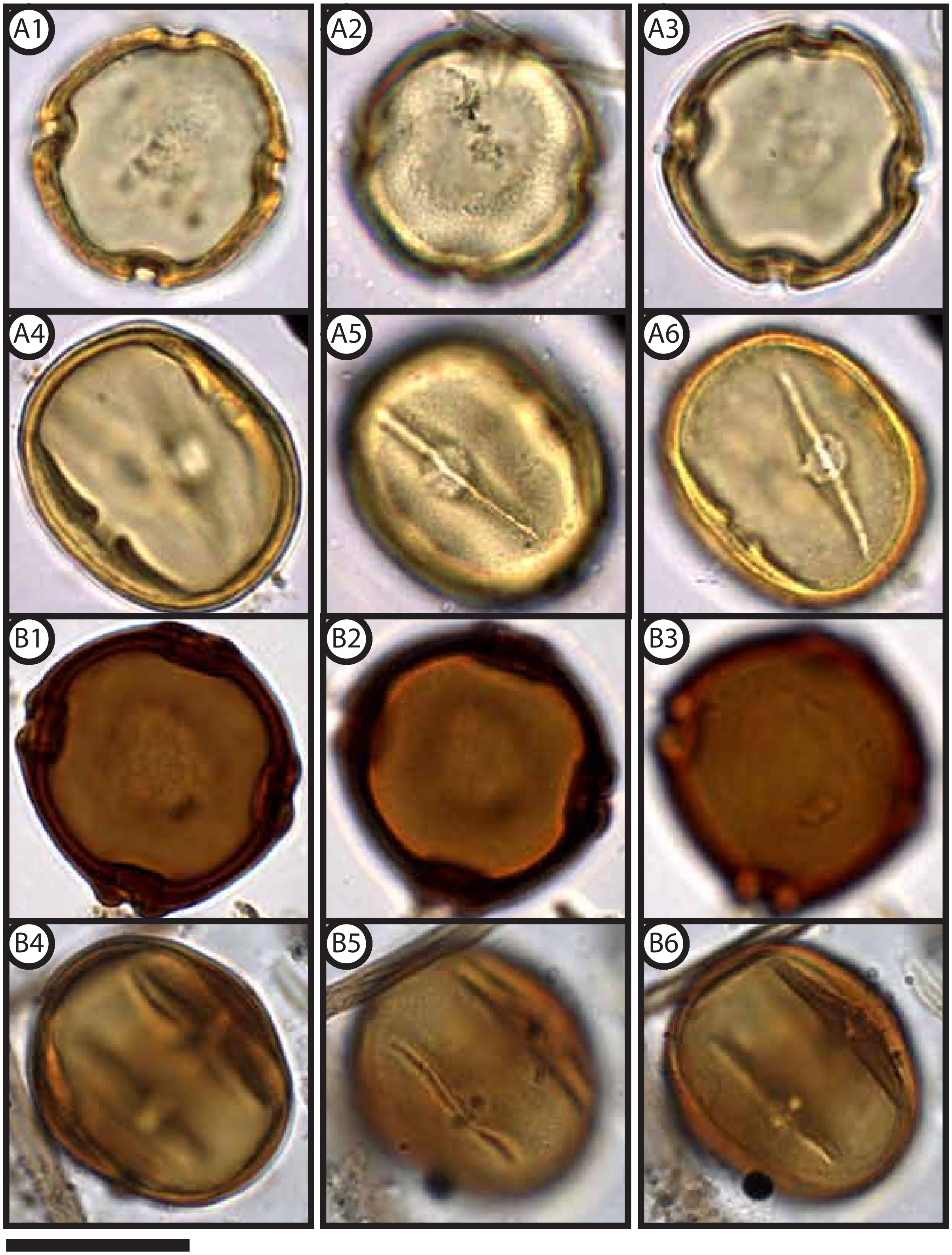

Plate S27. Meliaceae: *Trichilia* sp. (A1-A6); *Trichilia* sp. (B1-B6)

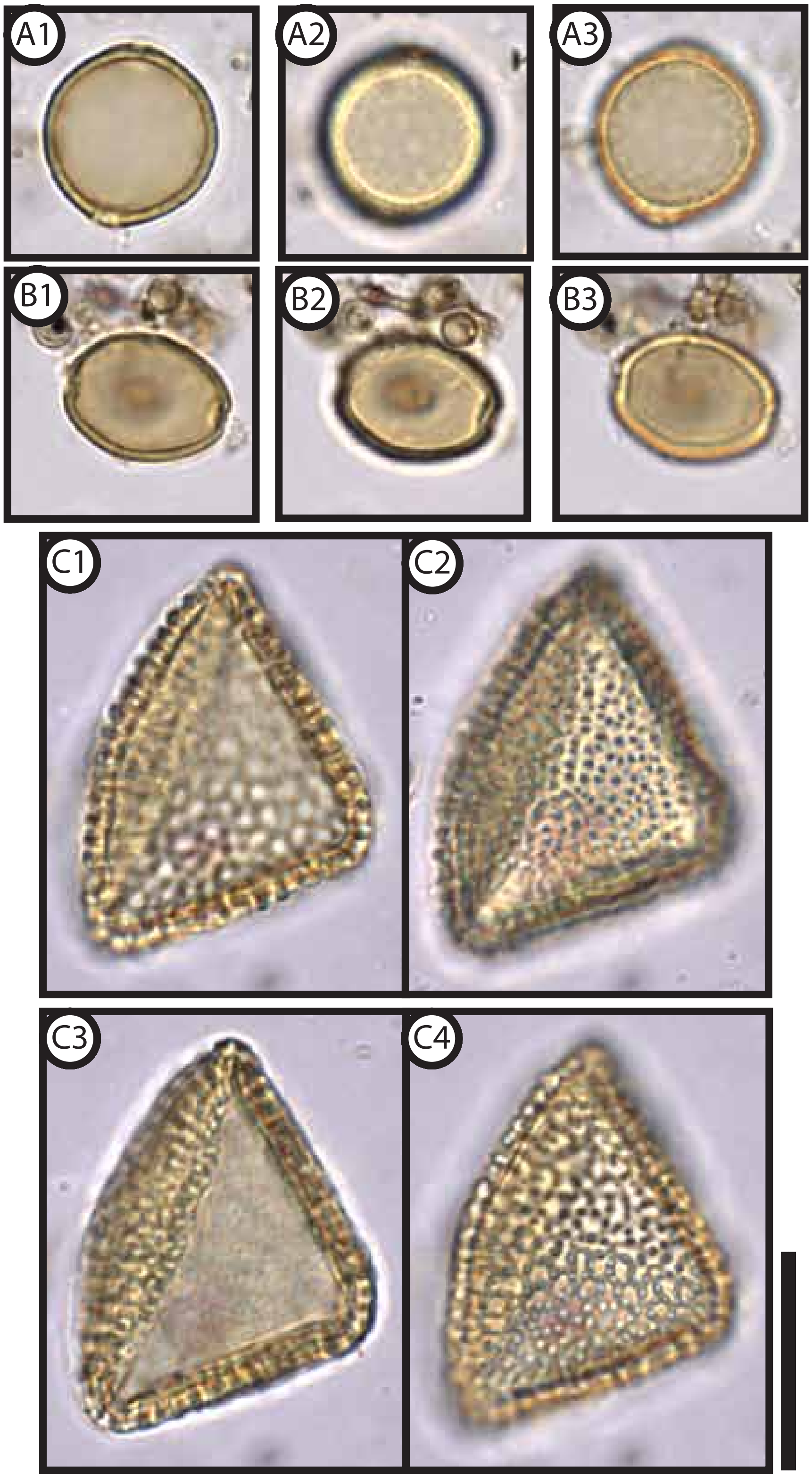

Plate S28. Moraceae/Urticaceae: *Brosimum*-type (A1-A3); *Ficus* sp. (B1-B3); Myristicaceae: *Virola* sp. (C1-C4)

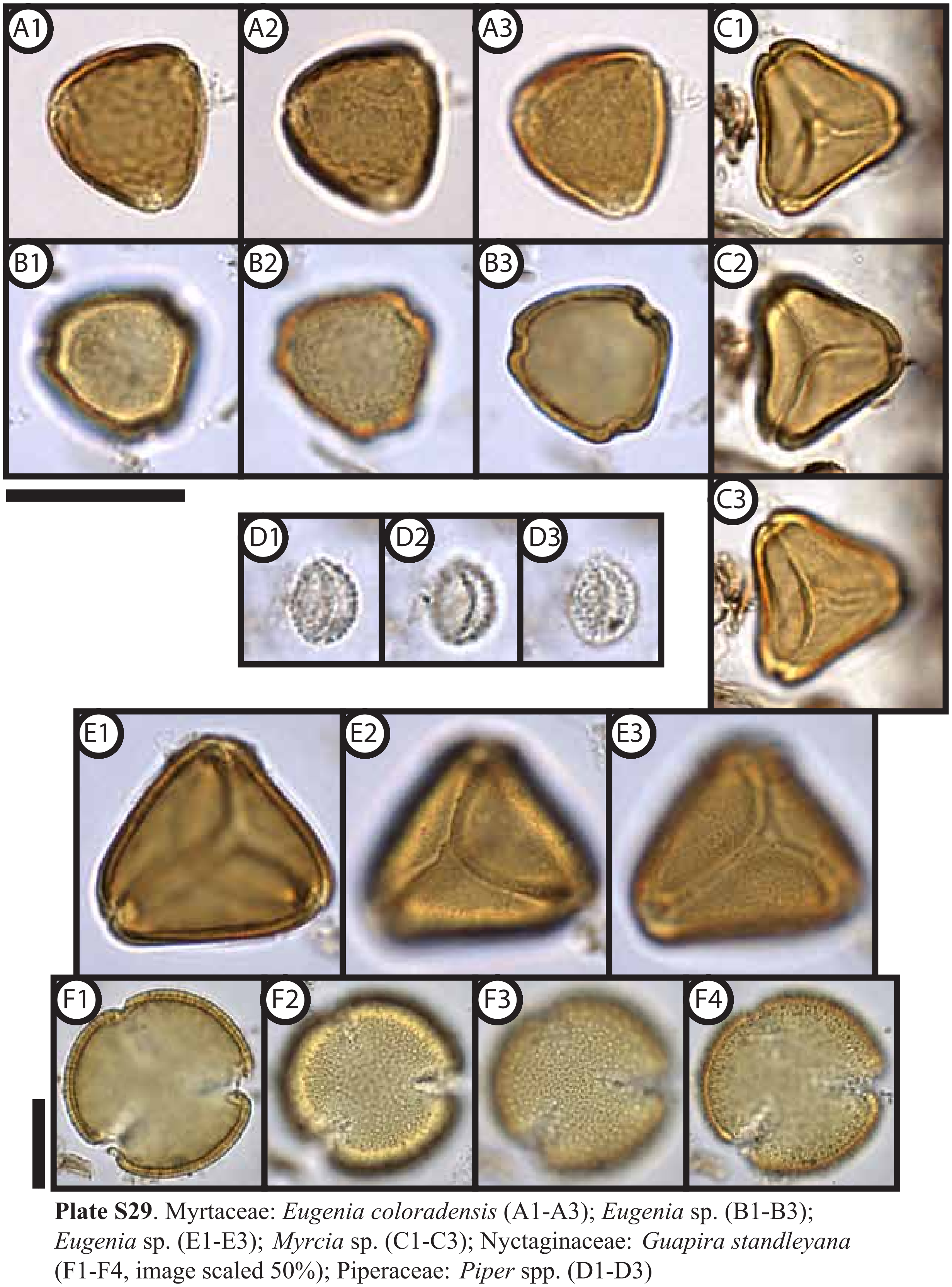

Plate S29. Myrtaceae: *Eugenia coloradensis* (A1-A3); *Eugenia* sp. (B1-B3); *Eugenia* sp. (E1-E3); *Myrcia* sp. (C1-C3); Nyctaginaceae: *Guapira standleyana* (F1-F4, image scaled 50%); Piperaceae: *Piper* spp. (D1-D3)

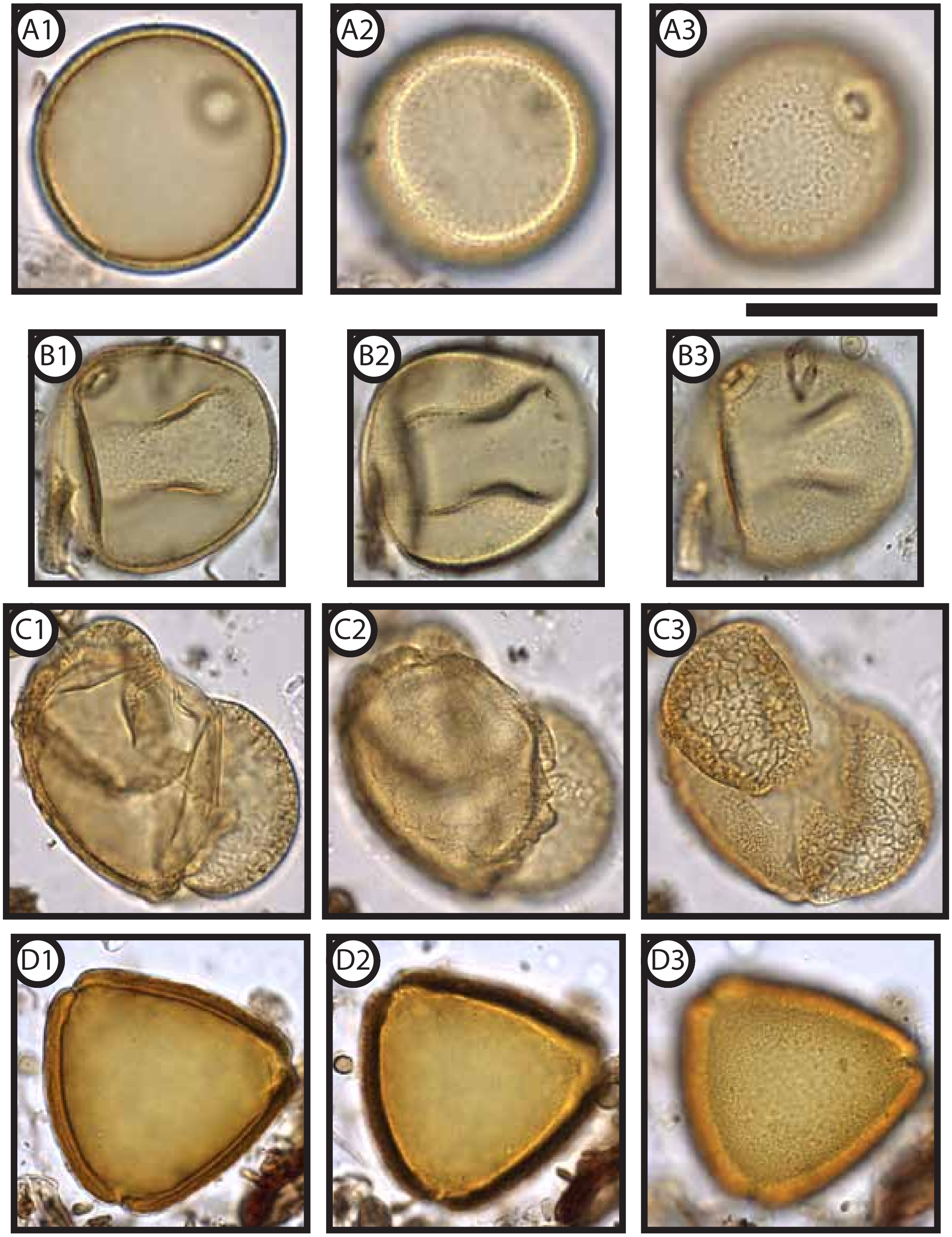

Plate S30. Poaceae: unknown sp.1 (A1-A3); unknown sp.2 (B1-B3, image scaled 50%); Pinaceae/Podocarpaceae: unknown sp. (C1-C3, image scaled 50%); Proteaceae: cf. *Roupala montana* (D1-D3, image scaled 50%)

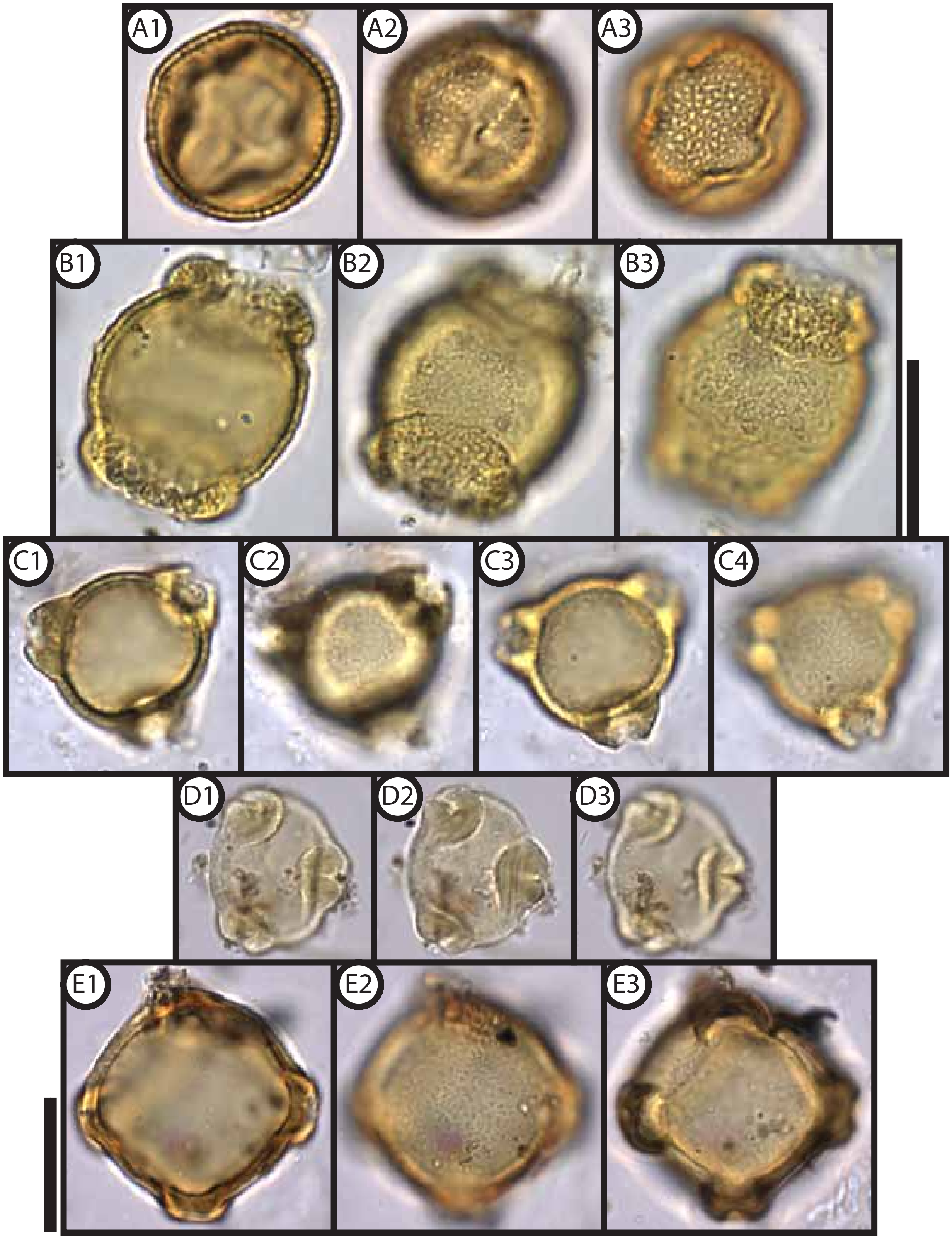

Plate S31. Rubiaceae: *Alseis* sp. (A1-A3); *Coussarea* sp. (B1-B3); *Faramea occidentalis* (C1-C4); *Faramea* sp. 2 (D1-D3); *Faramea* sp.3 (E1-E3)

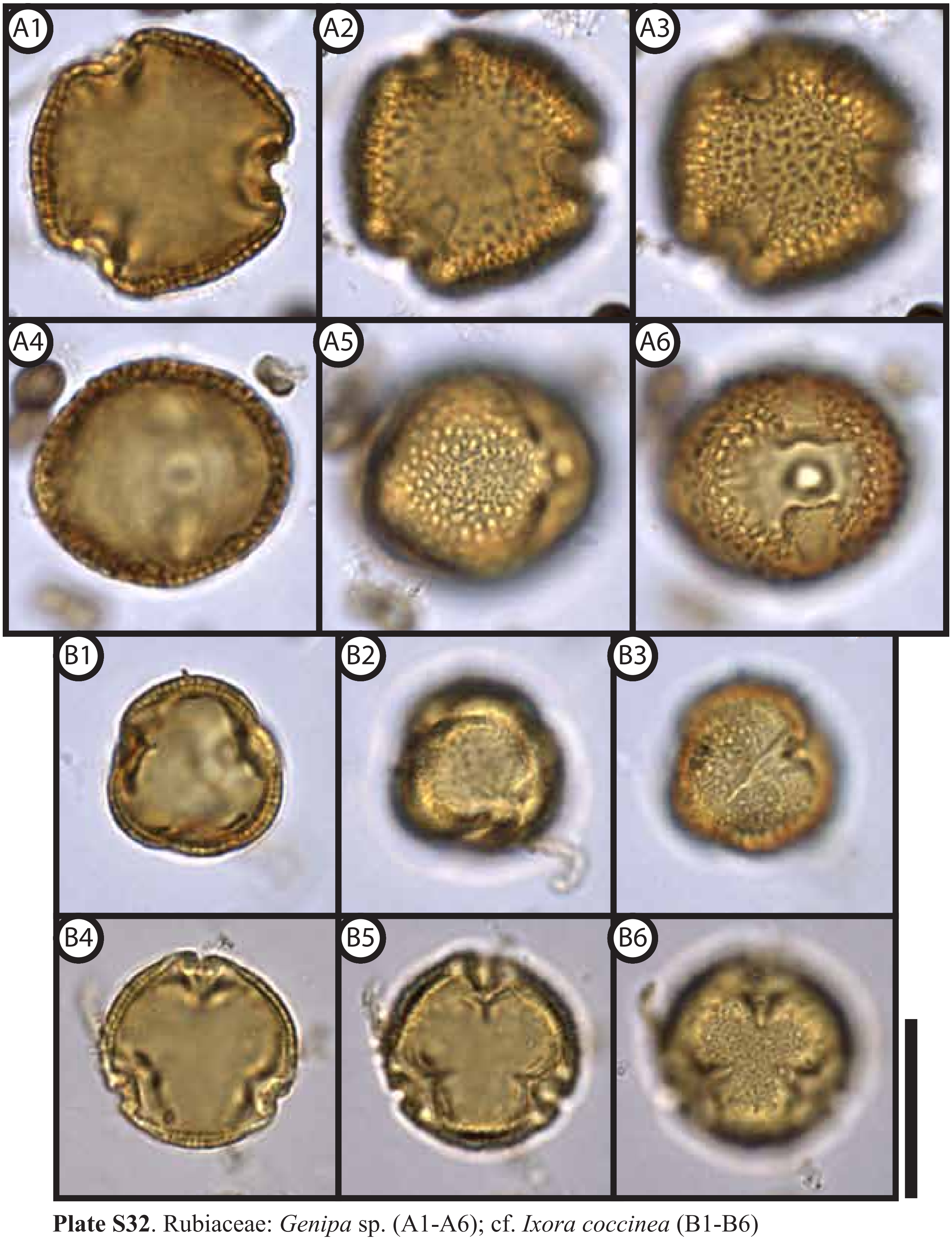

Plate S32. Rubiaceae: *Genipa* sp. (A1-A6); cf. *Ixora coccinea* (B1-B6)

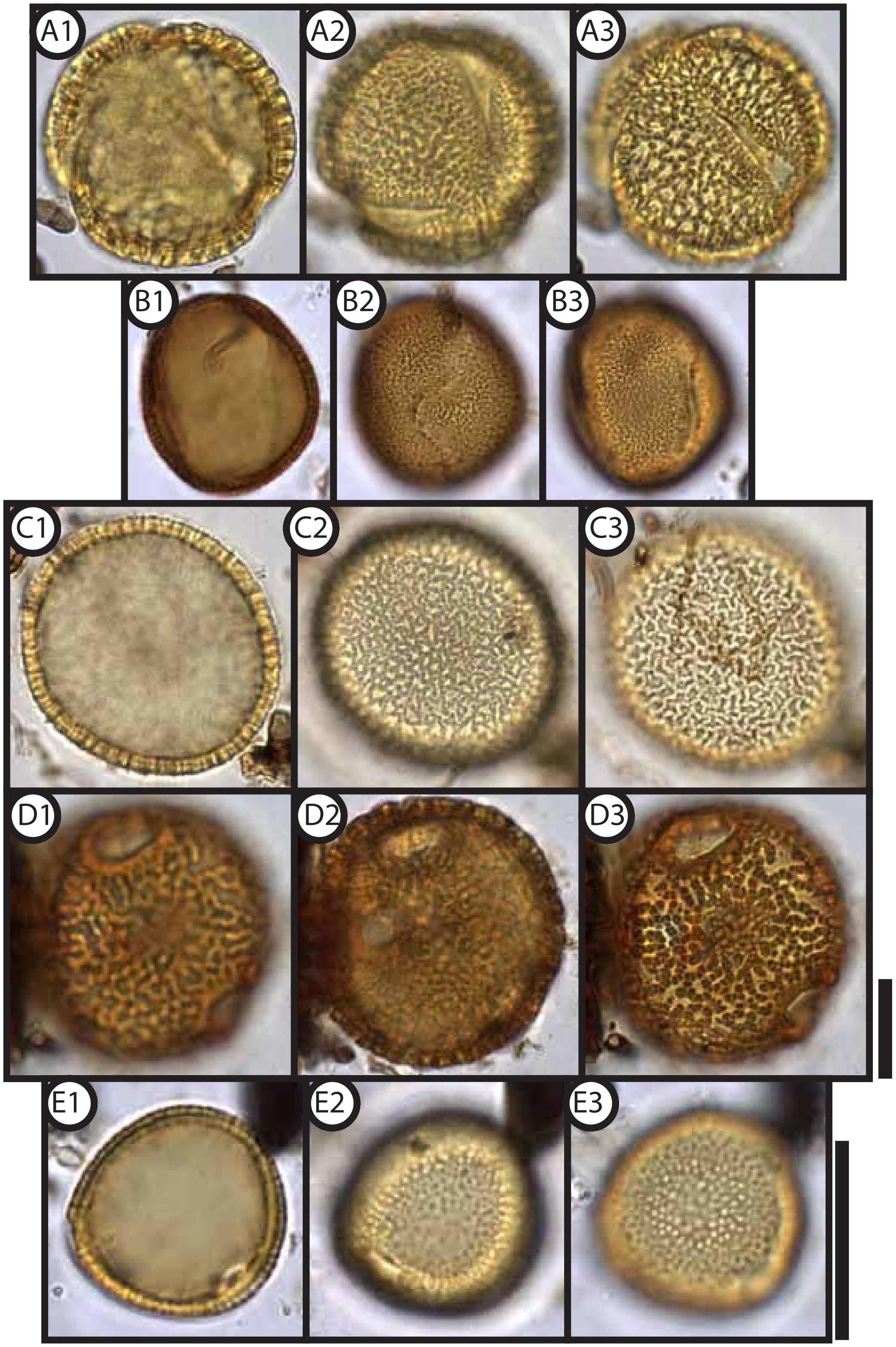

Plate S33. Rubiaceae: *Posoqueria* sp. (A1-A3, image scaled 50%); *Psychotria* sp.1 (B1-B3, image scaled 50%); *Psychotria* sp.2 (C1-C3, image scaled 50%); *Sabicea* sp. (E1-E3); *Tocoyena pittieri* (D1-D3, image scaled 50%)

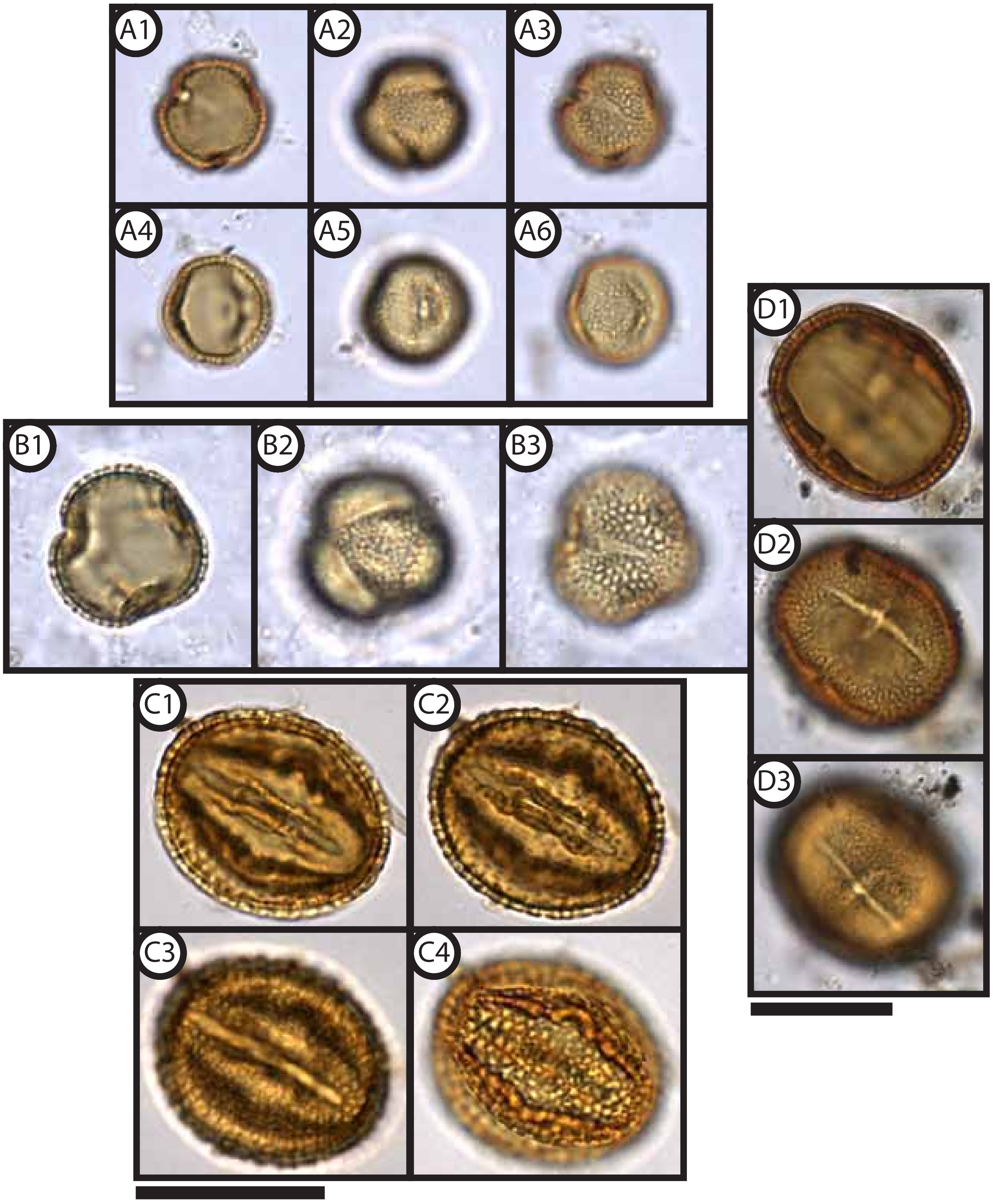

Plate S34. Rubiaceae: *Uncaria tomentosa* (A1-A6); cf. *Warszewiczia* sp. (B1-B3); Rutaceae: *Citrus* sp.1 (C1-C4); *Citrus* sp.2 cf. *grandis* (D1-D3, image scaled 75%)

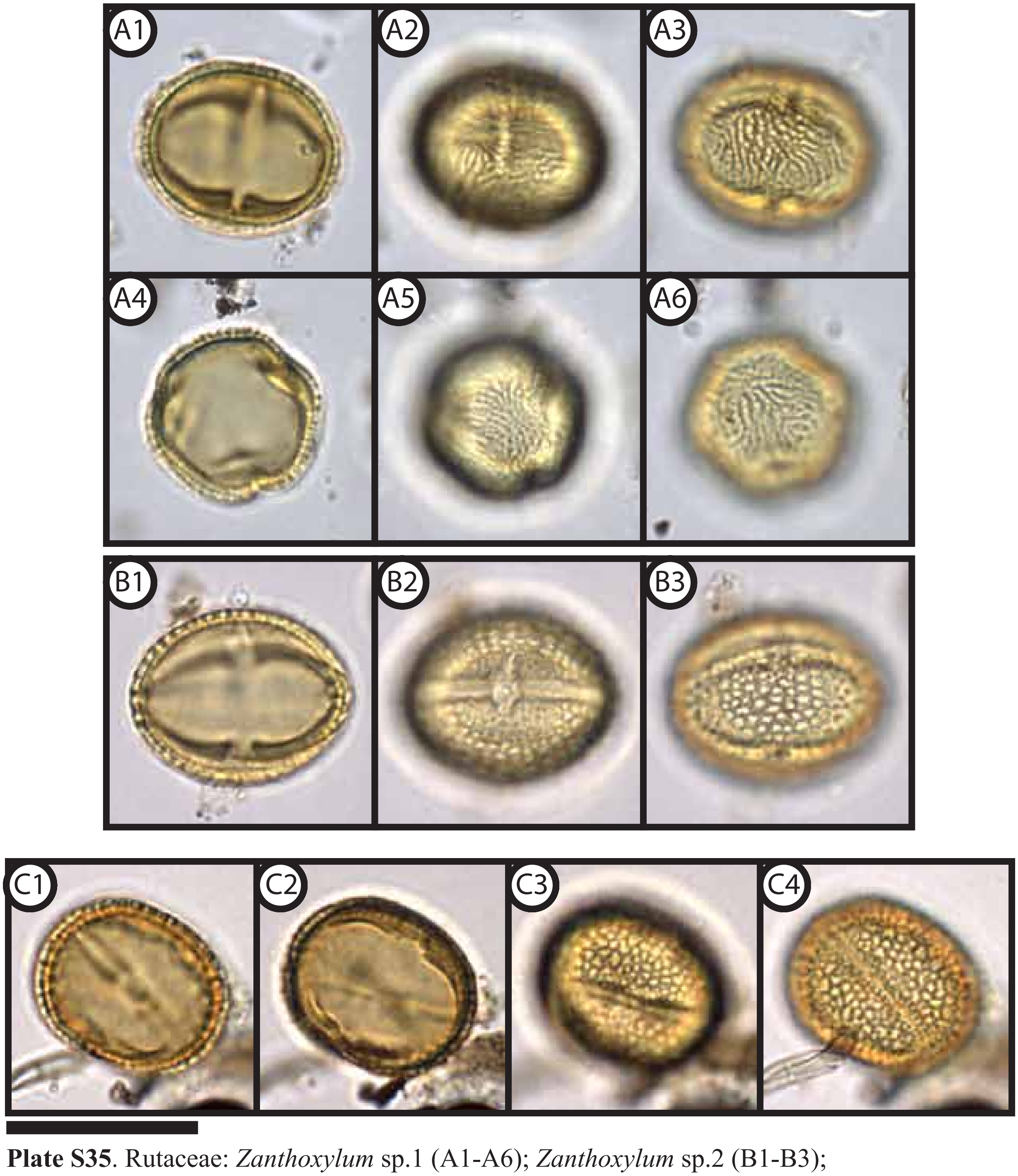

Plate S35. Rutaceae: *Zanthoxylum* sp.1 (A1-A6); *Zanthoxylum* sp.2 (B1-B3); cf. Rutaceae spp. (C1-C4)

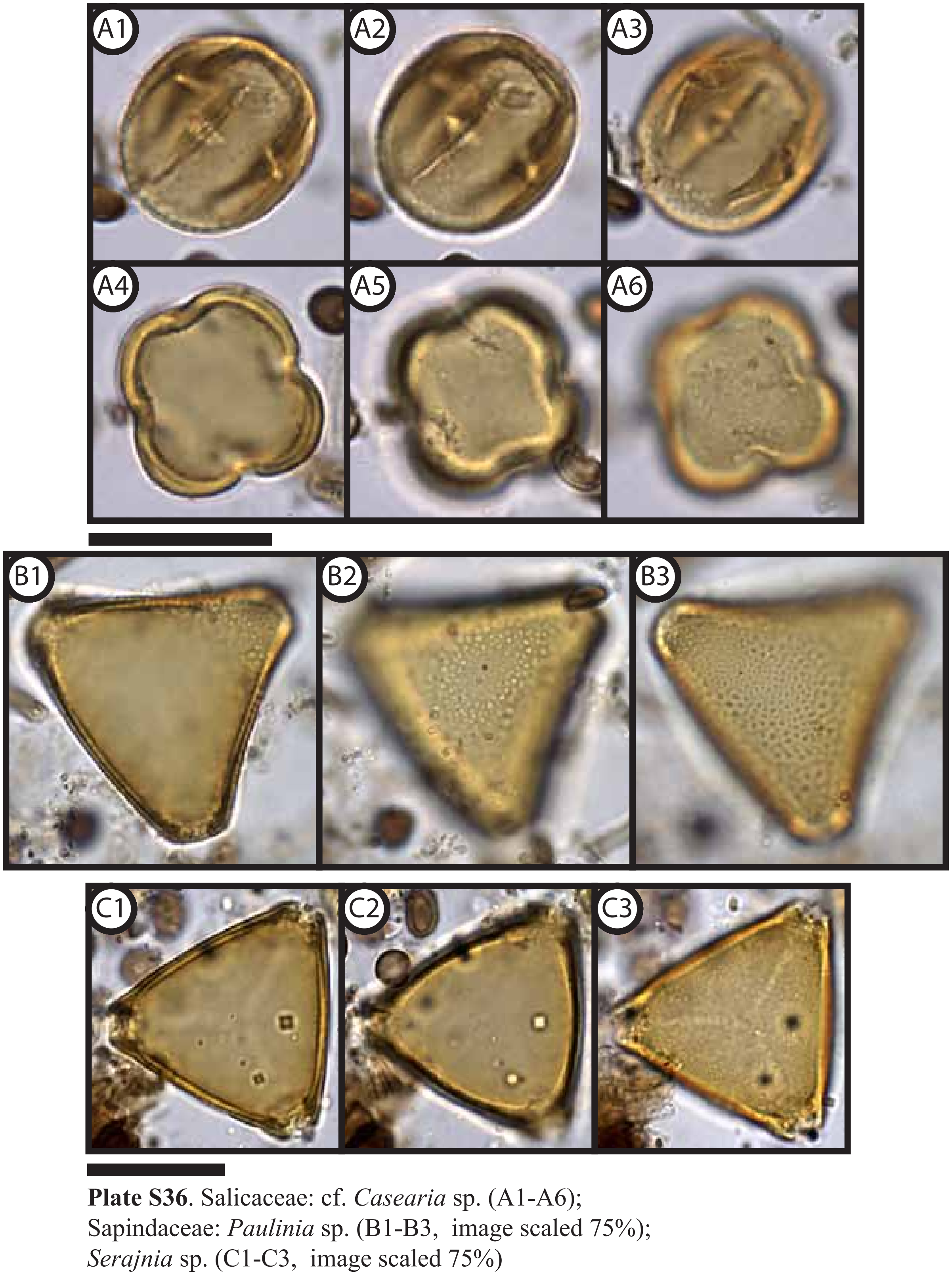

Plate S36. Salicaceae: cf. *Casearia* sp. (A1-A6);
Sapindaceae: *Paulinia* sp. (B1-B3, image scaled 75%);
Serajnia sp. (C1-C3, image scaled 75%)

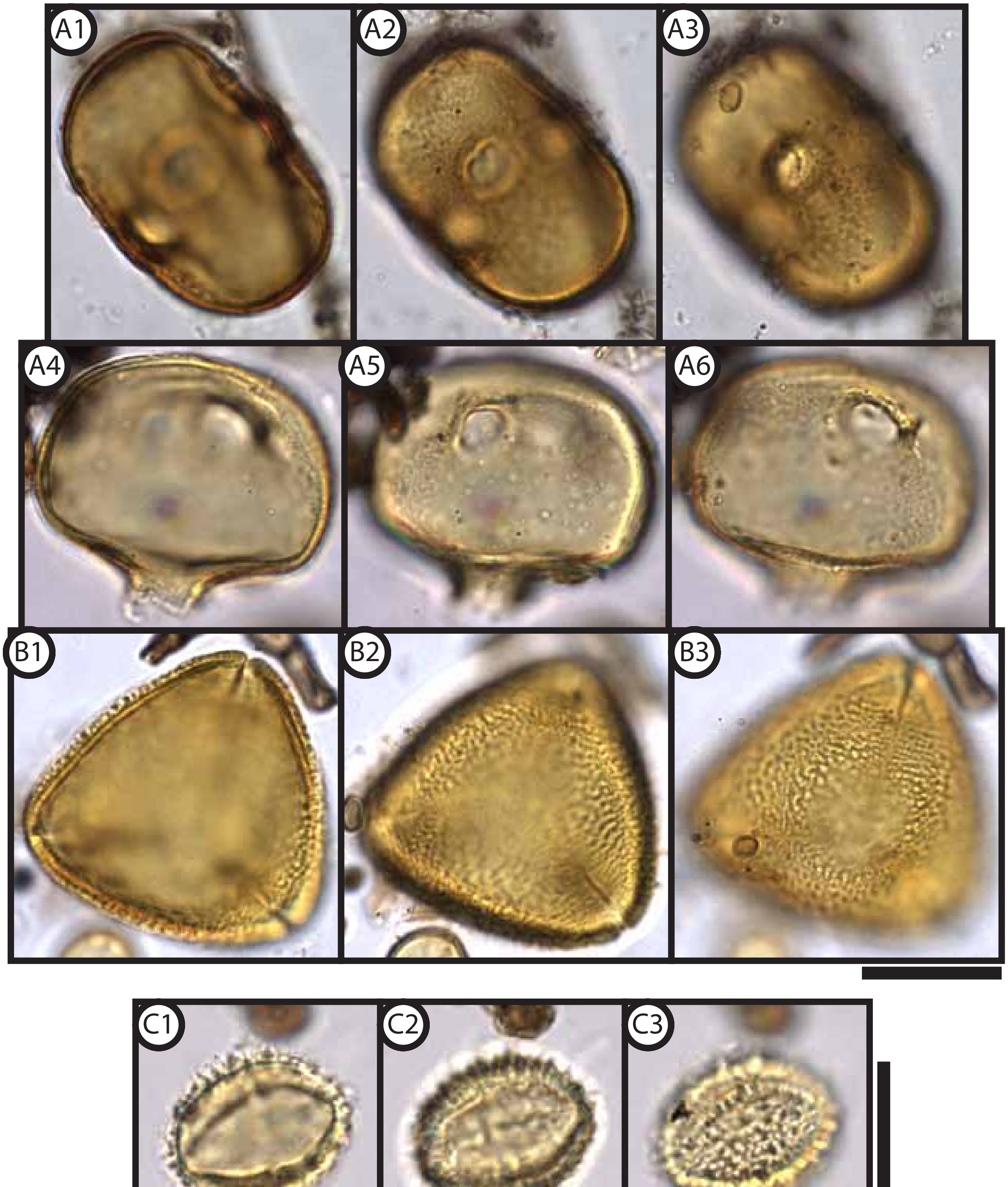

Plate S37. Sapotaceae: *Pouteria* sp. (A1-A6); Simaroubaceae: *Quassia* sp. (B1-B3, image scaled 75%); *Simarouba* sp. (C1-C3)

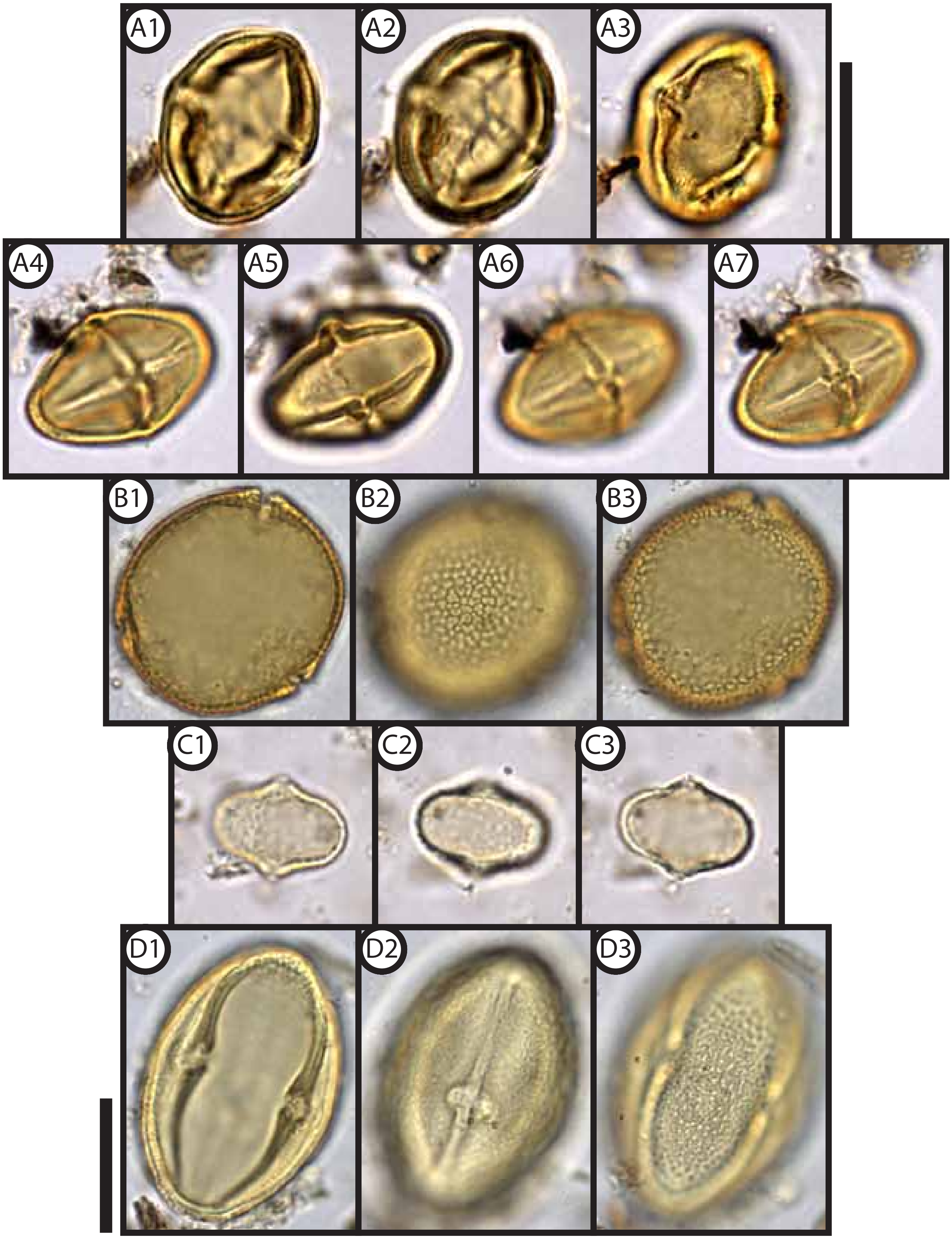

Plate S38. Solanaceae: *Solanum* spp. (A1-A7); Tiliaceae: cf. *Apeiba* sp. (B1-B3, image scaled 75%); Urticaceae: *Cecropia* sp. (C1-C3); Vitaceae: *Cissus* sp. (D1-D3, image scaled 75%)

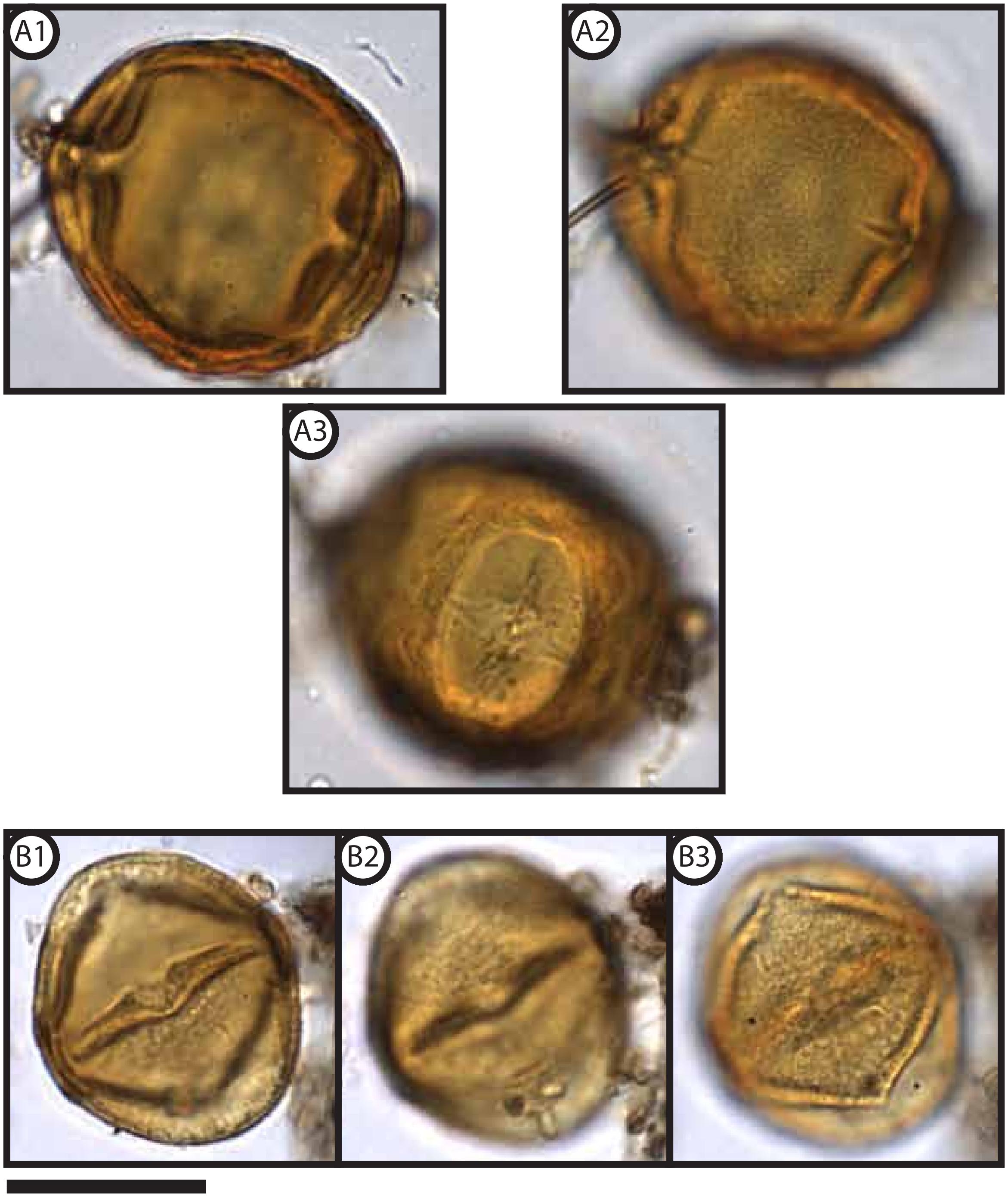

Plate S39. Vochysiaceae: *Vochysia* sp. (A1-A3); unknown sp. (B1-B3)

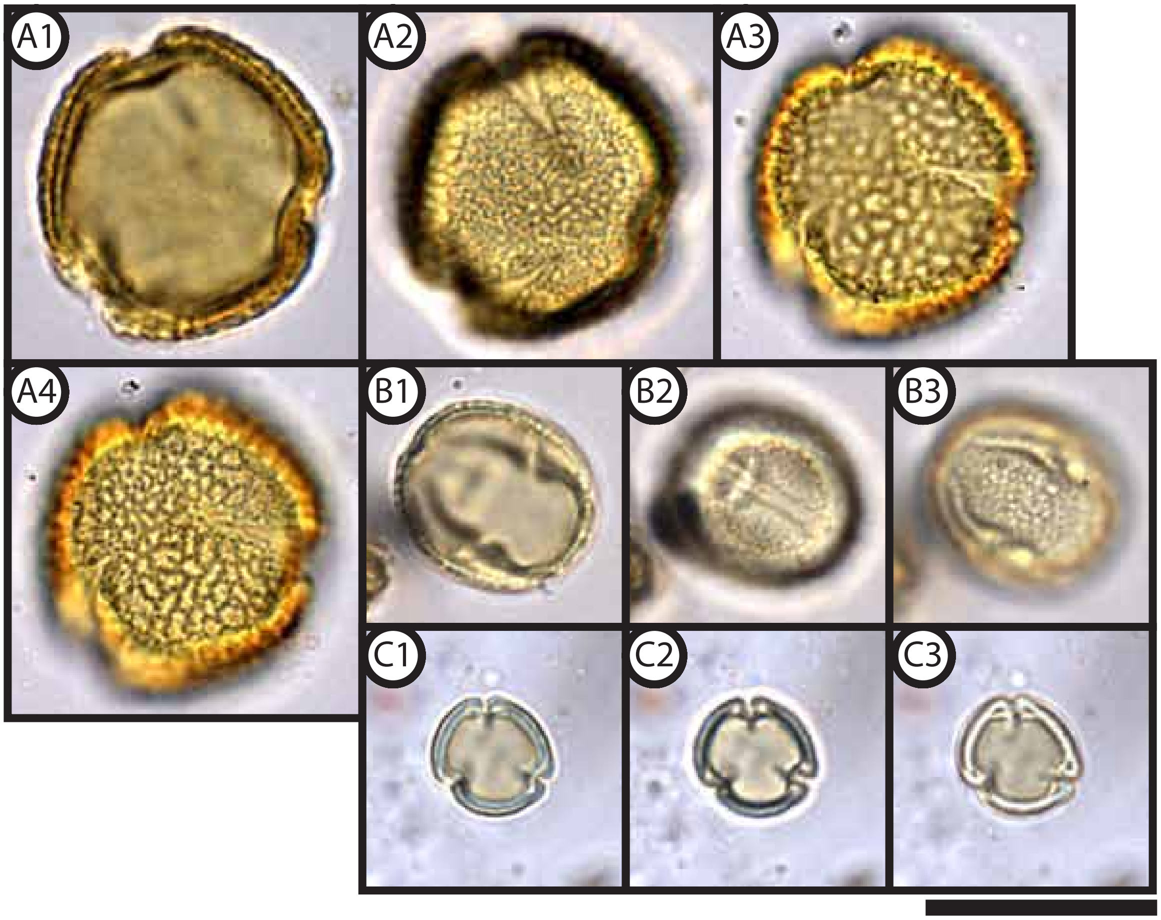

Plate S40. unknown sp./cf. *Vismia* sp. (A1-A4); unknown sp./cf. Rubiaceae spp. (B1-B3); unknown sp./cf. Ochnaceae: *Cespedezia* sp. (C1-C3)

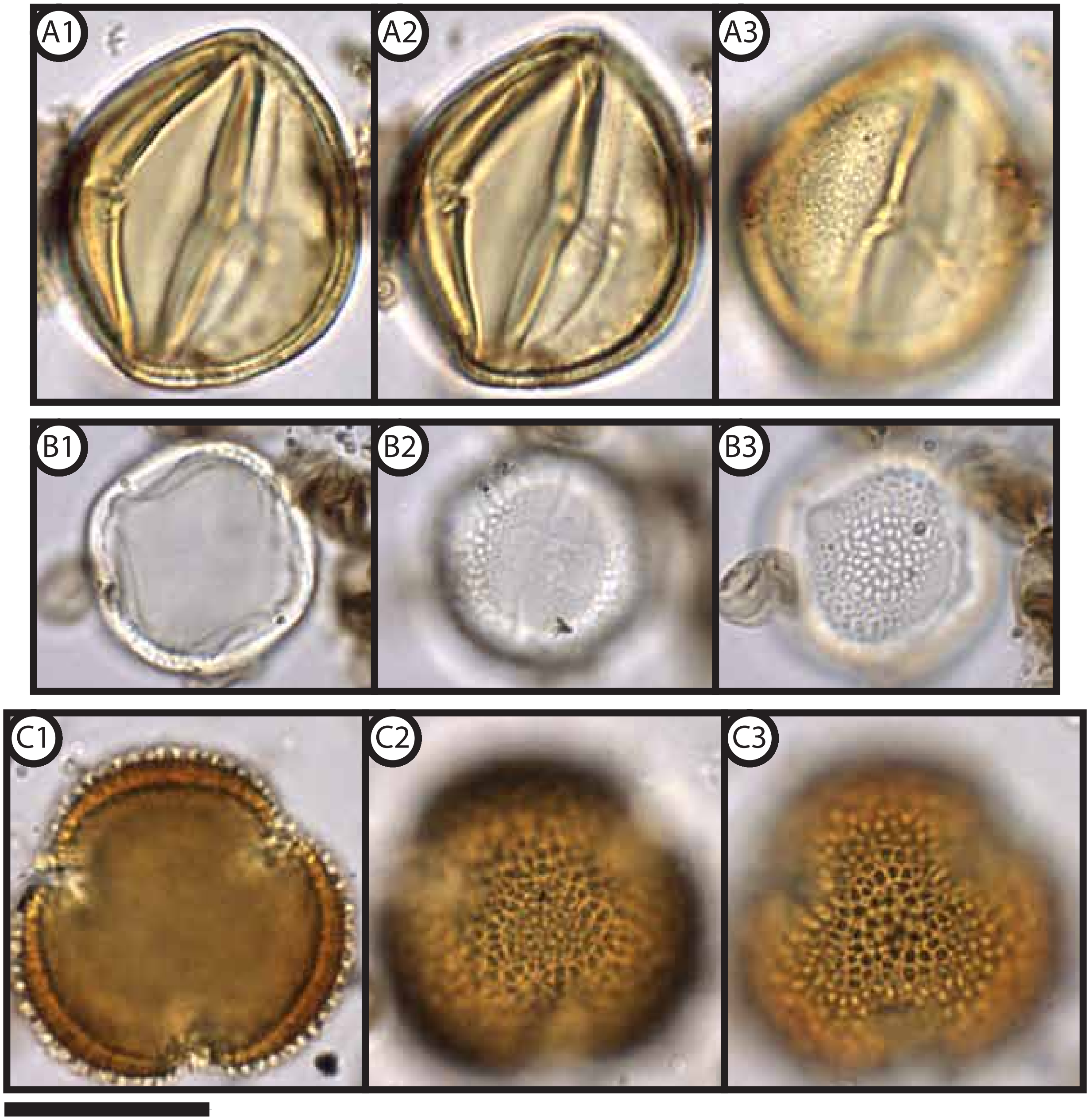

Plate S41. unknown sp. (A1-A3); unknown sp. (B1-B3); unknown sp. (C1-C3)

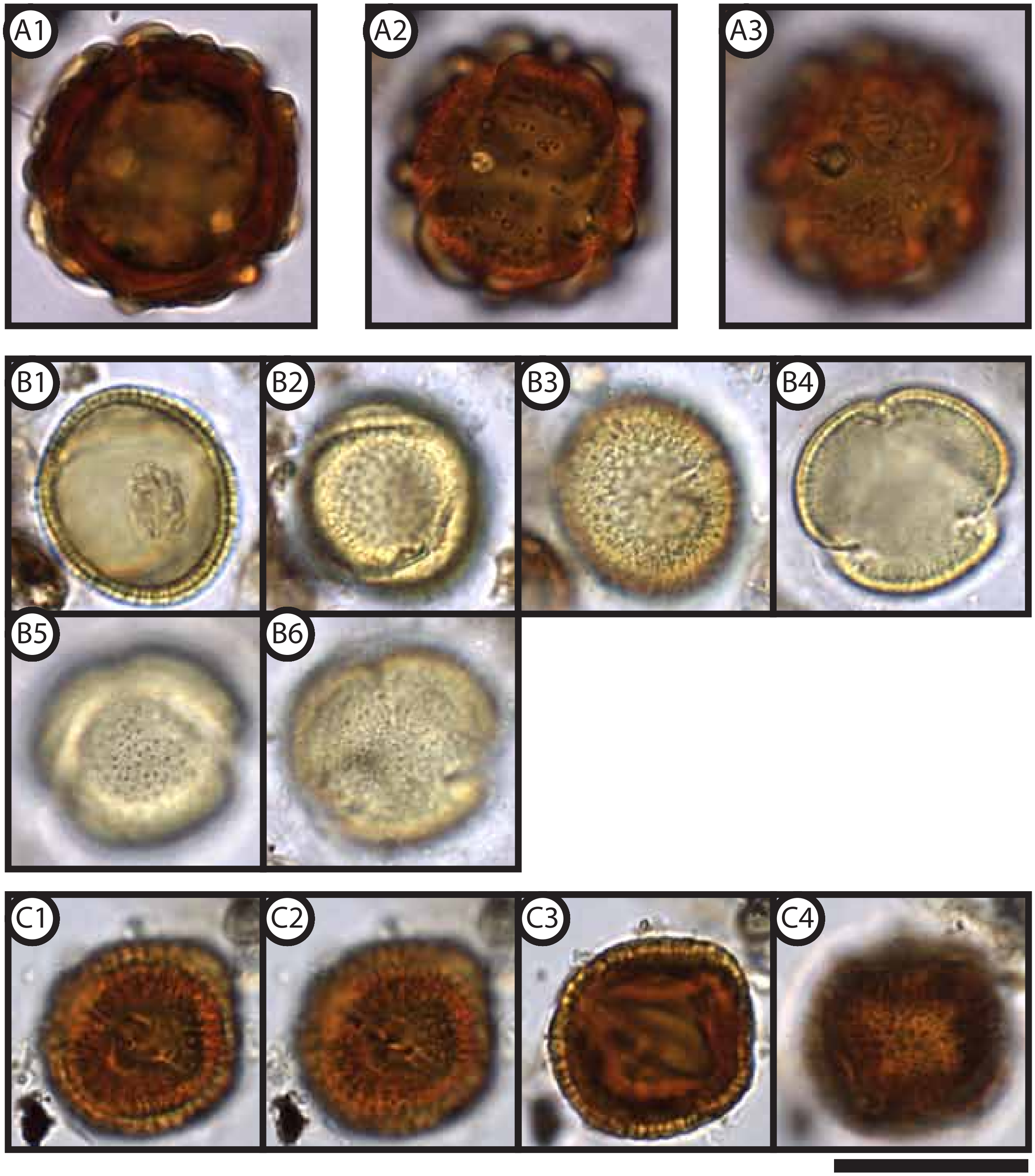

Plate S42. unknown sp./aff. Malpighiaceae: *Tetrapteris* sp. (A1-A3); unknown sp. (B1-B6); unknown sp./aff. Rubiaceae sp. (C1-C4)

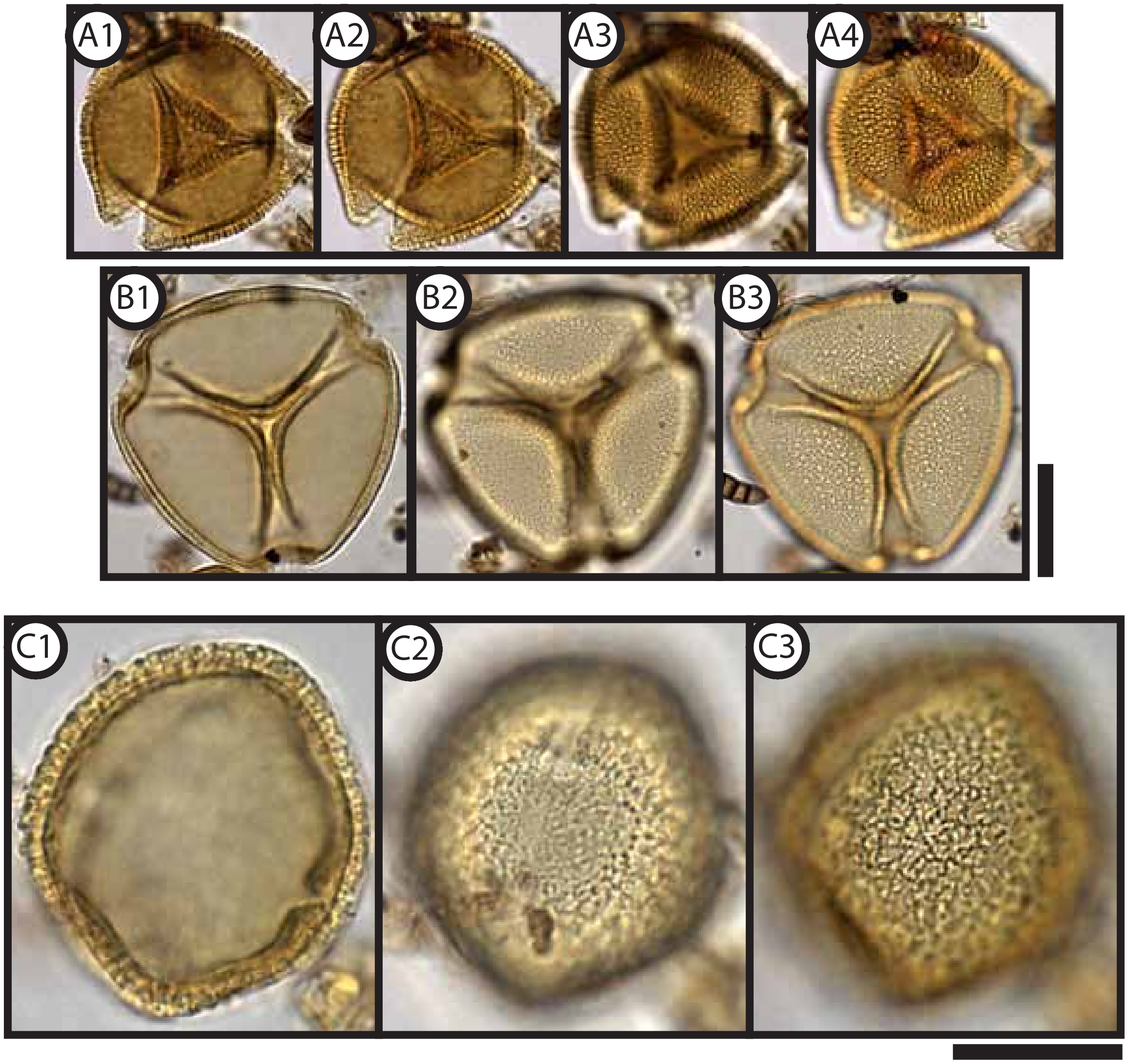

Plate S43. unknown sp. (A1-A4, image scaled 50%);
unknown sp. (B1-B3, image scaled 50%);
unknown sp. (C1-C3, image scaled 75%)

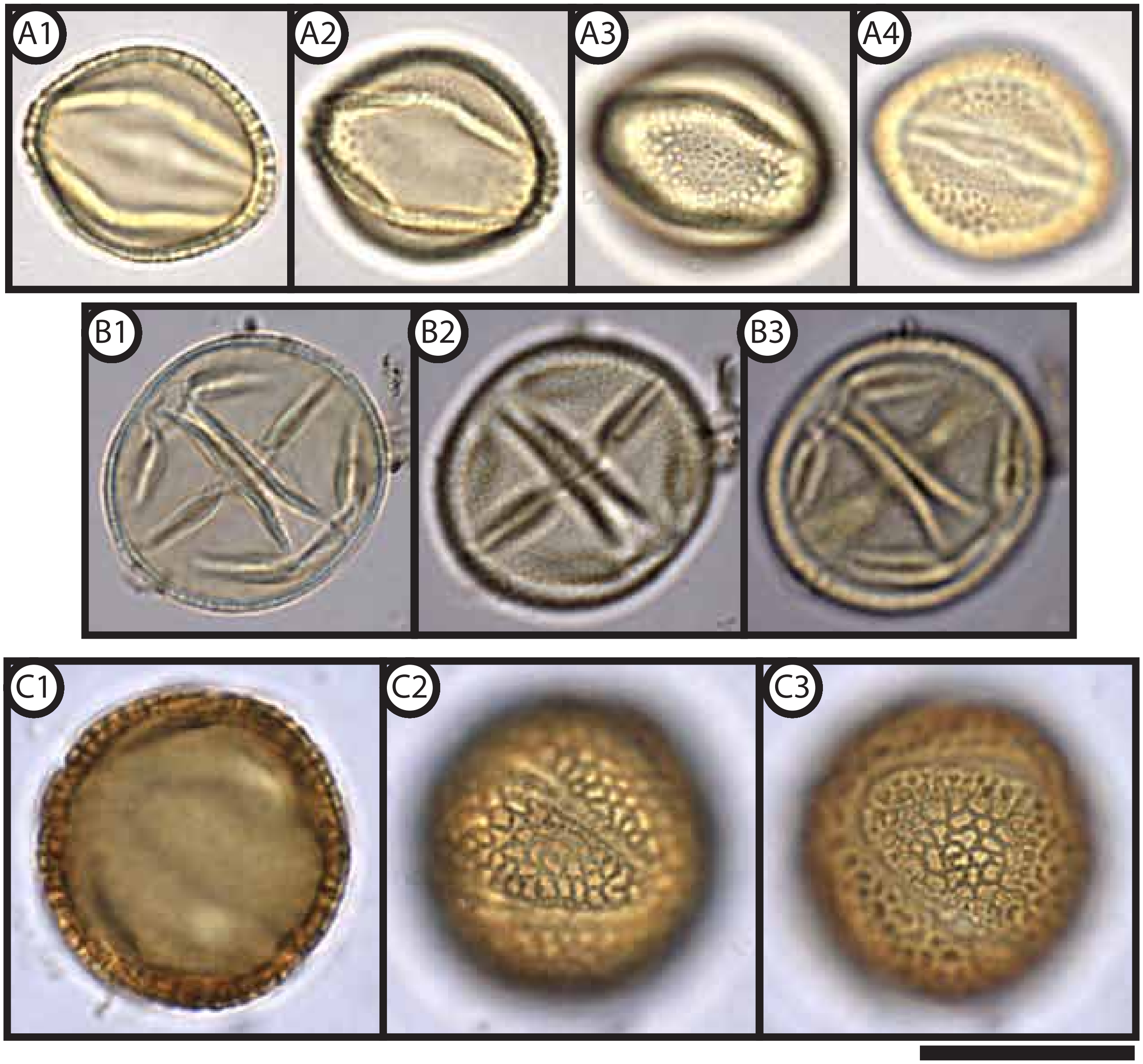

Plate S44. unknown sp. (A1-A4) unknown sp. (B1-B3); unknown sp./cf. Lamiaceae: *Hyptis* sp. (C1-C3)

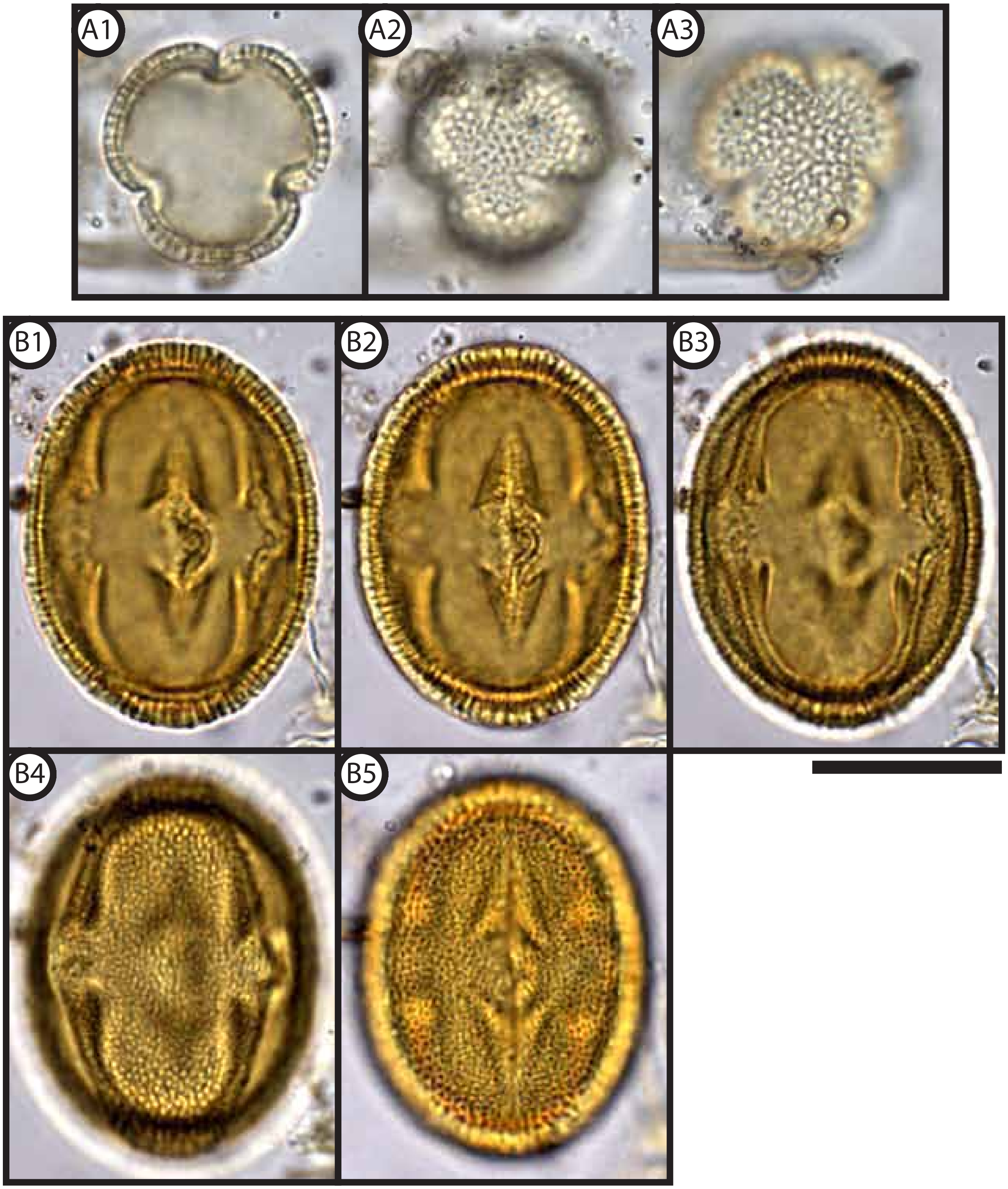

Plate S45. unknown sp. (A1-A3); unknown sp./cf. Sterculiaceae: *Melochia* sp. (B1-B5)
